# Supplementary figures and images for: Mrj is a chaperone of the Hsp40 family that regulates Orb2 oligomerization and long-term memory in Drosophila
Source: PLoS Biol. 2024 Apr 22;22(4):e3002585. doi: 10.1371/journal.pbio.3002585 (PMC11034981; doi:10.1371/journal.pbio.3002585)

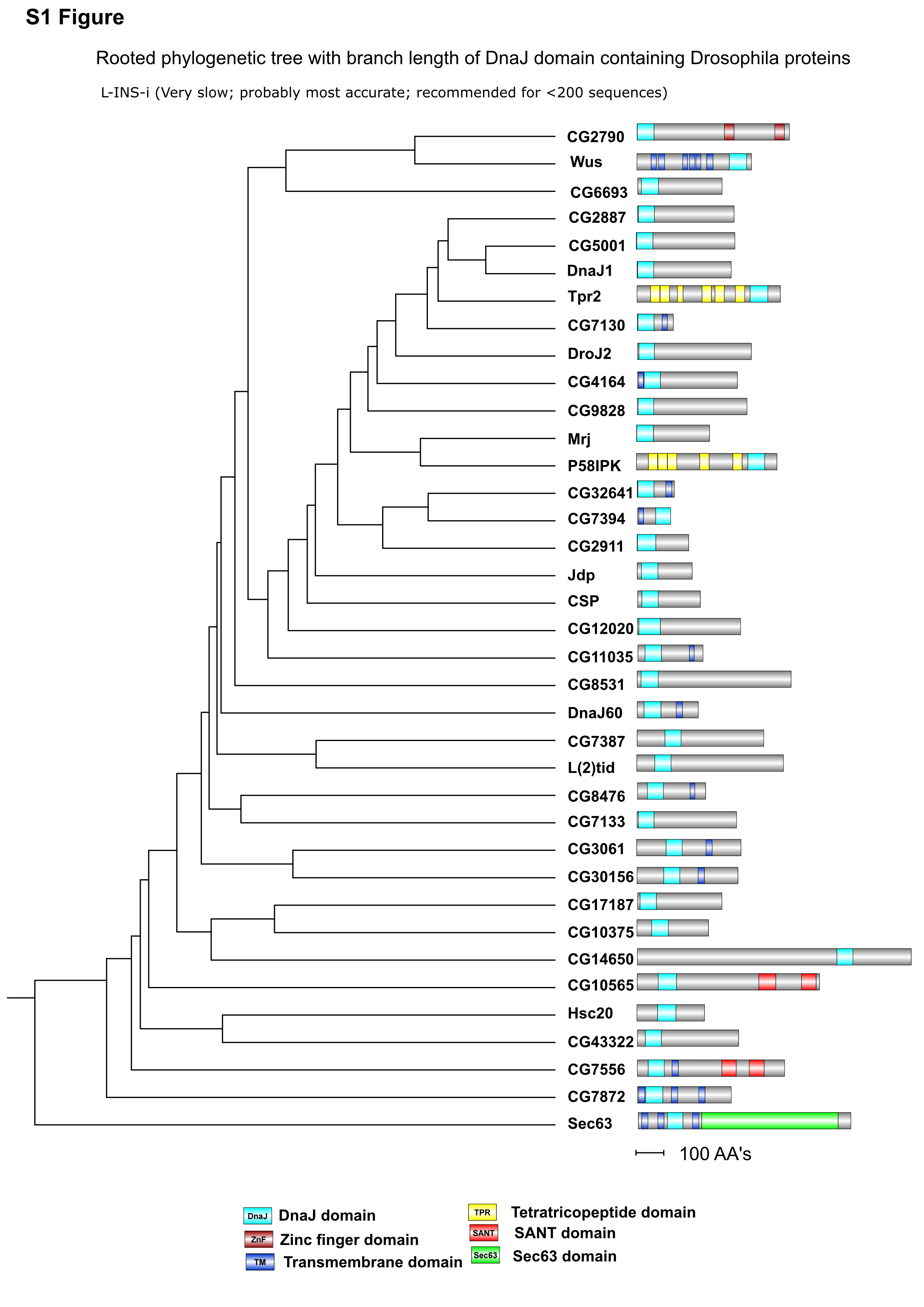

Supplement: S1 Fig — This list does not show Auxillin and Rme8, which were not used in the screen here. (TIFF) [file pbio.3002585.s001.tiff]

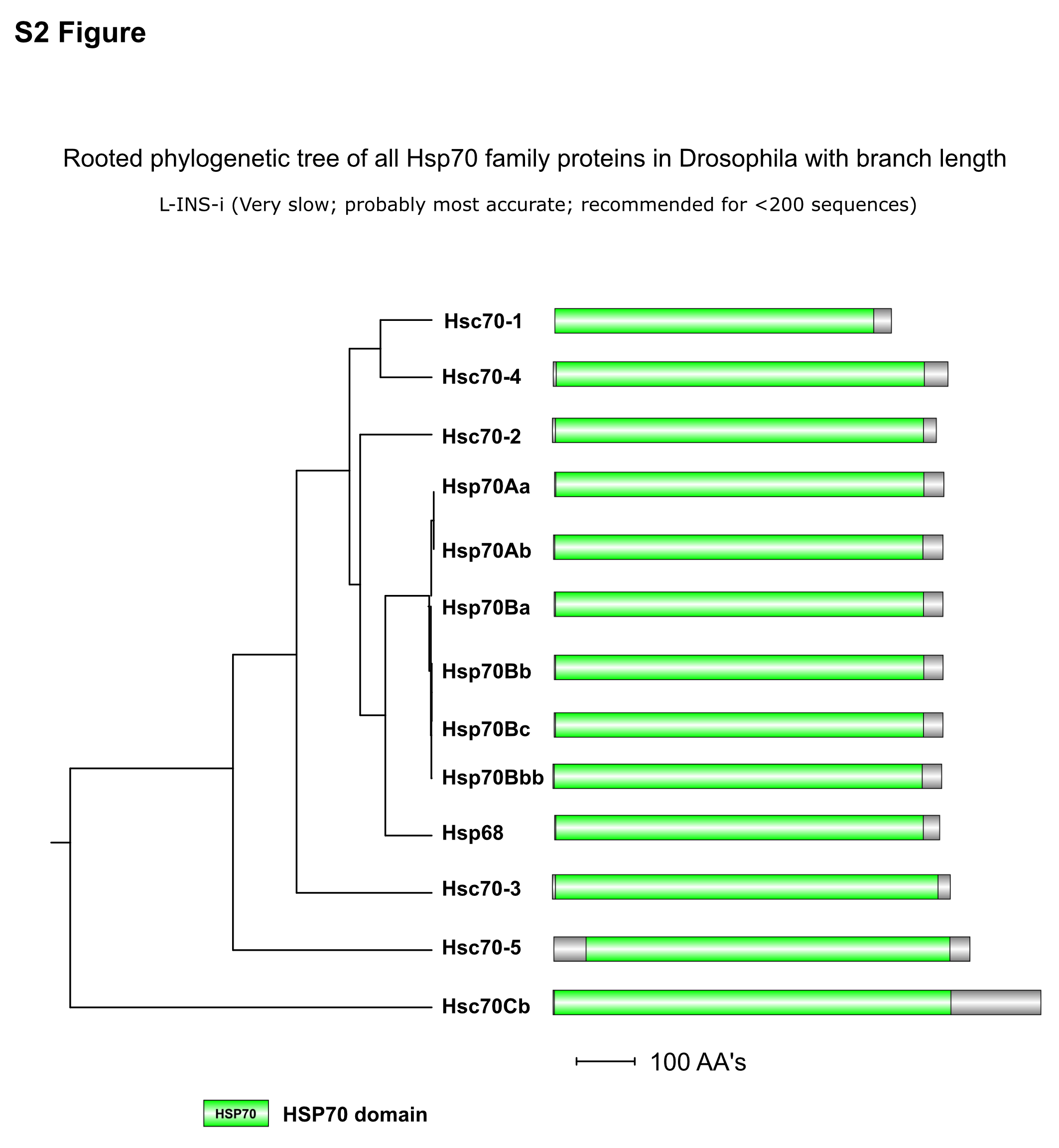

Supplement: S2 Fig — Of these, Hsp70Aa, Hsc70-1, Hsc70Cb, and Hsc70-4 were used in the screen. (TIFF) [file pbio.3002585.s002.tiff]

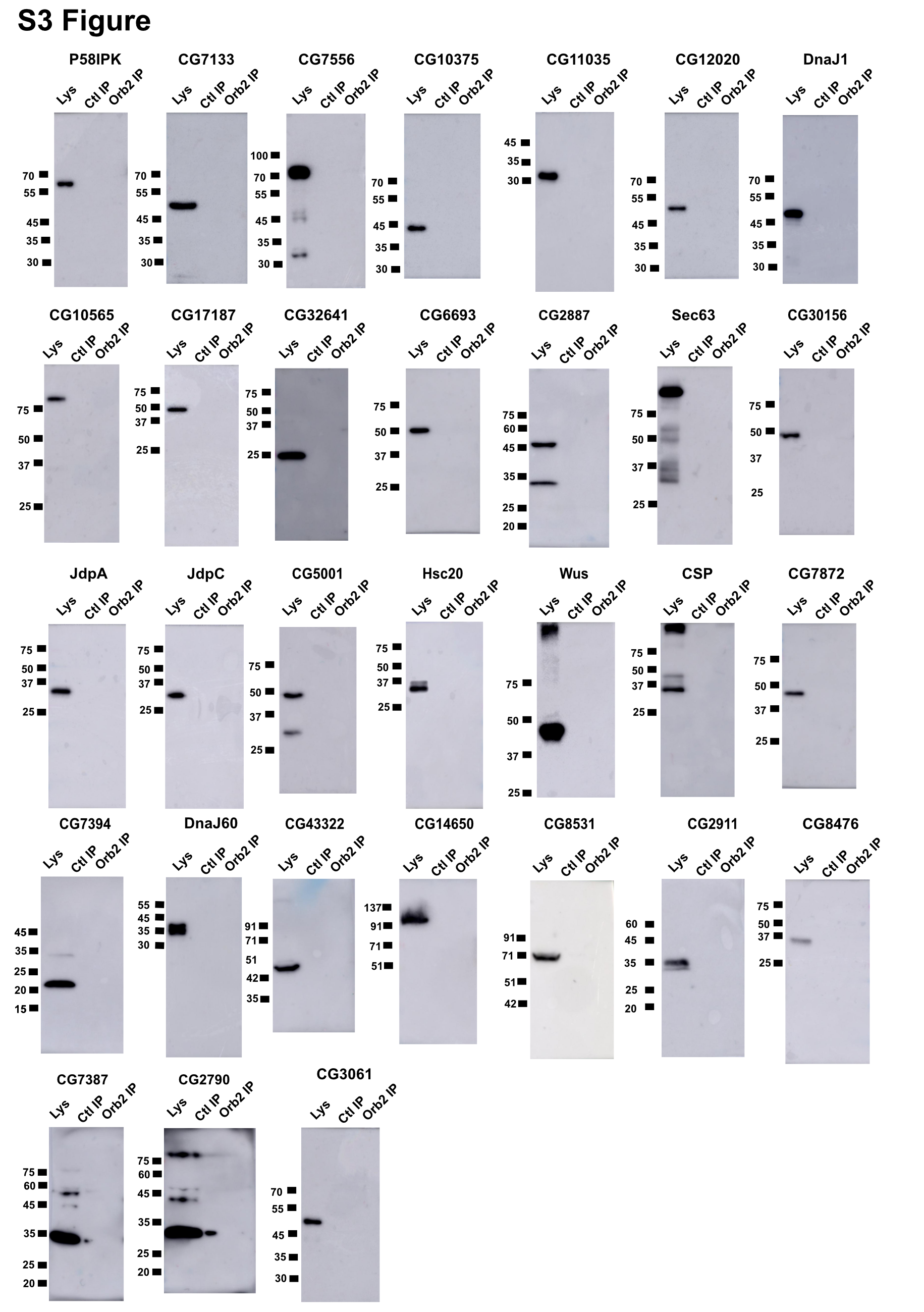

Supplement: S3 Fig — The data underlying this figure are available at: https://figshare.com/s/f5d913a0a289339ee16b. (TIFF) [file pbio.3002585.s003.tiff]

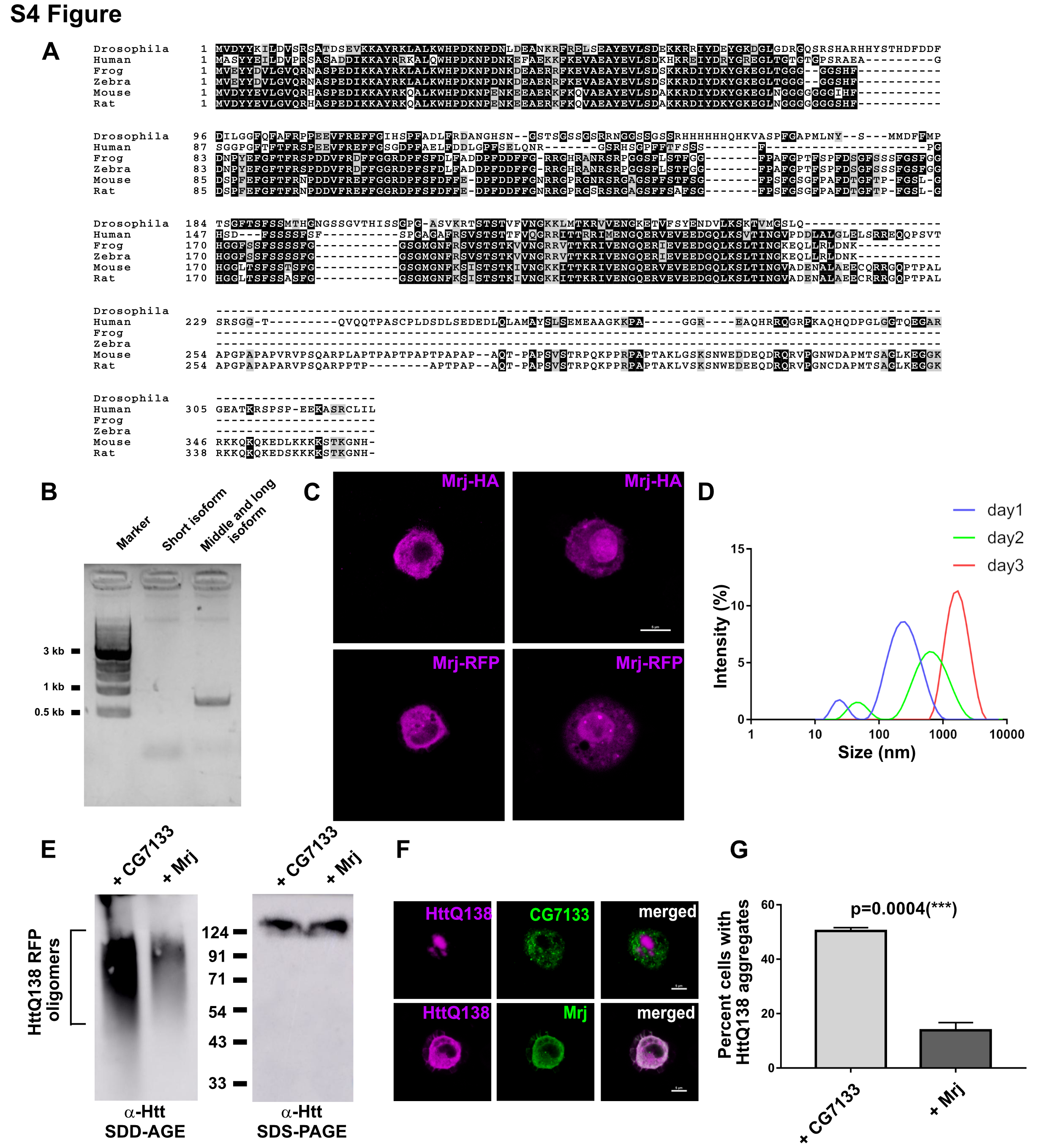

Supplement: S4 Fig — (A) ClustalO alignment of Drosophila Mrj with Human, Frog, Zebrafish, Mouse, and Rat Mrj/DnaJB6. (B) RT-PCR using isoform-specific primer sets shows amplification of only the 777 nucleotides long form which corresponds to 259 amino acid isoform of Mrj. (C) Representative images of S2 cells expressing Mrj-HA (upper panels) and Mrj-RFP (lower panels). Both constructs show the presence of Mrj in both the nucleus and cytoplasm. Scale bar is of 5 microns. (D) DLS experiments with recombinant Mrj over 3 days show its shift to higher sizes with time. (E) Left panel shows a representative SDD-AGE from S2 cell lysate coexpressing HttQ138-RFP along with CG7133 and Mrj showed a decreased amount of Htt oligomers in presence of Mrj. The right panel is of a western blot of lysates in SDS-PAGE from S2 cells coexpressing HttQ138-RFP with Mrj and CG7133 showing similar amounts of Htt. (F) Representative images of HttQ138-RFP cells coexpressing with CG7133-HA and Mrj-HA suggests a decrease in the Htt aggregates in presence of Mrj. Scale bars are of 5 microns. (G) Quantitation of the percentage of HttQ138-RFP expressing cells with aggregates in presence of CG7133 and Mrj suggests a significant decrease of Htt aggregates in presence of Mrj. Data is represented as mean ± SEM and significance is checked using two-tailed Student’s paired t test. The data underlying this figure are available at: https://figshare.com/s/f5d913a0a289339ee16b. (TIFF) [file pbio.3002585.s004.tiff]

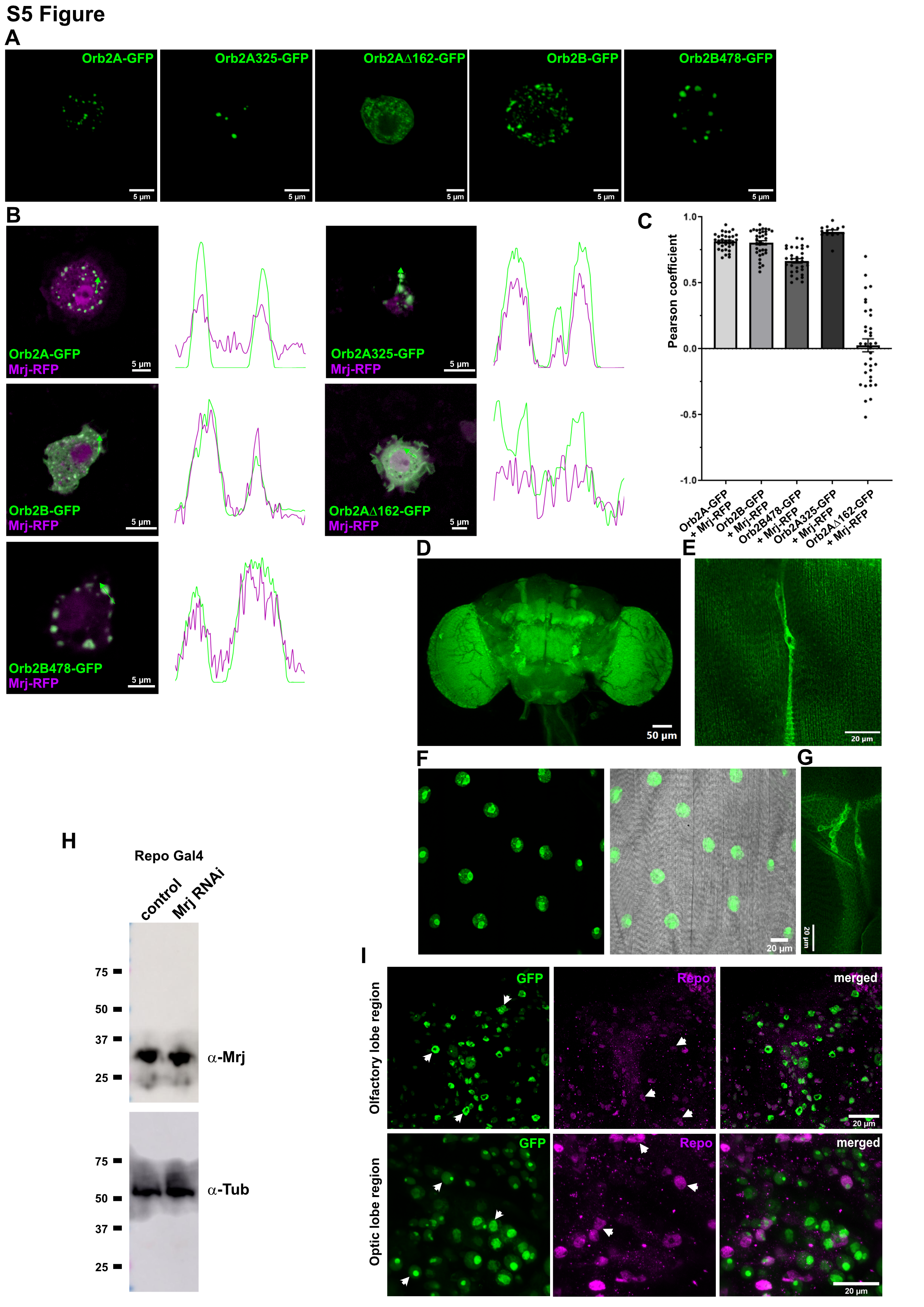

Supplement: S5 Fig — (A) Representative images of S2 cells expressing Orb2A, Orb2A325, Orb2AΔ162, Orb2B, and Orb2B478 constructs tagged with GFP. (B) Intensity profile plots from lines drawn over punctae in the cells show colocalization between Orb2A-GFP, Orb2A325-GFP, Orb2AΔ162-GFP, Orb2B-GFP, and Orb2B478-GFP with Mrj-RFP. The Orb2AΔ162-GFP construct does not show colocalization with Mrj-RFP. (C) Colocalization quantitation using Pearson’s coefficient shows significant colocalization of Mrj-RFP with Orb2AGFP (n = 34 ROI’s from 4 cells), Orb2B-GFP (n = 36 ROI’s from 6 cells), Orb2A325-GFP (n = 14 ROI’s from 5 cells), Orb2B478-GFP (n = 32 ROI’s from 8 cells) but not with Orb2AΔ162-GFP (n = 35 ROI’s from 5 cells). Data is represented as mean ± SEM. (D) Drosophila adult brain image of Mrj knockout (KO) Gal4 driving UAS-CD8GFP showing enriched expression in optic lobes, olfactory lobes, and the mushroom body region. Scale bar is of 50 microns. (E) Image from the larval muscle of the same flies show expression of CD8-GFP in the muscle. Scale bar is 20 microns. (F) Image of larval muscles from Mrj KO Gal4 driving UAS-NLS-GFP shows the expression in multiple nuclei confirming the expression in muscle fibers. The right panel depicts the merge of NLS-GFP with the DIC image. Scale bar is 20 micron. (G) Image of the neuromuscular junction from larvae of Mrj KO Gal4 driving UAS-CD8GFP shows expression in the neuromuscular junction synaptic boutons. Scale bar is 20 micron. (H) Driving the Mrj RNAi line in Glial cells using Repo Gal4 shows no difference in Mrj expression in comparison to the control animals as seen in the western blot with anti-Mrj antibody. Anti-α-Tubulin antibody was used as the loading control. (I) Representative images from olfactory and optic lobe regions of Mrj KO Gal4 driving NLS-GFP brains immunostained with glia-specific anti-Repo antibody. Arrows in the GFP panel depict cells with only GFP and no Repo expression. Arrows in the Repo panel depict cells with both GFP and Repo [file pbio.3002585.s005.tiff]

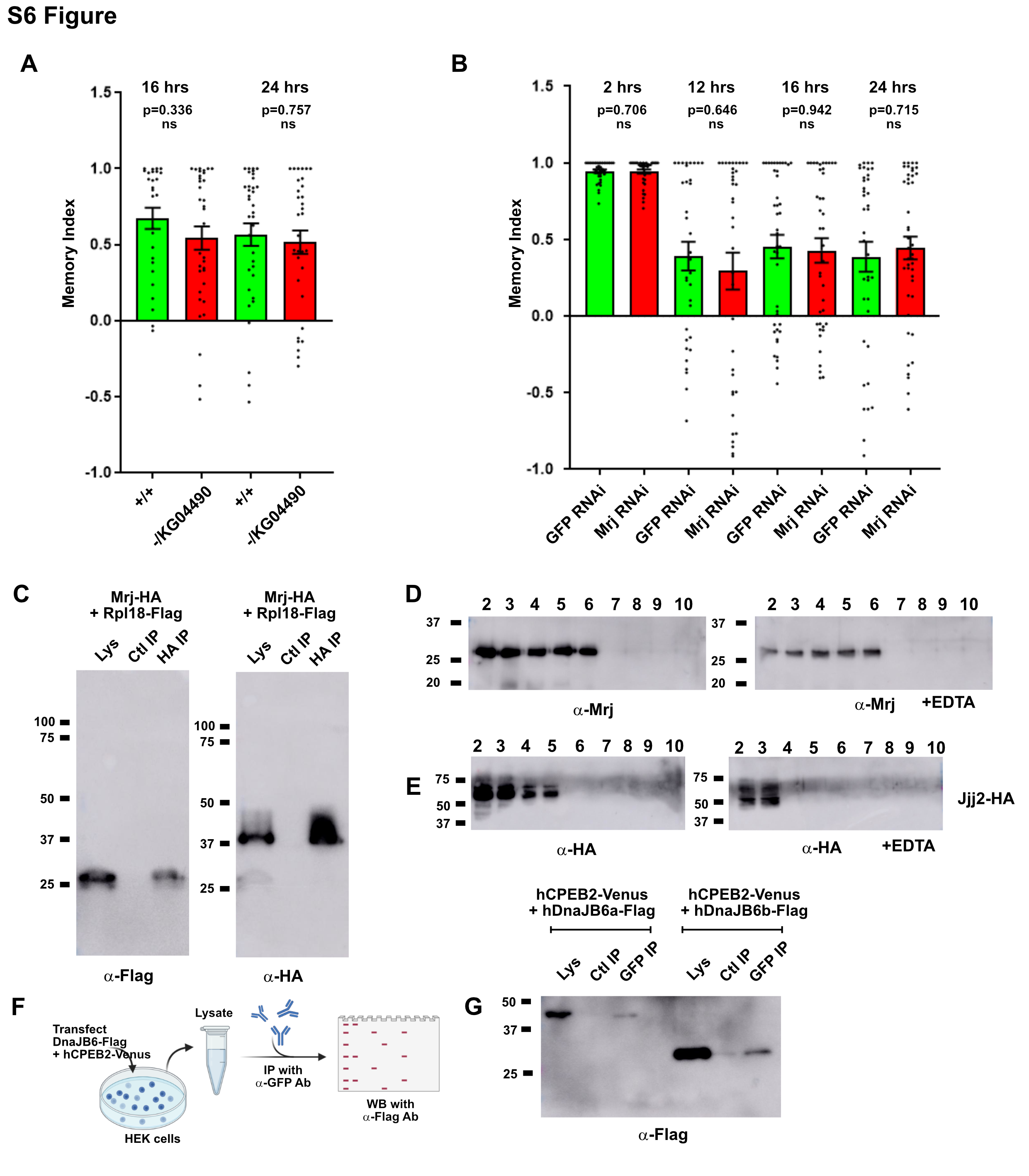

Supplement: S6 Fig — (A) Mrj-/KG04490 flies do not show any significant memory deficit in comparison to the wild-type flies at 16 and 24 h onwards. Data are represented as mean ± SEM and Mann–Whitney U test is done to test for significance. (B) Preventing the knockdown of Mrj in specific mushroom body neurons using 201Y Gal4 with Tub Gal80 ts at 18°C does not cause any significant memory deficit in comparison to the control from 16 h onwards. Data are represented as mean ± SEM and Mann–Whitney U test is done to test for significance. (C) Immunoprecipitated Mrj-HA pulls down Rpl18-Flag which is incorporated in ribosomes, suggesting Mrj is associated with ribosomes. The right panel shows the same blot probed with anti-HA antibody and confirming the presence of Mrj-HA in the immunoprecipitate. (D) Loading recombinant Mrj on an identical polysome gradient followed by centrifugation and fractionation and probing these fractions with anti-Mrj antibody shows their absence from the heavier polysome fractions. Also, on EDTA treatment the recombinant Mrj does not move to the heavier fractions, unlike our observations with cellularly expressed Mrj. (E) Polysome fractionation of Jjj2-HA expressing S2 cells in the absence and presence of EDTA followed by detection of Jjj2 using western blots show its absence in the heavier polysome fractions. (F) Schematic of immunoprecipitation assay to check the possibility of interaction between hCPEB2 and hDnaJB6 in HEK cells. The cells were lysed and immunoprecipitation was done with anti-GFP antibody (GFP-Trap beads). The immunoprecipitate was next probed with anti-Flag antibody. (G) Representative image of the blot shows a pulldown of DnaJB6 by hCPEB2. The data underlying this figure are available at: https://figshare.com/s/f5d913a0a289339ee16b. (TIFF) [file pbio.3002585.s006.tiff]

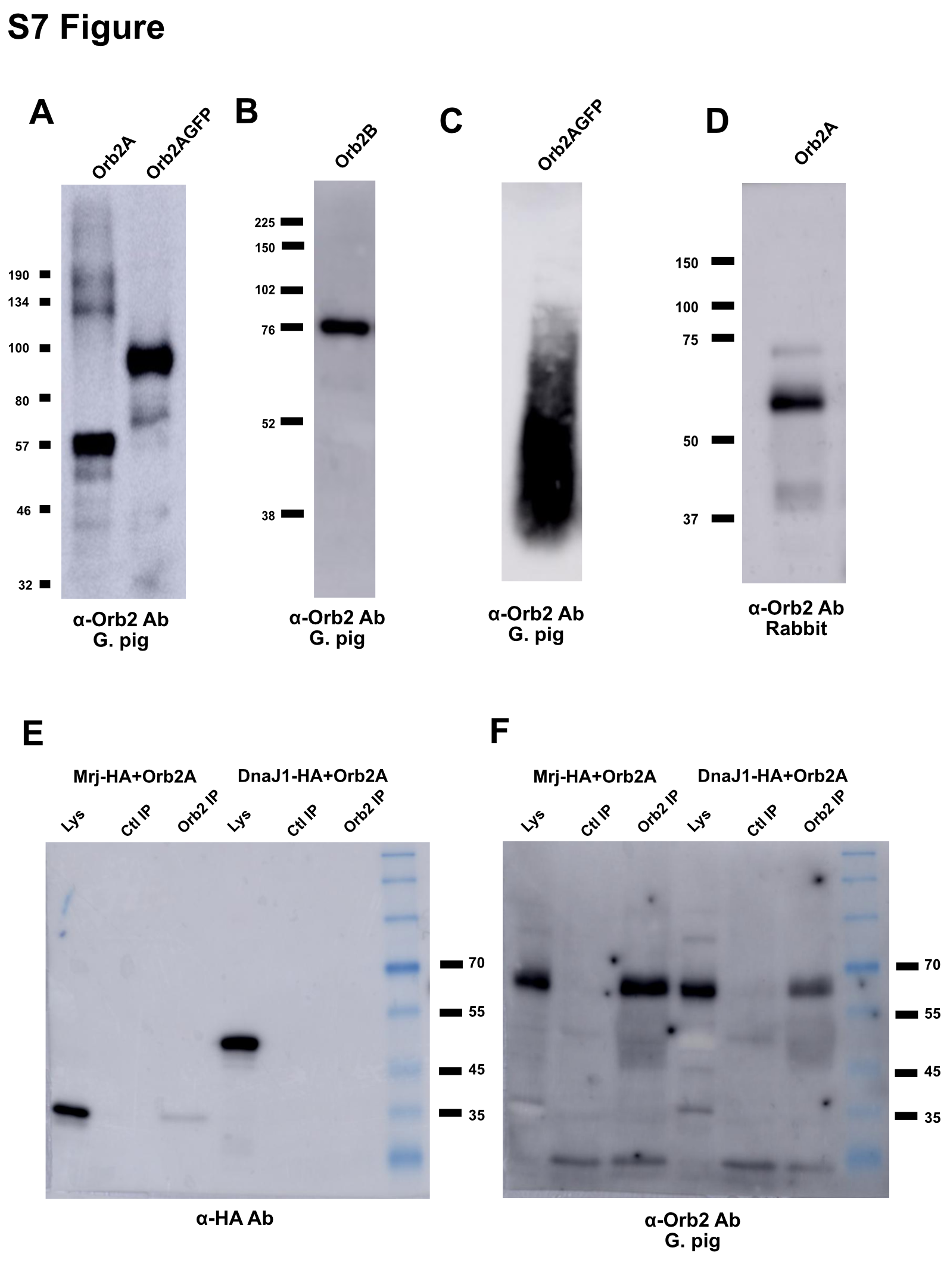

Supplement: S7 Fig — (A) Validation of anti Orb2 antibody raised in guinea pig in western blot with S2 cell lysate from cells expressing Orb2A and Orb2A-GFP. (B) Guinea pig anti-Orb2 antibody detects endogenous Orb2B from fly head extract. (C) Guinea pig anti-Orb2 antibody detects Orb2A-GFP oligomers from Sf9 cell lysate. (D) Validation of anti Orb2 antibody raised in rabbit in western blot with S2 cell lysate from cells expressing Orb2A. (E) Representative western blot of immunoprecipitation performed from cells expressing Orb2A with Mrj-HA and Orb2A with DnaJ1-HA with anti Orb2 antibody. Probing the blot with an anti-HA antibody shows the presence of Mrj in the lane for lysate and Orb2 IP suggesting its interaction with Orb2A. In contrast for DnaJ1, it is detected only in the lysate lane and not in the Orb2 IP lane, suggesting no interaction between DnaJ1 and Orb2A. (F) The same blot as in E was probed with anti Orb2 antibody and here Orb2A could be detected in both the lysate lanes and Orb2 IP lanes confirming the pull down of Orb2A with anti Orb2 antibody. The data underlying this figure are available at: https://figshare.com/s/f5d913a0a289339ee16b. (TIFF) [file pbio.3002585.s007.tiff]
