# Supplementary material for: Mrj is a chaperone of the Hsp40 family that regulates Orb2 oligomerization and long-term memory in Drosophila
Source: PLoS Biol. 2024 Apr 22;22(4):e3002585. doi: 10.1371/journal.pbio.3002585 (PMC11034981; doi:10.1371/journal.pbio.3002585)
Supplement: S1 Data — (DOCX) [file pbio.3002585.s008.docx]

**S1_data:**

**Clustal O analysis of all Drosophila Mrj isoforms**

**Mrj-PA MVDYYKILDVSRSATDSEVKKAYRKLALKWHPDKNPDNLDEANKRFRELSEAYEVLSDEK
Mrj-PB MVDYYKILDVSRSATDSEVKKAYRKLALKWHPDKNPDNLDEANKRFRELSEAYEVLSDEK
Mrj-PC MVDYYKILDVSRSATDSEVKKAYRKLALKWHPDKNPDNLDEANKRFRELSEAYEVLSDEK
Mrj-PD MVDYYKILDVSRSATDSEVKKAYRKLALKWHPDKNPDNLDEANKRFRELSEAYEVLSDEK
Mrj-PE MVDYYKILDVSRSATDSEVKKAYRKLALKWHPDKNPDNLDEANKRFRELSEAYEVLSDAR
Mrj-PG MVDYYKILDVSRSATDSEVKKAYRKLALKWHPDKNPDNLDEANKRFRELSEAYEVLSDAR
Mrj-PH MVDYYKILDVSRSATDSEVKKAYRKLALKWHPDKNPDNLDEANKRFRELSEAYEVLSDAR
Mrj-PF ------------------------------------------------------------

Mrj-PA ------------------------------------------------------------
Mrj-PB ------------------------------------------------------------
Mrj-PC ------------------------------------------------------------
Mrj-PD ------------------------------------------------------------
Mrj-PE KRRIYDARATLHKSSNSGSSSSNSSSYTRYRNGGTGGSSSYGRDYDYDYYPGSGYGSGSG
Mrj-PG KRRIYDARATLHKSSNSGSSSSNSSSYTRYRNGGTGGSSSYGRDYDYDYYPGSGYGSGSG
Mrj-PH KRRIYDARATLHKSSNSGSSSSNSSSYTRYRNGGTGGSSSYGRDYDYDYYPGSGYGSGSG
Mrj-PF ------------------------------------------------------------

Mrj-PA ---------------------------KRRIYDEYGKDGLGDRGQSRSHARHHYSTHDFD
Mrj-PB ---------------------------KRRIYDEYGKDGLGDRGQSRSHARHHYSTHDFD
Mrj-PC ---------------------------KRRIYDEYGKDGLGDRGQSRSHARHHYSTHDFD
Mrj-PD ---------------------------KRRIYDEYGKDGLGDRGQSRSHARHHYSTHDFD
Mrj-PE RRSGNRYQAFTFRNIFEGTPFHKMFEKKRRIYDEYGKDGLGDRGQSRSHARHHYSTHDFD
Mrj-PG RRSGNRYQAFTFRNIFEGTPFHKMFEKKRRIYDEYGKDGLGDRGQSRSHARHHYSTHDFD
Mrj-PH RRSGNRYQAFTFRNIFEGTPFHKMFEKKRRIYDEYGKDGLGDRGQSRSHARHHYSTHDFD
Mrj-PF ------------------MYSHFLTEKKRRIYDEYGKDGLGDRGQSRSHARHHYSTHDFD

Mrj-PA DFDILGGFQFAFRPPEEVFREFFGIHSPFADLFRDANGHSNGSTSGSSGSRRNGGSSGSS
Mrj-PB DFDILGGFQFAFRPPEEVFREFFGIHSPFADLFRDANGHSNGSTSGSSGSRRNGGSSGSS
Mrj-PC DFDILGGFQFAFRPPEEVFREFFGIHSPFADLFRDANGHSNGSTSGSSGSRRNGGSSGSS
Mrj-PD DFDILGGFQFAFRPPEEVFREFFGIHSPFADLFRDANGHSNGSTSGSSGSRRNGGSSGSS
Mrj-PE DFDILGGFQFAFRPPEEVFREFFGIHSPFADLFRDANGHSNGSTSGSSGSRRNGGSSGSS
Mrj-PG DFDILGGFQFAFRPPEEVFREFFGIHSPFADLFRDANGHSNGSTSGSSGSRRNGGSSGSS
Mrj-PH DFDILGGFQFAFRPPEEVFREFFGIHSPFADLFRDANGHSNGSTSGSSGSRRNGGSSGSS
Mrj-PF DFDILGGFQFAFRPPEEVFREFFGIHSPFADLFRDANGHSNGSTSGSSGSRRNGGSSGSS

Mrj-PA RHHHHHHQHKVASPFGAPMLNYSMMDFFMPTSGFTSFSSMTHGNGSSGVTHISSGPGASV
Mrj-PB RHHHHHHQHKVASPFGAPMLNYSMMDFFMPTSGFTSFSSMTHGNGSSGVTHISSGPGASV
Mrj-PC RHHHHHHQHKVASPFGAPMLNYSMMDFFMPTSGFTSFSSMTHGNGSSGVTHISSGPGASV
Mrj-PD RHHHHHHQHKVASPFGAPMLNYSMMDFFMPTSGFTSFSSMTHGNGSSGVTHISSGPGASV
Mrj-PE RHHHHHHQHKVASPFGAPMLNYSMMDFFMPTSGFTSFSSMTHGNGSSGVTHISSGPGASV
Mrj-PG RHHHHHHQHKVASPFGAPMLNYSMMDFFMPTSGFTSFSSMTHGNGSSGVTHISSGPGASV
Mrj-PH RHHHHHHQHKVASPFGAPMLNYSMMDFFMPTSGFTSFSSMTHGNGSSGVTHISSGPGASV
Mrj-PF RHHHHHHQHKVASPFGAPMLNYSMMDFFMPTSGFTSFSSMTHGNGSSGVTHISSGPGASV

Mrj-PA KRTSTSTVFVNGKKLMTKRVVENGKETVFSYENDVLKSKTVMGSLQ
Mrj-PB KRTSTSTVFVNGKKLMTKRVVENGKETVFSYENDVLKSKTVMGSLQ
Mrj-PC KRTSTSTVFVNGKKLMTKRVVENGKETVFSYENDVLKSKTVMGSLQ
Mrj-PD KRTSTSTVFVNGKKLMTKRVVENGKETVFSYENDVLKSKTVMGSLQ
Mrj-PE KRTSTSTVFVNGKKLMTKRVVENGKETVFSYENDVLKSKTVMGSLQ
Mrj-PG KRTSTSTVFVNGKKLMTKRVVENGKETVFSYENDVLKSKTVMGSLQ
Mrj-PH KRTSTSTVFVNGKKLMTKRVVENGKETVFSYENDVLKSKTVMGSLQ
Mrj-PF KRTSTSTVFVNGKKLMTKRVVENGKETVFSYENDVLKSKTVMGSLQ**
