# Supplementary material for: Mrj is a chaperone of the Hsp40 family that regulates Orb2 oligomerization and long-term memory in Drosophila
Source: PLoS Biol. 2024 Apr 22;22(4):e3002585. doi: 10.1371/journal.pbio.3002585 (PMC11034981; doi:10.1371/journal.pbio.3002585)
Supplement: S2 Data — (DOCX) [file pbio.3002585.s009.docx]

**S2_Data:**

**Human homologs of other DnaJ interactors of Orb2**

We have performed BlastP analysis of CG4164, CG9828, CG7130, DroJ2, and Tpr2 protein sequences against Human proteins. Based on this we have listed the highest-ranking candidate identified here for each of these genes.

| **Drosophila Gene** | **Human gene** | **Query cover** | **Percent identity** | **E value** |
| --- | --- | --- | --- | --- |
| CG4164 | dnaJ homolog subfamily B member 11 isoform 1 | 98 % | 62.96% | 2e-150 |
| CG9828 | dnaJ homolog subfamily A member 2 | 92% | 39.41% | 3e-84 |
| CG7130 | dnaJ homolog subfamily B member 4 isoform d | 56% | 69.44% | 2e-30 |
| Tpr2 | dnaJ homolog subfamily C member 7 isoform 1 | 93% | 46.22% | 6e-139 |
| DroJ2 | dnaJ homolog subfamily A member 4 isoform 2 | 98% | 60.60% | 2e-169 |
