# Supplementary material for: Mrj is a chaperone of the Hsp40 family that regulates Orb2 oligomerization and long-term memory in Drosophila
Source: PLoS Biol. 2024 Apr 22;22(4):e3002585. doi: 10.1371/journal.pbio.3002585 (PMC11034981; doi:10.1371/journal.pbio.3002585)
Supplement: S1 Raw Images — (PDF) [file pbio.3002585.s010.pdf]

Figure 1

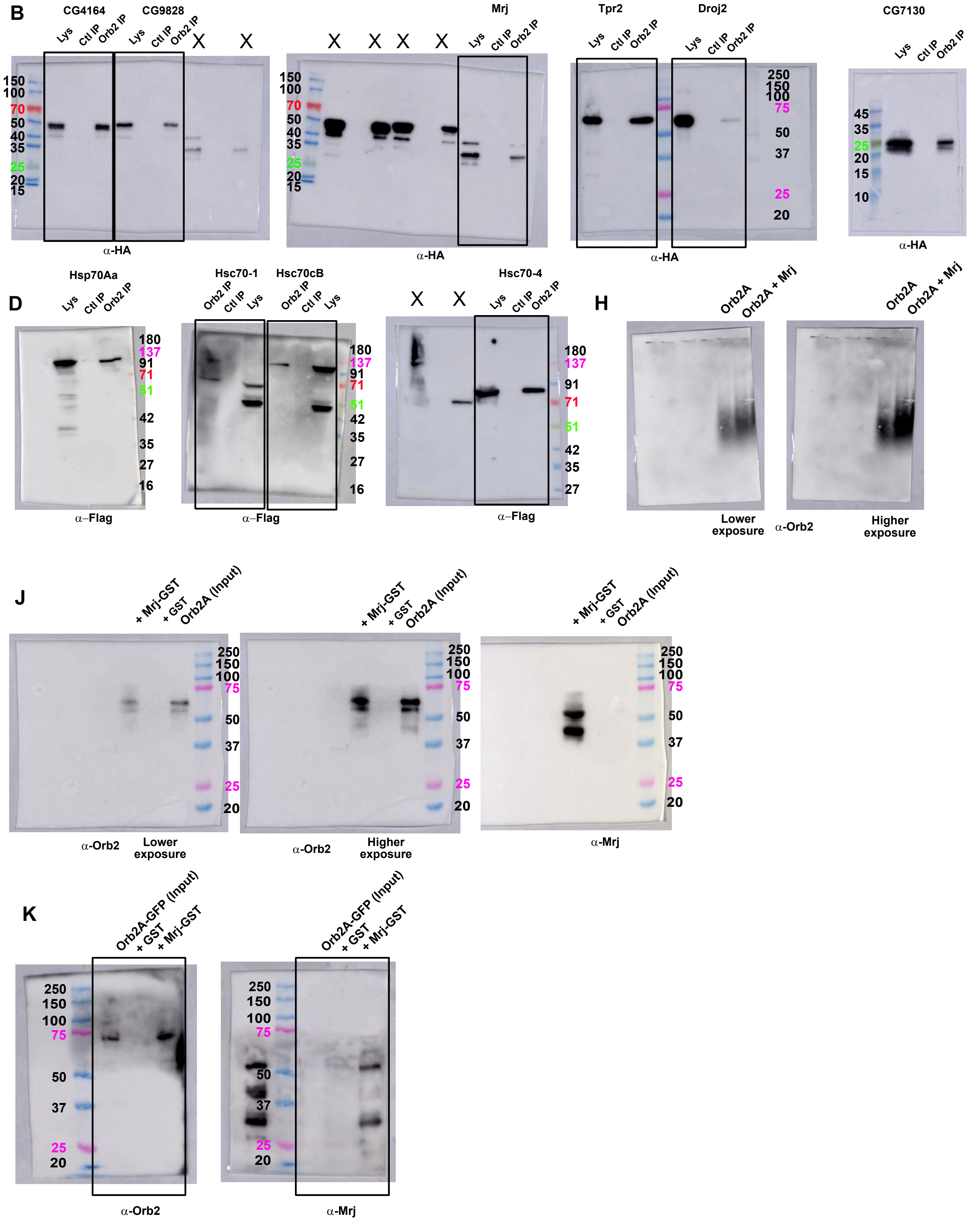

All blots scanned in GE Imager 600

Figure 2

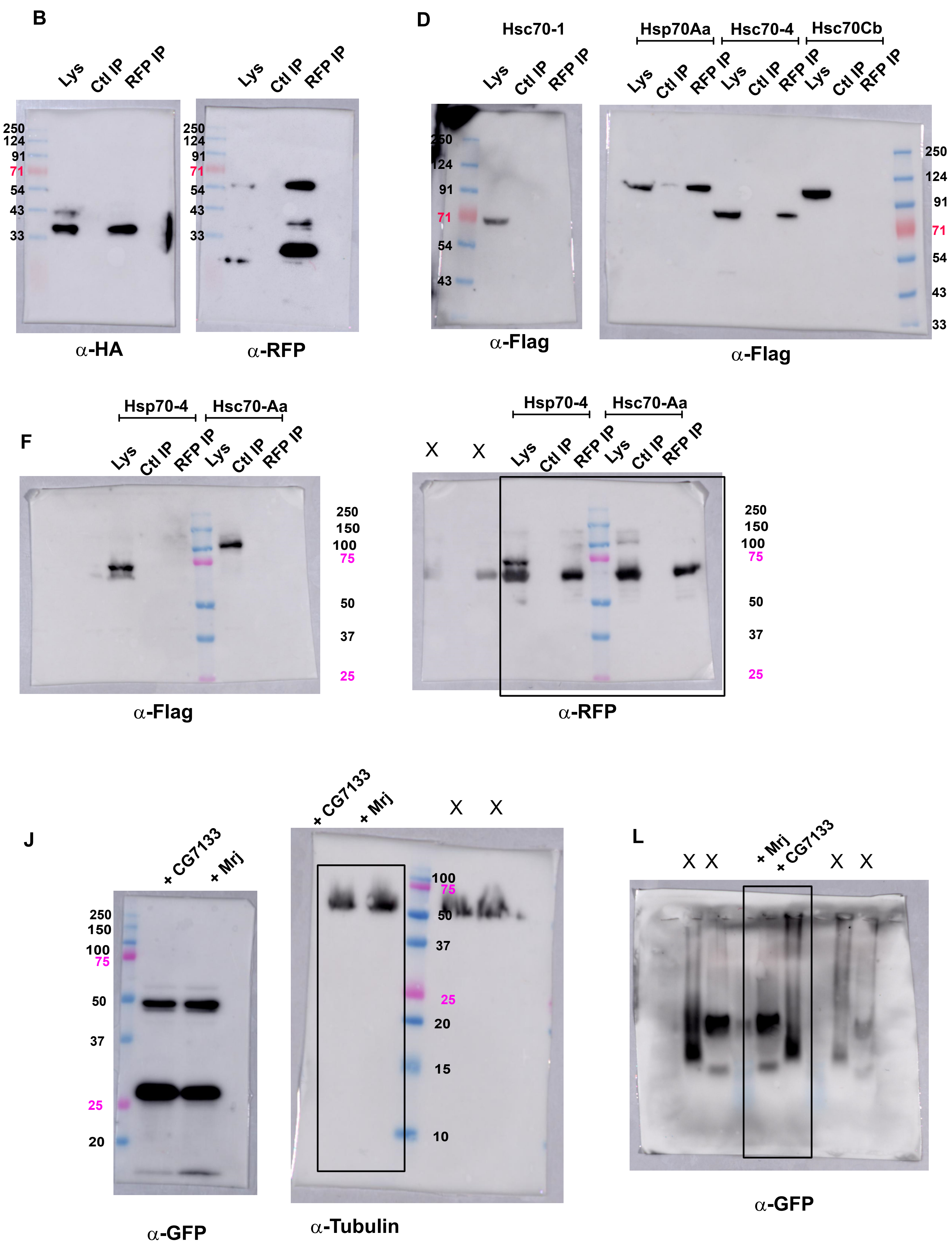

All blots scanned in GE Imager 600

Figure 3

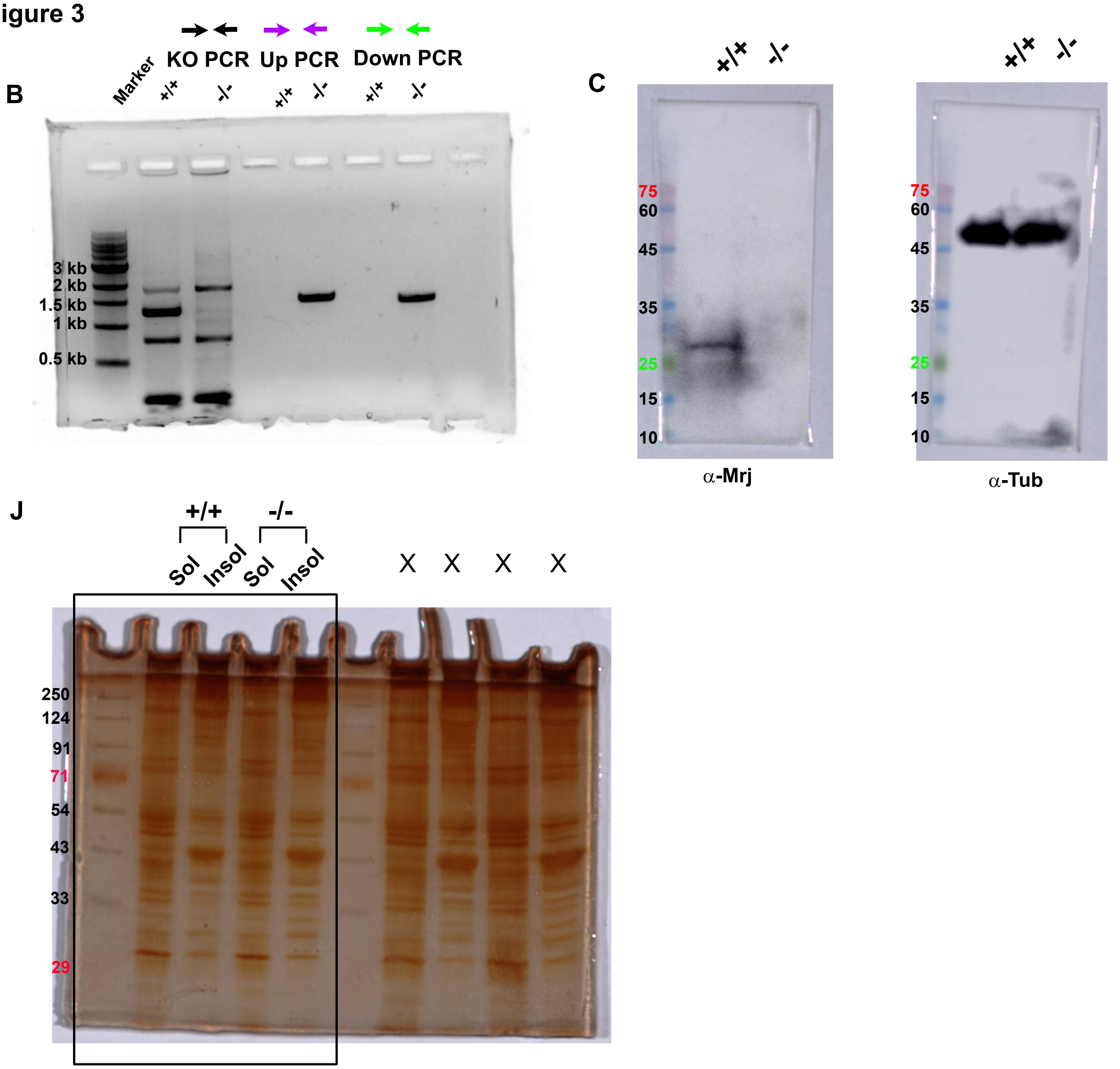

All blots and agarose gel scanned in GE Imager 600

Figure 4

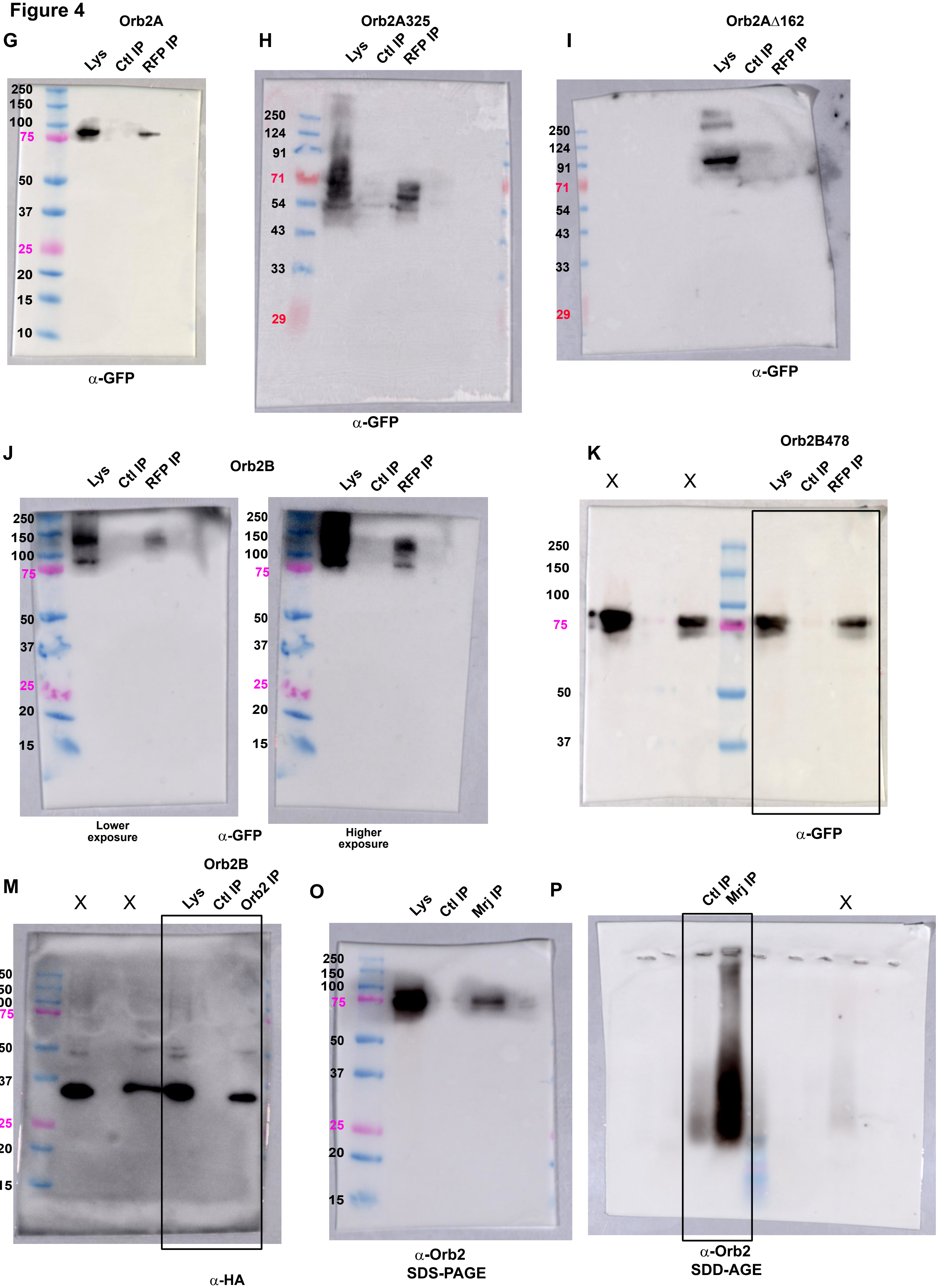

All blots scanned in GE Imager 600

Figure 5

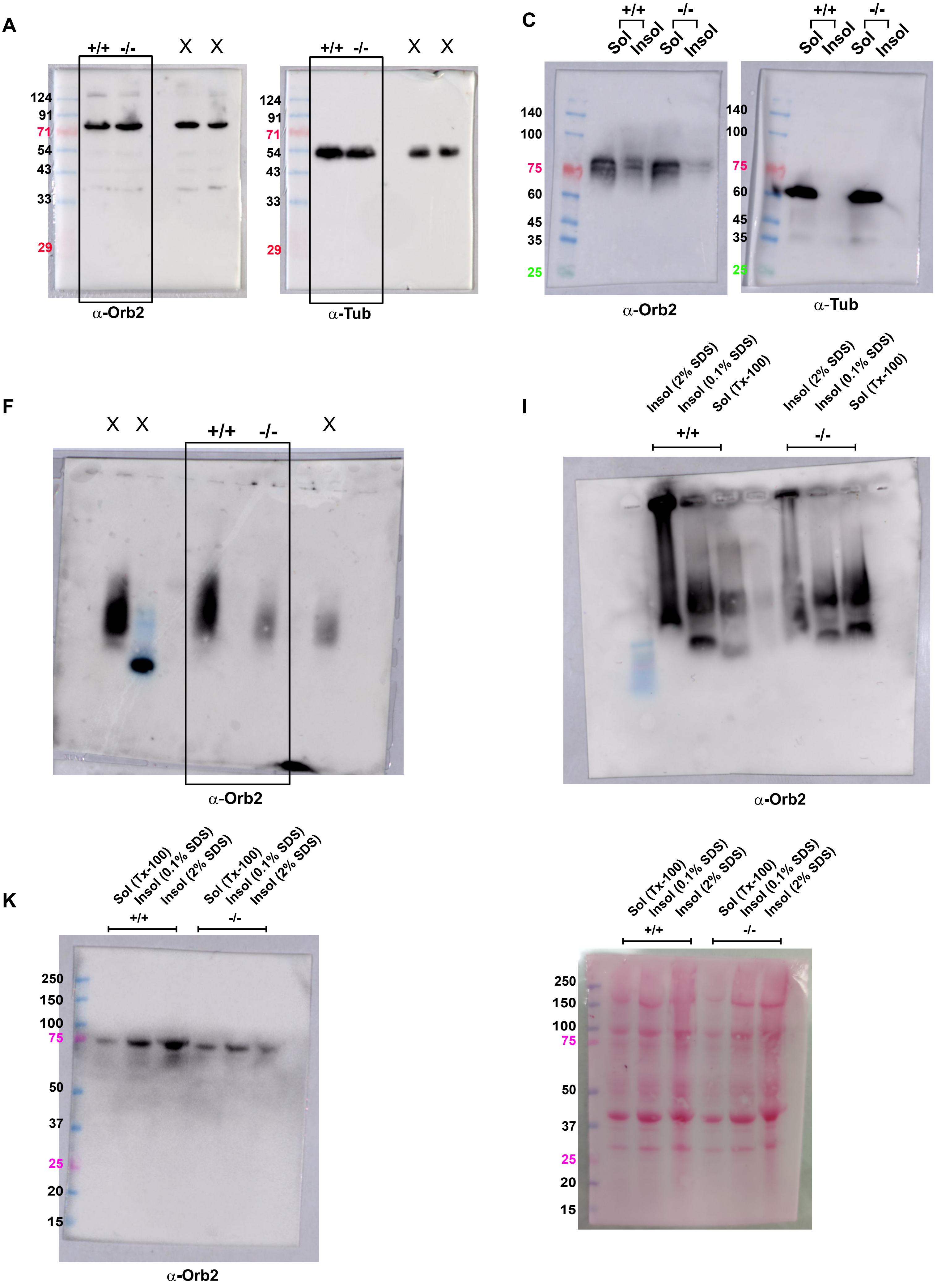

All blots scanned in GE Imager 600

Figure 6

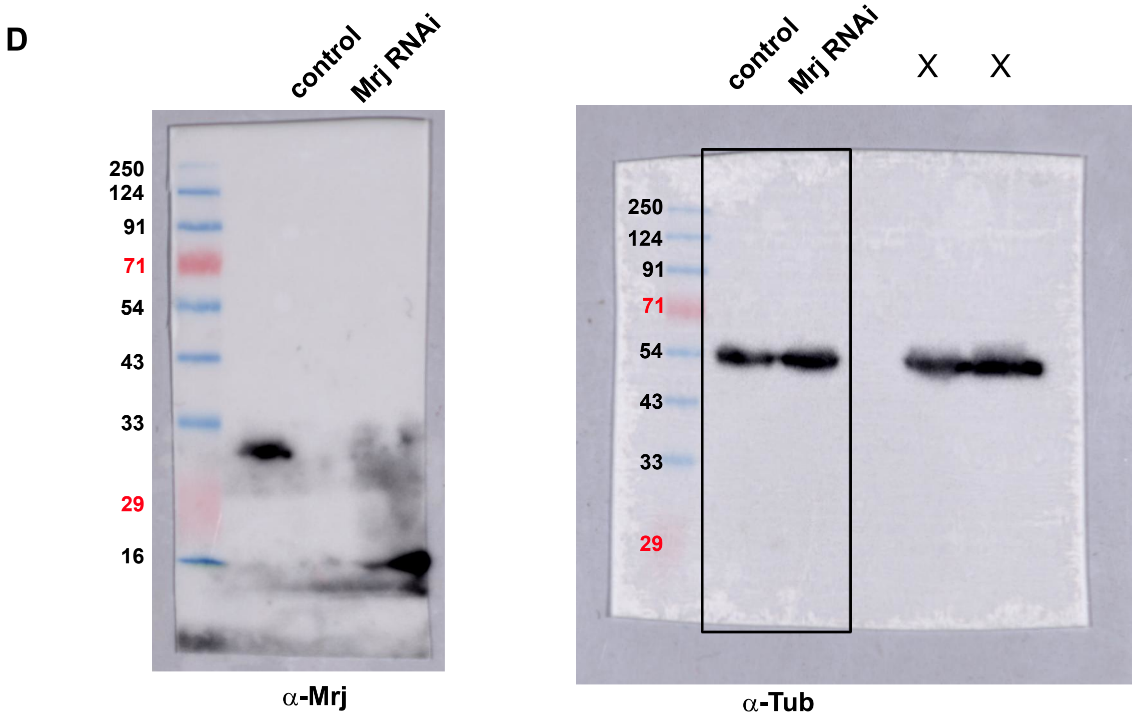

For Tubulin blot 1/3rd of the same lysates as used in the mrj blot were ran on SDS PAGE and processed for western blotting

All blots scanned in GE Imager 600

Figure 7

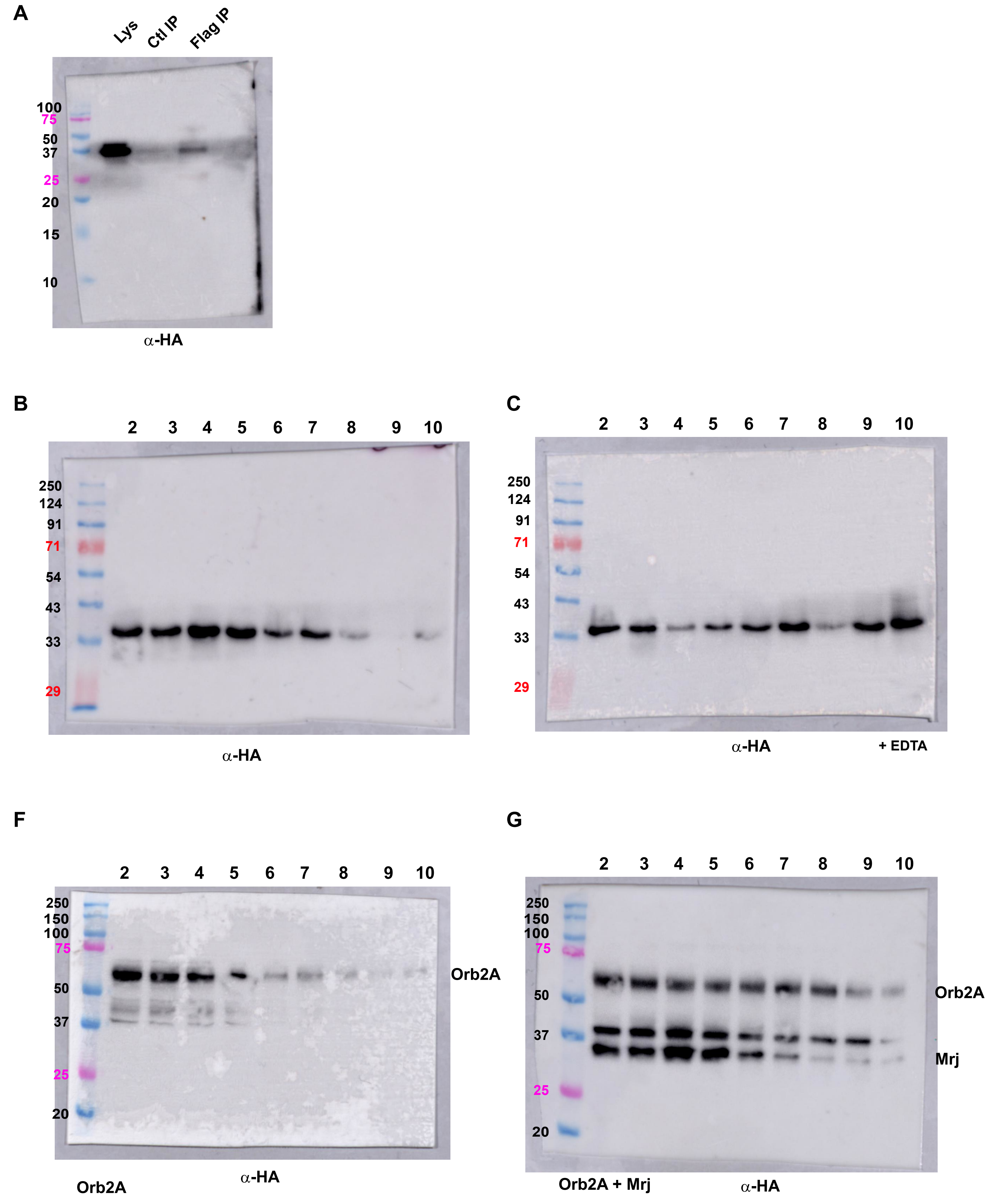

All blots scanned in GE Imager 600

S3 Figure

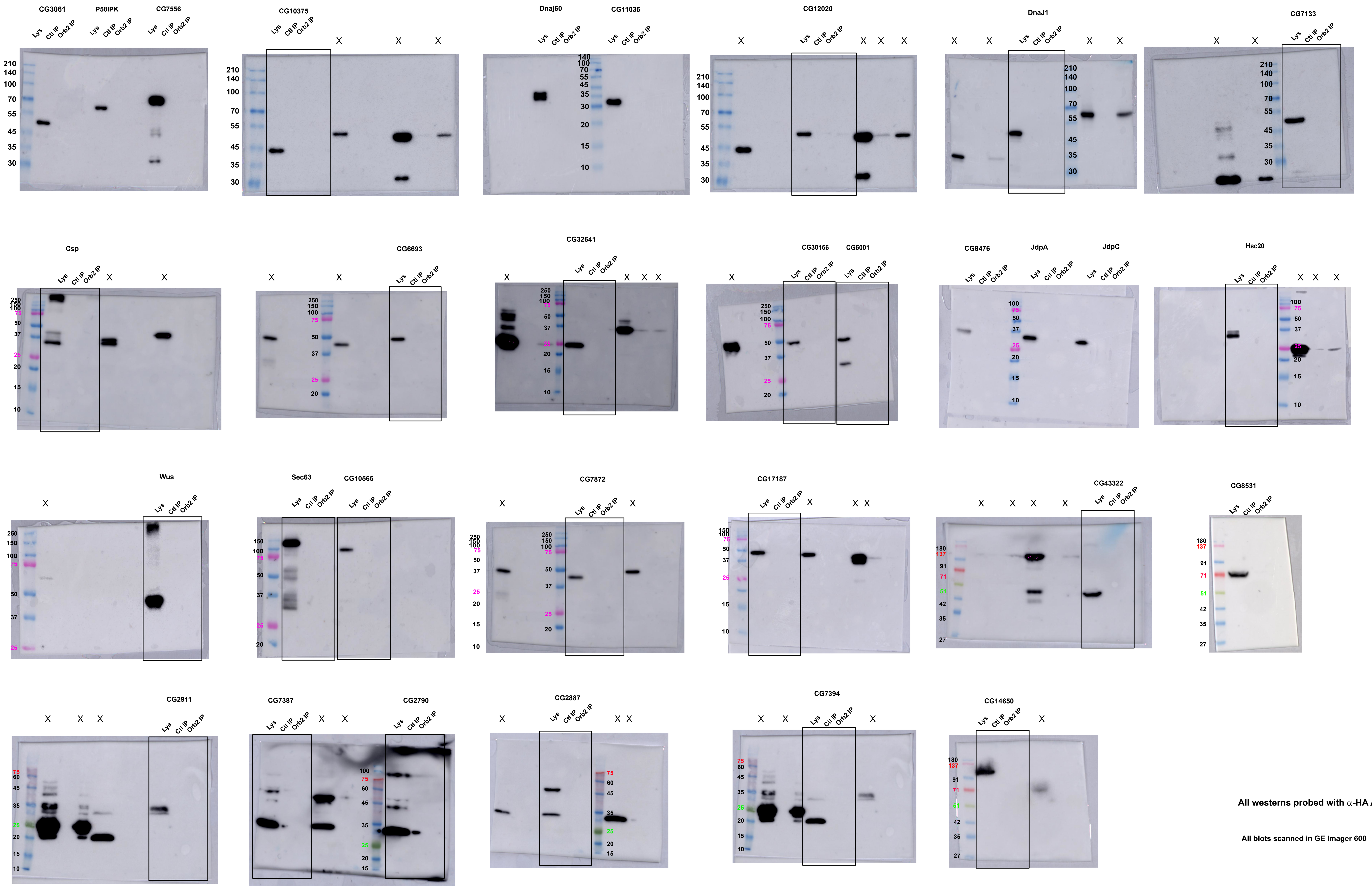

All westerns probed with α-HA Ab

All blots scanned in GE Imager 600

S4 Figure

B

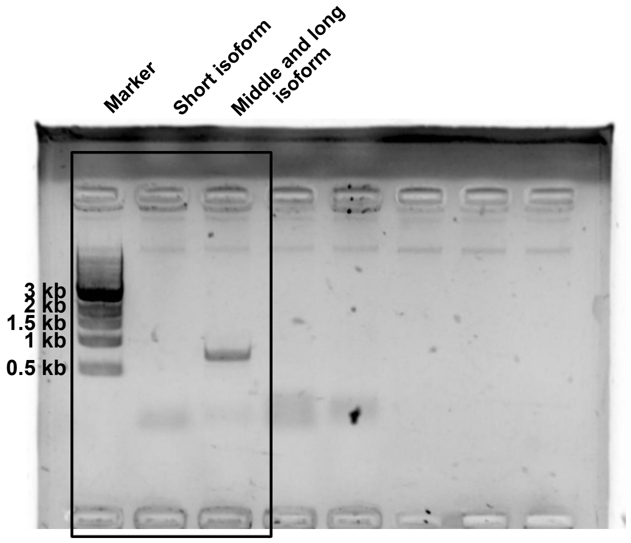

E

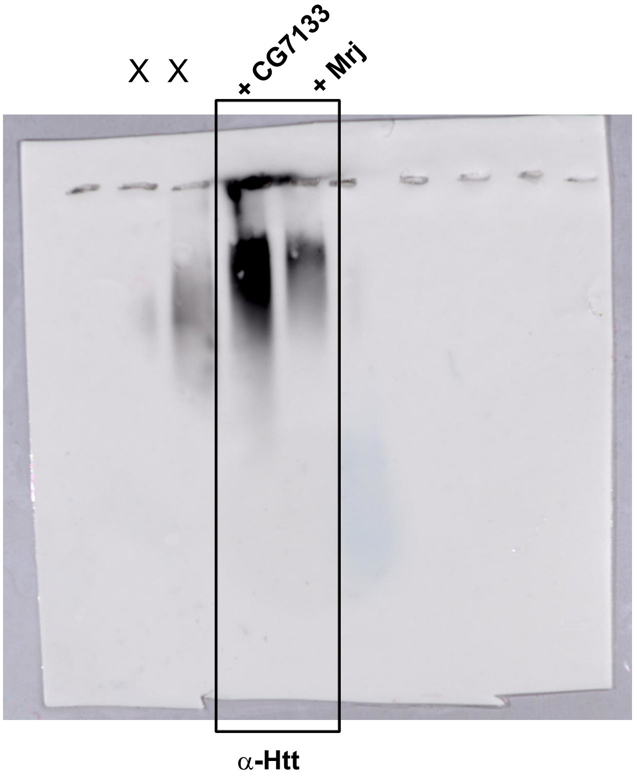

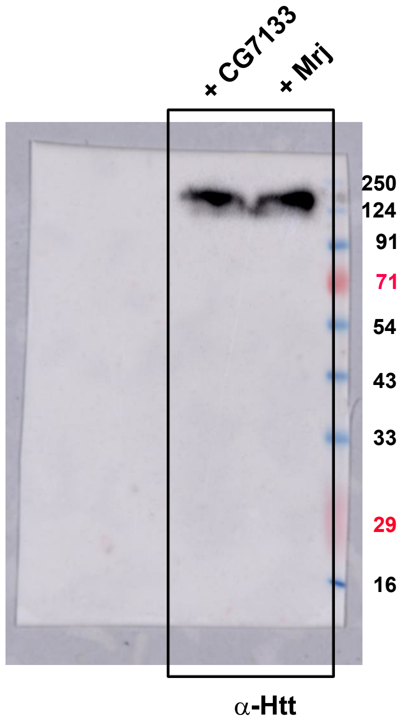

All blots scanned in GE Imager 600

S5 Figure

H

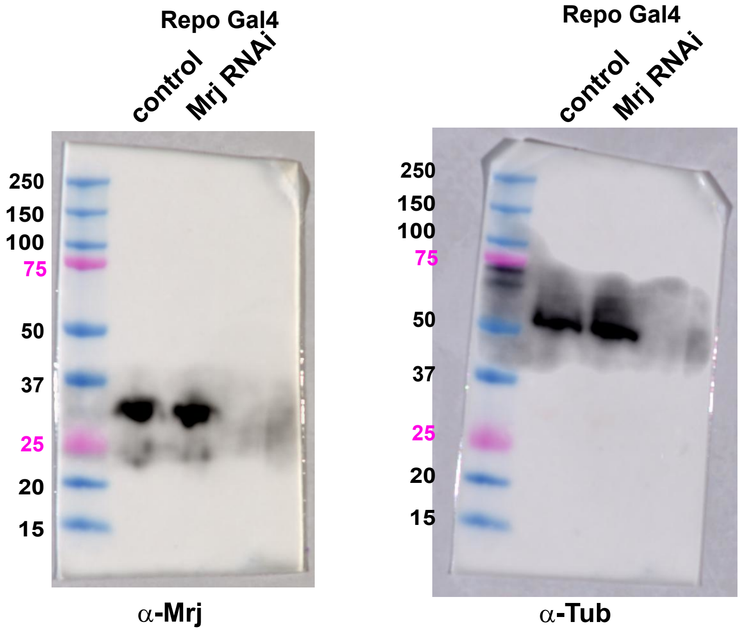

All blots scanned in GE Imager 600

S6 Figure

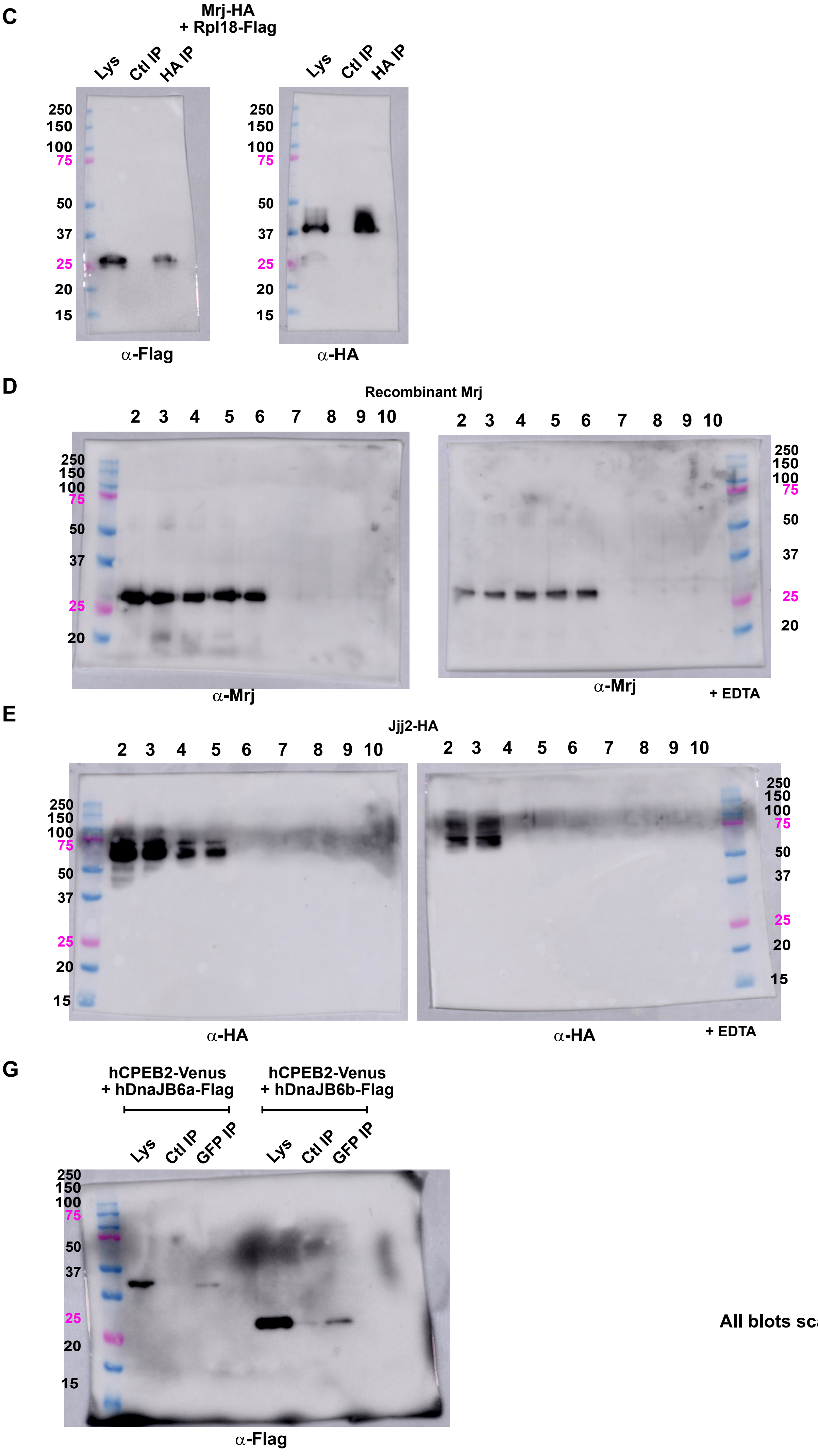

All blots scanned in GE Imager 600

S7 Figure

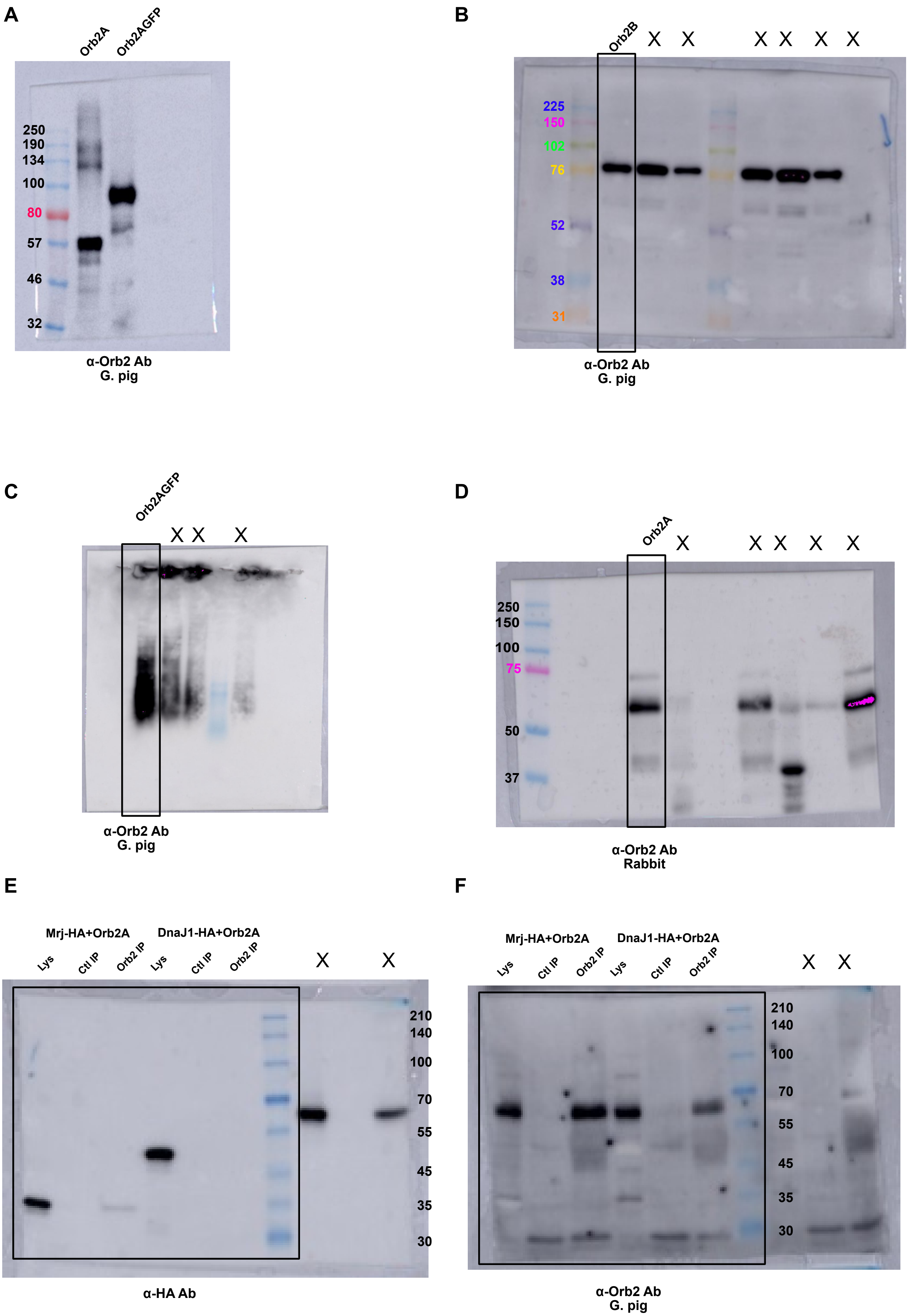

All blots scanned in GE Imager 600
