# Supplementary material for: Mrj is a chaperone of the Hsp40 family that regulates Orb2 oligomerization and long-term memory in Drosophila
Source: PLoS Biol. 2024 Apr 22;22(4):e3002585. doi: 10.1371/journal.pbio.3002585 (PMC11034981; doi:10.1371/journal.pbio.3002585)
Supplement: Attachment — Submitted filename: 131752_1_rebuttal_2628207_rrs6vn.pdf [file pbio.3002585.s011.pdf]

Full Revision

Manuscript number: RC-2022-01752
Corresponding author(s): Amitabha, Majumdar

[Please use this template only if the submitted manuscript should be considered by the affiliate journal as a full revision in response to the points raised by the reviewers.

*If you wish to submit a preliminary revision with a revision plan, please use our "Revision Plan" template. **It is important to use the appropriate template to clearly inform the editors of your intentions.**]*

1. General Statements [optional]

This section is optional. Insert here any general statements you wish to make about the goal of the study or about the reviews.

We would like to thank the reviewers for their extensive review of our manuscript and constructive criticism. We have attempted to address the points raised in the reviewer's comments and have performed additional experiments and have edited the text of the manuscript to explain these points. Please see below, our point-by-point response (in blue) to the reviewer's comments. In the submitted revised manuscript, some figure numbers have changed from the prior reviewed version.

2. Point-by-point description of the revisions

This section is mandatory. Please insert a point-by-point reply describing the revisions that were already carried out and included in the transferred manuscript.

Reviewer #1 (Evidence, reproducibility and clarity (Required)):

In this MS, Mrj - a member of the JDP family of Hsp70 co-chaperones was identified as a regulator of the conversion of Orb2A (the Dm ortholog of CPEB) to its prion-like form.

In drosophila, Mrj deletion does not cause any gross neurodevelopmental defect nor leads to detectable alterations in protein homeostasis. Loss of Mrj, however, does lead to altered Orb2 oligomerization. Consistent with a role of prion-like characteristics of Orb2 in memory consolidation, loss of Mrj results in a deficit in long-term memory.

Aside from the fact that there are some unclarities related to the physicochemical properties of Orb2 and how Mrj affects this precisely, the finding that a chaperone could be important for memory is an interesting observation, albeit not entirely novel.

In addition, there are several minor technical concerns and questions I have that I feel the authors should address, including a major one related to the actual approach used to demonstrate memory deficits upon loss of Mrj.

Reviewer #1 (Significance (Required)):

Figure 1 (plus related Supplemental figures):

• There seem to be two isoforms of Mrj (like what has been found for human DNAJB6). I find it striking to see that only (preferentially?) the shorter isoform interacts with Orb2. For DNAJB6, the long isoform is mainly related to an NLS and the presumed substrate binding is identical for both isoforms. If this is true for Dm-Mrj too, the authors could actually use this to demonstrate the specificity of their IPs where Orb2 is exclusively non-nuclear?

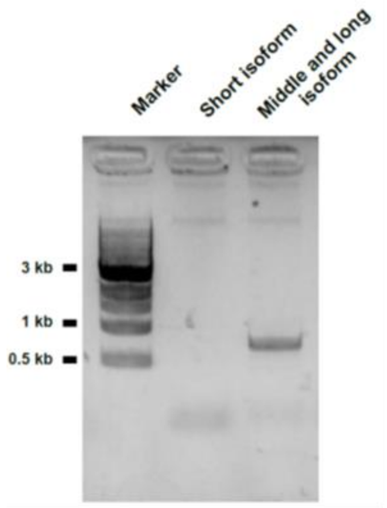

Figure 1: RT-PCR using isoform specific primer sets shows amplification of only the 777 nucleotides long form which corresponds to 259 amino acid isoform of Mrj

According to Flybase, Mrj has 8 predicted isoforms of which four are of 259 amino acids (PA, PB, PC, and PD), 3 are of 346 amino acids (PE, PG, and PH) and one is of 208 amino acids (PF) length (Supplementary data 1). We isolated RNA from fly heads and used this in RT-PCR experiments to check which Mrj isoforms express in the brain. Since both the 346 amino acid (1038 nucleotide long) and 259 amino acids (777 nucleotides long) form, which we refer to as the long and middle isoform, has the same N and C terminal sequences we used the same primer pair for this, but on RT-PCR the only amplicon we got corresponds to the 259 amino acid form. For the 208 amino acids (624 nucleotides long) form we designed a separate forward primer and attempted to amplify this using RT-PCR but were unable to detect this isoform also. This data is now presented in Supplemental Figure 4B. Since the only isoform detected from fly head cDNA corresponded to the 259 amino acid form, we think this is the predominant isoform of Mrj expressing in Drosophila and this is what is in our DnaJ library and what we have used in all our experiments here. This is also the same isoform described in previous papers on Drosophila Mrj (Fayazi et al, 2006; Li et al, 2016b). For this 259 amino acid Mrj isoform, we see its expression in both the nucleus and cytoplasm (Supplemental Figure 4C). As the long 346 AA fragment was undetectable in the brain, it was not feasible to address the reviewer's point of using the long and short forms of Mrj for IP with Orb2. However, we have performed IP of human CPEB2 (hCPEB2) with the long and short isoforms of human DnaJB6 and have detected interaction of hCPEB2 with both the long and short isoforms of DnaJB6 (Supplemental Figure 6E).

• I would be interested to know a bit more about the other 5 JDPs that are interactors with Orb2:

are the human orthologs of those known? It is striking that these other 5 JDPs interact with Orb2 in Dm (in IPs) but have no impact on Sup35 prion behavior. Importantly, this does not imply they may not have impact on the prion-like behavior of other Dm substrates, including Dm-Orb2.

We have performed BlastP analysis of CG4164, CG9828, CG7130, DroJ2, and Tpr2 protein sequences against Human proteins. Based on this we have listed the highest-ranking candidate identified here for each of these genes.

Drosophila Gene	Human gene	Query cover	Percent identity	E value
CG4164	dnaJ homolog subfamily B member 11 isoform 1	98 %	62.96%	2e-150
CG9828	dnaJ homolog subfamily A member 2	92%	39.41%	3e-84
CG7130	dnaJ homolog subfamily B member 4 isoform d	56%	69.44%	2e-30
Tpr2	dnaJ homolog subfamily C member 7 isoform 1	93%	46.22%	6e-139
DroJ2	dnaJ homolog subfamily A member 4 isoform 2	98%	60.60%	2e-169

In the context of the chimeric Sup35-based assay where Orb2A's Prion-like domain (PrD) is coupled with the C-terminal domain of Sup35, the only protein which could convert Orb2A PrD-Sup35 C from its non-prion state to prion state was Mrj. Within the limitations of this heterologous-system-based assay, the other 5 DnaJ domain proteins as well as the Hsp70's were unable to convert the Orb2A PrD from its non-prion to prion-like state. What these other 5 interacting JDP proteins are doing through their interaction with Orb2A and if they are even expressing in the Orb2 relevant neurons will need to be tested separately and will be the subject of our future studies.
.

• The data in panels H, I indeed suggest that Mrj1 alters the (size of) the oligomers. It would be important to know what is the actual physicochemical change that is occurring here. The observed species are insoluble in 0.1 % TX100 but soluble in 0.1% SDS, which suggest they could be gels, but not real amyloids such as formed by the polyQ proteins that require much higher SDS concentrations (~2%) to be solubilized. This is relevant as Mrj1 reduces polyQ amyloidogenesis whereas is here is shown to enhance Orb2A oligomerization/gelidification. In the same context, it is striking to see that without Mrj the amount of Orb2A seems drastically reduced and I wonder whether this might be due to the fact that in the absence of Mrj a part of Orb2A is not recovered/solubilized due to its conversion for a gel to a solid/amyloid state? In other words: Mrj1 may not promote the prion state, but prevents that state to become an irreversible, non-functional amyloid?

On the reviewer's point to address what is the actual physicochemical change occurring here, we will need to develop methods to purify the Orb2 oligomers in significant quantities to examine and distinguish if they are of gel or real amyloid-like nature. Currently, within the limitations of our ongoing work, this has not been possible for us to do and we can attempt to address this in our future work. Cryo-EM derived structure of endogenous Orb2 oligomers purified from a fly head

extract from 3 million fly heads, made in the TritonX-100 and NP-40 containing buffer, the same buffer as what we have used here for the first soluble fraction, showed these oligomers as amyloids (Hervas *et al*, 2020). If the oligomers extracted using 0.1% and 2% SDS are structurally and physicochemically different, within the limitations of our current work, had not been possible to address.

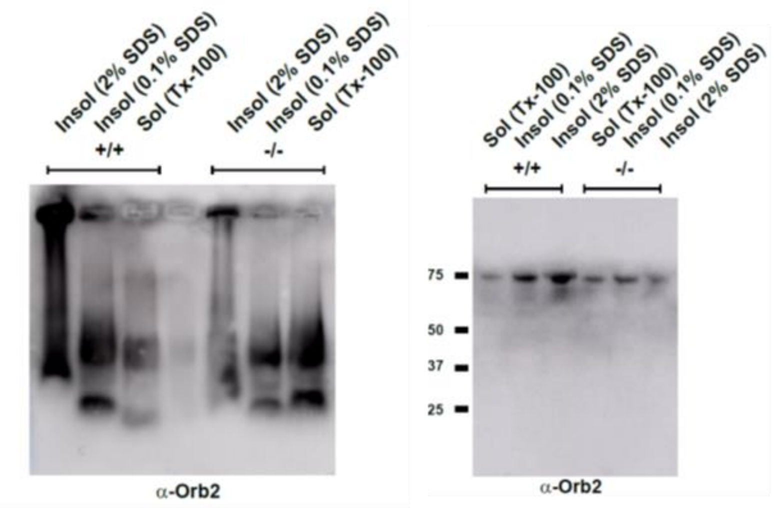

Figure 2:
Left Panel: Probing the SDD-AGE of different fractions from wild type and Mrj knockout flyheads show, increased presence of Orb2 in the TritonX-100 extracted fraction from Mrj knockout in comparison to the wildtype. However, for both the 0.1% and 2% SDS extracted fractions the Orb2 oligomer levels were found to be reduced in the Mrj knockout
Right Panel: Probing the SDS-PAGE of the same extracted fractions with anti-Orb2 antibody shows the similar trend of increased amounts of Orb2B monomers in the TritonX-100 extracted fraction and decreased amounts of Orb2B monomers in the 0.1% and 2% SDS extracted fractions from Mrj knockout in comparison to wild type. For the 2% SDS extracted fraction, 25% of the total eluate was ran to avoid saturation of signal.

The other point raised by the reviewer is if in the absence of Mrj (in the context of Figure 4 of our previously submitted manuscript), a part of Orb2 is not solubilized due to us using a lower 0.1% SDS for extraction. To address this, we attempted to see how much of leftover Orb2 is remaining in the pellet after extraction with 0.1 % SDS. Towards this, according to the reviewers' suggestion, we used a higher 2% SDS containing buffer to resuspend the leftover pellet after 0.1% SDS extraction, and post solubilisation ran all the fractions in SDD-AGE. We did this experiment with both wild-type and Mrj knockout fly heads. Under these different extractions, we first observed while there is more Orb2 in the soluble fraction (Triton X-100 extracted) of Mrj knockout, this amount is reduced in both the 0.1% SDS solubilized and 2% SDS solubilized fractions. So, even though there is leftover Orb2 after 0.1% SDS extraction, which can be extracted using 2% SDS, this amount is reduced in Mrj knockout. The other observation here is the Orb2 extracted using 2% SDS shows a longer smear in comparison to the 0.1% SDS extracted form suggesting a possibility of more and higher-sized oligomers present in this fraction. Since we do not have the exact physicochemical characterization of these oligomers detected with 0.1% and 2% SDS-containing buffer, we are not differentiating them by using the terms gels and real amyloids, but refer to them as 0.1% SDS soluble Orb2 oligomers and 2% SDS soluble Orb2 oligomers. Overall, our observations here suggest in absence of Mrj, both of these kinds of Orb2 oligomers are decreased and so Mrj is most likely promoting the formation of Orb2 oligomers. It is possible that the 0.1% SDS soluble Orb2 oligomers gradually accumulate and undergo a further transition to the 2% SDS soluble Orb2 oligomers, so if in absence of Mrj, the formation of the 0.1% SDS

soluble Orb2 oligomers is decreased, the next step of formation of 2% SDS soluble Orb2 oligomers also be decreased. This data is now presented in Figures 5H, I, and J).
On the other possibility raised by the reviewer that Mrj can prevent the oligomeric state of Orb2 to become an irreversible non-functional amyloid, we think it is still possible for Mrj to do this but this could not be tested under the present conditions.

• It may be good for clarity to refer to the human Mrj as DNAJB6 according to the HUGO nomenclature. Also, the first evidence for its oligomerization was by Hageman et al 2010.
We have now changed mentions of human Mrj to DNAJB6. We apologize for missing the Hageman et al 2010 reference and have now cited this reference in the context of Mrj oligomerization.

• It is striking to see that Mrj co-Ips with Hsp70AA, Hsp70-4 but not Hsp70Cb. The fact that interactions were detected without using crosslinking is also striking given the reported transient nature of J-domain-Hsp70 interactions Together, this may even suggest that Mrj-1 is recognized as a Hsp70 substrate (for Hsp70AA, Hsp70-4 but not Hsp70Cb) rather than as a co-chaperone. In fact, a variant of Mrj-1 with a mutation in the HPD motif should be used to exclude this option.
In IP experiments we notice Mrj interacts with Hsp70Aa and Hsc70-4 but not with Hsc70-1 and Hsc70Cb. In our previously submitted manuscript, we realized we made a typo on the figure, where we referred to Hsp70Aa as Hsc70Aa. We have corrected this in the current revised manuscript. On the crosslinking point raised by the reviewer, we reviewed the published literature for studies of immunoprecipitation experiments which showed an interaction between DnaJB6 and Hsp70. We noted while one of the papers (Kakkar *et al*, 2016) report the use of a crosslinker in the experiment which showed an interaction between GFP-Hsp70 and V5-DnaJB6, in another two papers the interaction between endogenous Mrj and endogenous Hsp/c70 (Izawa *et al*, 2000) and Flag-Hsp70 and GFP-DnaJB6 (Bengoechea *et al*, 2020) could be detected without using any crosslinker. Our observations of detecting the interaction of Mrj with Hsp70Aa and Hsc70-4 in the absence of a crosslinker are thus similar to the observations reported by (Izawa *et al*, 2000; Bengoechea *et al*, 2020).
On the point of if Mrj is a substrate for Hsp70aa and Hsc70-4 and not a co-chaperone, we feel in the context of this manuscript, since we are focussing on the role of Mrj in the regulation of oligomerization of Orb2 and memory, the experiment with HPD motif mutant is probably not necessary here. However, if the reviewers suggest this experiment to be essential, we can attempt this experiment by making this HPD motif mutant.

• The rest of these data reconfirm nicely that Mrj/DNAJB6 can suppress polyQ-Htt aggregation. Yet note that in this case the oligomers that enter the agarose gel are smaller, not bigger. This argues that Mrj is not an enhancer of oligomerization, but rather an inhibitor of the conversion of oligomers to a more amyloid like state.

Figure 2 and Supplemental Figure 4 discuss the effect of Mrj on Htt aggregation. We have used 2 different Htt constructs here. For Figure 2I, we used only Exon1 of Htt with the poly Q repeats. Here in SDD-AGE, for the control lane, we see the Htt oligomers as a smear for the control. For

Mrj, we see only a small band at the bottom which can be interpreted most likely as either a monomer or as small oligomers since we do not observe any smear here. However, for the 588 amino acid fragment of HttQ138 in the SDD-AGE we do not see a difference in the length of the smear but in the intensity of the smear of the Htt oligomers (Supplemental Figure 4E). Based on this we are suggesting in presence of Mrj, there are lesser Htt oligomers. On the point of Mrj is not an enhancer of oligomerization, but rather an inhibitor of the conversion of oligomers to a more amyloid-like state, our experiments with the Mrj knockout show reduced Orb2 oligomers (both for 0.1% and 2% SDS soluble forms), suggesting Mrj plays a role in the conversion of Orb2 to the oligomeric state. If Mrj inhibits the conversion of oligomers to a more amyloid-like state, this is possible but we couldn't test this hypothesis here. However, for Htt amyloid aggregates, previous works done by other labs with DnaJB6 as well as our experiments with Mrj suggest this as a likely possibility.

Figure 3:

• The finding that knockout of DNAJB6 in mice is embryonic lethal is related to a problem with placental development and not embryonic development (Hunter et al, 1999; Watson et al, 2007, 2009, 2011) as well recognized by the authors. Therefore, the finding that deletion of Dm-Mrj has no developmental phenotype in Drosophila may not be that surprising.

We agree with the reviewer's point that DNAJB6 mutant mice have a problem with placental development. However, one of the papers cited here (Watson *et al*, 2009) suggests DNAJB6 also plays a crucial role in the development of the embryo independent of the placenta development defect. The mammalian DNAJB6 mutant embryos generated using the tetraploid complementation method show severe neural defects including exencephaly, defect in neural tube closure, reduced neural tube size, and thinner neuroepithelium. Due to these features seen in the mice knockout, and the lack of such developmental defects in the Drosophila knockout, we interpreted our findings in Drosophila as significantly different from the mammals.

• It is a bit more surprising that Mrj knockout flies showed no aggregation phenotype or muscle phenotype, especially knowing that DNAJB6 mutations are linked to human diseases associated with aggregation (again well recognized by the authors). However, most of these diseases are late-onset and the phenotype may require stress to be revealed. So, while important to this MS in terms of not being a confounder for the memory test, I would like to ask the authors to add a note of caution that their data do not exclude that loss of Mrj activity still may cause a protein aggregation-related disease phenotype. Yet, I also do think that for the main message of this MS, it is not required to further test this experimentally.

We agree with the reviewer and have added this suggestion in the discussion that the loss of Mrj may still result in a protein aggregation-related disease phenotype, probably under a sensitized condition of certain stresses which is not tested in this manuscript.

Figure 4:

• IPs were done with Orb2A as bite and clearly pulled down substantial amounts of GFP-tagged Mrj. For interactions with Orb2B, a V5-tagged Mrj was use and only a minor fraction was pulled down. Why were two different Mrj constructs used for Arb2A and Orb2b?

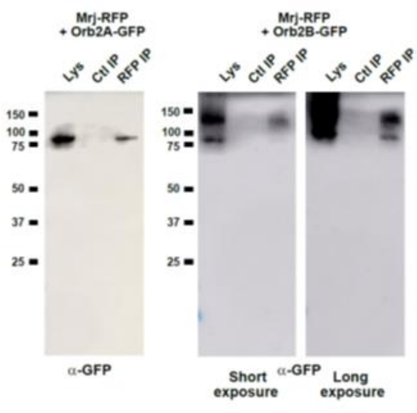

Figure 3:
Left Panel: Immunoprecipitated Mrj-RFP pulls down Orb2A-GFP
Right Panel: Immunoprecipitated Mrj-RFP pulls down Orb2B-GFP .

In the previously submitted manuscript, we have used HA-tagged Mrj (not V5) for checking the interaction with full-length Orb2B tagged with GFP. This was done using the same Mrj-HA construct as that used in the initial Orb2A immunoprecipitation experiment. Since this has raised some concerns as in the IPs to check for interaction between truncated Orb2A constructs (Orb2A325-GFP and Orb2AΔ162-GFP) and Mrj (Mrj-RFP), we used a different GFP and RFP tag combination. To address this, we have now added the same tag combinations for the IPs (Mrj-RFP with Orb2A-GFP and Orb2B-GFP). In these immunoprecipitation experiments where Mrj-RFP was pulled down using RFP Trap beads, we were able to detect positive interaction with GFP-tagged Orb2A and Orb2B. This data is now added in Figures 4F and 4I. We also have added the IP data for interaction between Mrj-HA and untagged Orb2B in Figure 4K, similar to the combination of the initial experiment between Mrj-HA and untagged Orb2A.

• In addition, I think it would be important what one would see when pulling on Mrj1, especially under non-denaturing conditions and what is the status of the Orb2 that is than found to be associated with Mrj (monomeric, oligomeric and what size).

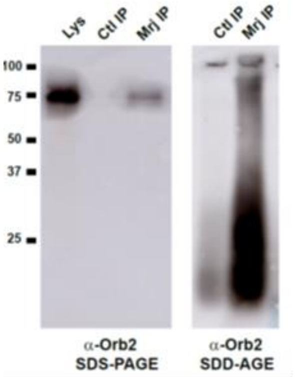

Figure 4:
Left Panel: Probing the SDS-PAGE of Mrj immunoprecipitate with anti-Orb2 antibody detects the presence of monomeric Orb2B
Right Panel: Probing the SDD-AGE of Mrj immunoprecipitate with anti Orb2 antibody shows the presence of Orb2 oligomers

We have now performed IP from wild-type fly heads using anti Mrj antibody and ran the immunoprecipitate in SDS-PAGE and SDD-AGE followed by immunoblotting them with anti-Orb2 antibody. Our experiments suggest that immunoprecipitating endogenous Mrj brings down both the monomeric and oligomeric forms of Orb2. This data is now added in Figures 4L, M, and N.

• This also relates to my remark at figure 1 and the subsequent fractionation experiments they show here in which there is a slight (not very convincing) increase in the ratio of TX100-soluble and insoluble (0.1% SDS soluble) material. My question would be if there is a remaining fraction of 0.1% insoluble (2% soluble) Orb2 and how Mrj affects that? As stated before, this is (only) mechanistically relevant to understanding why there is less oligomers of Orb2 in terms of Mrj either promoting it or by preventing it to transfer from a gel to a solid state. The link to the memory data remains intriguing, irrespective of what is going on (but also on those data I do have several comments: see below).
We have addressed this in response to the reviewer's comments on Figure 1. We find in both wild type and Mrj knockout fly heads, there are Orb2 oligomers that can be detected using 0.1% SDS extraction and with further extraction with 2% SDS. The 2% SDS soluble Orb2 oligomers were

previously insoluble during 0.1% SDS-based extraction. However, the amounts of both of these oligomers are reduced in Mrj knockout fly heads. Since we do not have the physicochemical characterization of both of these kinds of oligomers, we are not using the terms gel or solid state here but referring to these oligomers as 0.1% SDS soluble Orb2 oligomers and 2% SDS soluble Orb2 oligomers. We speculate that the 0.1% SDS soluble Orb2 oligomers over time transition to the 2% SDS soluble Orb2 oligomers. As in the absence of Mrj in the knockout flies, both the 0.1% SDS soluble and 2% SDS soluble Orb2 oligomers are decreased, this suggests Mrj is promoting the formation of Orb2 oligomers. On the reviewer's point, if Mrj can prevent the transition from 0.1% SDS soluble to 2% SDS soluble Orb2 oligomers, we think it is possible for Mrj to both promote oligomerization of Orb2 as well as prevent it from forming bigger non-functional oligomers, but the second point is not tested here. The relevant data is now presented in Figures 5H, I, and J.

• I also find the sentence that "Mrj is probably regulating the oligomerization of endogenous Orb2 in the brain" somewhat an overstatement. I would rather prefer to say that the data show that Mrj1 affects the oligomeric behavior/status of Orb2.

Based on the reviewer's suggestion we have now changed the sentence to Mrj is probably regulating the oligomeric status of Orb2

Figure 5:

• To my knowledge, the Elav driver regulates expression in all neurons, but not in glial cells that comprise a significant part of the fly heads/brain. The complete absence of Mrj in the fly-heads when using this driver is therefore somewhat surprising. Or do we need to conclude from this that glial cells normally already lack Mrj expression?

On driving Mrj RNAi with Elav Gal4, we did not detect any Mrj in the western. We attempted to address the glial contribution towards Mrj's expression we used a Glia-specific driver Repo Gal4 line to drive the control and Mrj RNAi line and performed a western blot using fly head lysate with anti-Mrj antibody. In this experiment, we did not observe any difference in Mrj levels between the two sets. As the Mrj antibody raised by us works in western blots but not in immunostainings, we could not do a colocalization analysis with a glial marker. However, we used the Mrj knockout Gal4 line to drive NLS-GFP and performed immunostainings of these flies with a glial marker anti-Repo antibody. Here we see two kinds of cells in the brain, one which has GFP but no Repo and the other where both are present together. This suggests that while Glial cells have Mrj but probably the majority of Mrj in the brain comes from the neurons. We also found a reference where it was shown that Elav protein as well as Elav Gal4 at earlier stages of development expresses in neuroblasts and in all Glia (Berger et al, 2007). So, another possibility is when we are driving Mrj RNAi using Elav Gal4, this knocks down Mrj in both the neurons as well as in the glia. This coupled with the catalytic nature of RNAi probably creates an effective knockdown of Mrj as seen in the western blot. This data is now added in Supplementary Figures 5G and H.

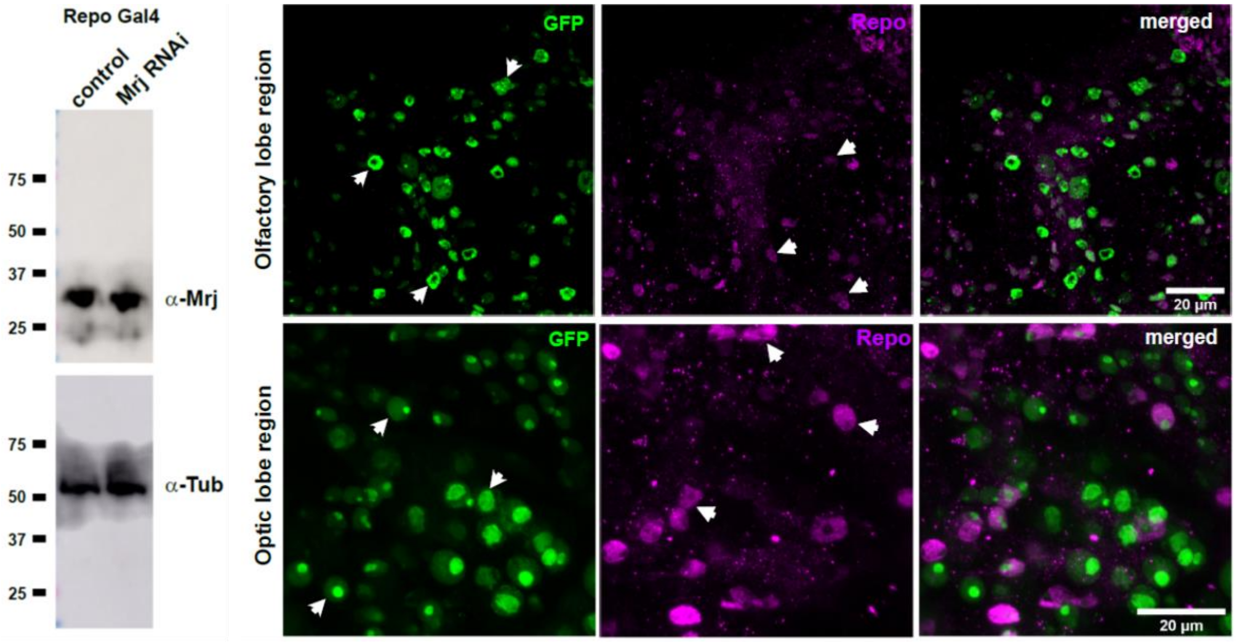

Figure 5:
Left panel: Driving the Mrj RNAi line in Glial cells using Repo Gal4 shows no difference in Mrj expression in comparison to the control animals as seen in the western blot with anti-Mrj antibody. Anti-α-Tubulin antibody was used as the loading control
Right Panel: Representative images from olfactory and optic lobe regions of Mrj KO gal4 driving NLS-GFP brains immunostained with glia specific anti-Repo antibody. Arrows in the GFP panel depicts cells with only GFP and no Repo expression. Arrows in the Repo panel depicts cells with both GFP and Repo expression suggesting the likely expression of Mrj in glial cells.

• Why not use these lines also for the memory test for confirmation? I understand the concerns of putative confounding effects of a full knockdown (which were however not reported), but now data rely only on the mushroom body-specific knockdown for the 201Y Gal4 line, for which the knockdown efficiency is not provided. But even more so, here a temperature shift (22oC-30oC) was required to activate the expression of the siRNA. For the effects of this shift alone no controls were provided. The functional memory data are nice and consistent with the hypothesis, but can it be excluded that the temperature shift (rather than the Mrj) knockdown has caused the memory defects? I think it is crucial to include the proper controls or use a different knockdown approach that does not require temperature shifts or even use the knockout flies.

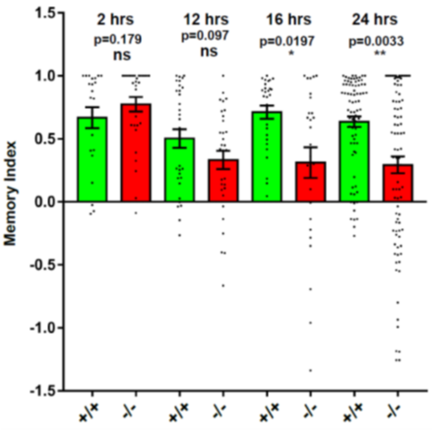

Figure 6:
Mrj knockout flies shows significant memory deficit in comparison to the wild type flies from 16 hours onwards.

We have now performed the memory experiments with Mrj knockout flies. Our experiments show at 16 and 24-hour time points Mrj knockout flies have significantly reduced memory in comparison to the control wildtype. This data is now added in Figure 6B.

Figure 6:

The finding of a co-IP between Rpl18 and Mrj (one-directional only) by no means suffices to conclude that Mrj may interact with nascent Orb2 chains here (which would be the relevant finding here). The fact that Mrj is a self-oligomerising protein (also in vitro, so irrespective of ribosomal associations!), and hence is found in all fractions in a sucrose gradient, also is not a very strong case for its specific interaction with polysomes. The finding that there is just more self-oligomerizing Orb2A co-sedimenting with polysomes in sucrose gradients neither is evidence for a direct effect of Mrj enhancing association of Orb2A with the translating ribosomes even though it would fit the hypothesis. So all in all, I think the data in this figure and non-conclusive and the related conclusions should be deleted.

We have now performed the reverse co-IP between Rpl18-Flag and Mrj-HA using an anti-HA antibody and could detect an interaction between the two. This data is now added in Supplementary Figure 6A.

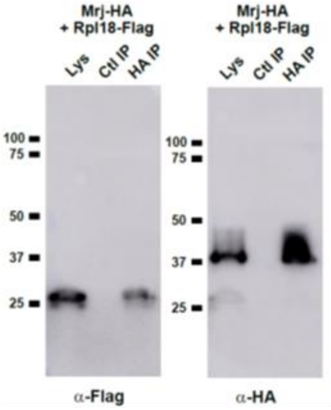

Figure 7:
Immunoprecipitated Mrj-HA pulls down Rpl18-Flag which is incorporated in ribosomes, suggesting Mrj is associated with ribosomes. The right panel shows the same blot probed with anti-HA antibody and confirming the presence of Mrj-HA in the immunoprecipitate

To address if Mrj is a self-oligomerizing protein that can migrate to heavier polysome fractions due to its size, we have loaded recombinant Mrj on an identical sucrose gradient as we use for polysome analysis. Post-ultra-centrifugation we fractionated the gradients and checked if Mrj can be detected in the fraction numbers where polysomes are present. In this experiment, we could not detect recombinant Mrj in the heavier polysome fractions (data presented in Supplementary Figure 6B). Overall, our observations of Mrj-Rpl18 IPs, the presence of cellularly expressed Mrj in polysome fractions, and the absence of recombinant Mrj from these fractions, suggest a likelihood of Mrj's association with the translating ribosomes.

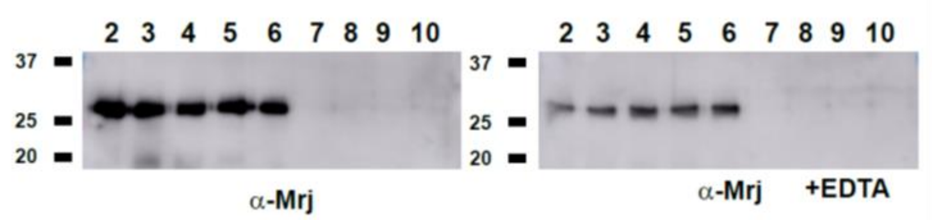

Figure 8:
Loading recombinant Mrj on an identical polysome gradient followed by centrifugation and fractionation and probing these fractions with anti-Mrj antibody shows their absence from the heavier polysome fractions. Also, on EDTA treatment the recombinant Mrj does not move to the heavier fractions, unlike our observations with cellularly expressed Mrj

On the reviewer's point of us concluding Mrj may interact with nascent Orb2 chains, we have not mentioned this possibility in the manuscript as we don't have any evidence to suggest this. We

have also added a sentence: This indicates that in presence of Mrj, the association of Orb2A with the translating ribosomes is enhanced, however, if this is a consequence of increased Orb2A oligomers due to Mrj or caused by interaction between polysome-associated Orb2A and Mrj will need to be tested in future.
Based on these above-mentioned points, we hope we can keep the data and conclusions of this section.

Overall, provided that proper controls/additional data can be provided for the key experiments of memory consolidation, I find this an intriguing study that would point towards a role of a molecular chaperone in controlling memory functions via regulating the oligomeric status of a prion-like protein and that is worthwhile publishing in a good journal.

However, in terms of mechanistical interpretations, several points have to be reconsidered (see remarks in figure 1,4); this pertains especially to what is discussed on page 13. In addition, I'd like the authors to put their data into the perspective of the findings that in differentiated neurons DNAJB6 levels actually decline, not incline (Thiruvalluvan et al, 2020), which would be counterintuitive if these proteins are playing a role as suggested here in memory consolidation.

We have addressed the comments on Figures 1 and 4 earlier. We have also added new memory experiment data with the Mrj knockout in Figure 6.
We have attempted to put the observations with Drosophila Mrj in perspective to observations in Thiruvalluvan et al, on human DnaJB6 in the discussions as follows:
Can our observation in Drosophila also be relevant for higher mammals? We tested this with human DnaJB6 and CPEB2. In mice CPEB2 knockout exhibited impaired hippocampus-dependent memory (Lu *et al*, 2017), so like Drosophila Orb2, its mammalian homolog CPEB2 is also a regulator of long-term memory. In immunoprecipitation assay we could detect an interaction between human CPEB2 and human DnaJB6, suggesting the feasibility for DnaJB6 to play a similar role to Drosophila Mrj in mammals. However, as the human DnaJB6 level was observed to undergo a reduction in transitioning from ES cells to neurons, (Thiruvalluvan *et al*, 2020) how this can be reconciled with its possible role in the regulation of memory. We speculate, such a reduction if is happening in the brain will occur in a highly regulatable manner to allow precise control over CPEB2 oligomerization only in specific neurons where it is needed and the reduced levels of DnaJB6 are probably sufficient to aid CPEB oligomerization Alternatively, there may be additional chaperones that may function in a stage-specific manner and be able to compensate for the decline in levels of DNAJB6.

Reviewer #2 (Evidence, reproducibility and clarity (Required)):

Summary:
The manuscript describes the role of the Hsp40 family protein Mrj in the prion-like oligomerization of Orb2. The authors demonstrate that Mrj promotes the oligomerization of Orb2, while a loss in Mrj diminishes the extent of Orb2 oligomerization. They observe that while Mrj is not an essential gene, a loss in Mrj causes deficiencies in the consolidation of long-term memory. Further, they

demonstrate that Mrj associates with polysomes and increases the association of Orb2 with polysomes.

Major comments: None

Minor comments:

1. In the section describing the chaperone properties of Mrj in clearing Htt aggregates (Fig 2), the legend describes that "Mrj-HA constructs are more efficient in decreasing Htt aggregation compared to Mrj-RFP". It would be helpful to add Mrj-RFP to the quantification in Fig 2G to know exactly the difference in efficiency. Is there an explanation for why the 2 constructs behave differently?

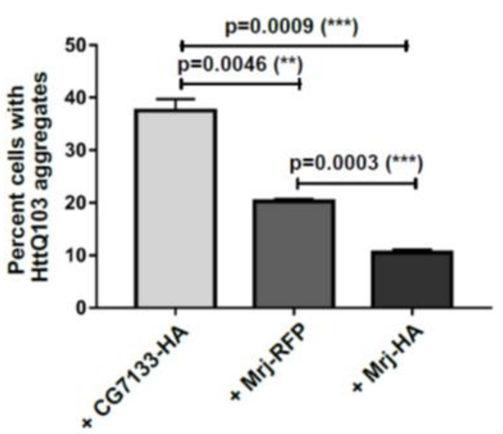

Figure 9:
Quantitation of the percentage of HttQ103-exon1GFP expressing cells with aggregates in presence of CG7133-HA, Mrj-RFP and Mrj-HA suggests a significant decrease of Htt aggregates in presence of both Mrj-RFP and Mrj-HA. The Mrj-HA construct is more efficient in decreasing Htt aggregation compared to Mrj-RFP.

We have added the quantitation of Htt aggregates in presence of Mrj-RFP in the revised version (Data presented in Figure 2G). While the efficiency of Mrj-RFP to decrease Htt aggregates is significantly less in comparison to Mrj-HA, it is still significantly better in comparison to the control CG7133-HA construct. It is possible, due to the tagging of Mrj with a larger tag (RFP), this reduces its ability to decrease the Htt aggregates in comparison to the construct where Mrj is tagged with a much smaller HA tag.

2. Figs A, B, C, G need to have quantification of the percentage of colocalization with details about the number of cells quantified for each experiment.

We have now added the intensity profile images and colocalization quantitation (Pearson's coefficient) in Supplemental Figures 5A and B. This quantitation is done from multiple ROI's taken from 4-6 cells.

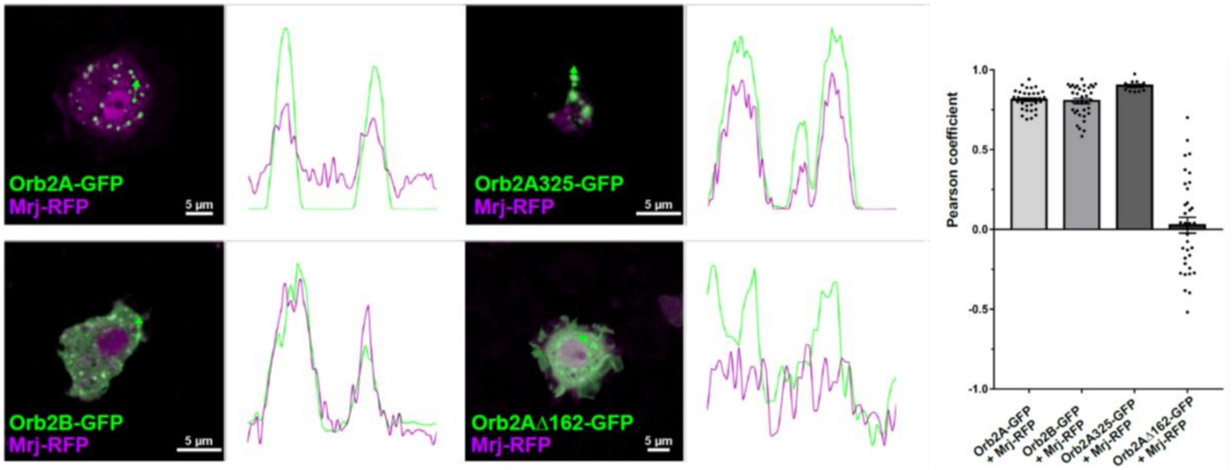

Figure 10:
Left panel: Intensity profile plots from lines drawn over punctae in the cells show colocalization between Orb2A-GFP, Orb2A325-GFP and Orb2B-GFP with Mrj-RFP. The Δ162Orb2A-GFP construct do not show colocalization with Mrj-RFP
Right panel: Colocalization quantitation using Pearson's coefficient shows significant colocalization of Mrj-RFP with Orb2AGFP (n=34 ROI's from 4 cells), Orb2B-GFP (n=36 ROI's from 6 cells), Orb2A325-GFP (n=14 ROI's from 5 cells) but not with Orb2AΔ162-GFP(n=35 ROI's from 5 cells).

3. In Fig 6 B, C, F, G it would be helpful to label the 40S, 60S and 80S peaks in the A 254 trace.

We have now labeled the 80S, and polysome peaks in Figures 7B, C, F and G. We could not separate the 40S and 60S peaks in the A254 trace.

4. It's interesting that Mrj has opposing functions with regard to aggregation when comparing huntingtin with Orb2. From the literature presented in the discussion, it appears as though chaperones including Mrj have an anti-aggregation role for prions. It would be helpful to have more discussion around why, in the case of Orb2, this is different. The discussion states that "The only Hsp40 chaperone which was found similar to Mrj in increasing Orb2's oligomerization is the yeast Jjj2 protein" - this point needs elaboration, as well as a reference.

In the discussions section we have now added the following speculations on this:

One question here is why Mrj behaves differently with Orb2 in comparison to other amyloids. Orb2 differs from other pathogenic amyloids in its extremely transient existence in the toxic intermediate form (Hervás *et al*, 2016). For the pathogenic amyloids, since they exist in the toxic intermediate form for longer, Mrj probably gets more time to act and prevent or delay them from forming larger aggregates. For Orb2, Mrj may help to quickly transition it from the toxic intermediate state, thereby helping this state to be transient instead of longer. An alternate possibility is post-transition from the toxic intermediate state, Mrj stabilizes these Orb2 oligomers and prevents them from forming larger aggregates. This can be through Mrj interacting with Orb2 oligomers and blocking its surface thereby preventing more Orb2 from assembling over it. Another difference between the Orb2 oligomeric amyloids and the pathogenic amyloids is in the nature of their

amyloid core. For the pathogenic amyloids, this core is hydrophobic and devoid of any water molecules, however, for Orb2, the core is hydrophilic. This raises another possibility that if the Orb2 oligomers go beyond a certain critical size, Mrj can destabilize these larger Orb2 aggregates by targeting its hydrophilic core.

On the Jjj2 point raised by the reviewer, we have added the (Li *et al*, 2016a) reference now and elaborated as:

The only Hsp40 chaperone which was found similar to Mrj in increasing Orb2's oligomerization is the yeast Jjj2 protein. In Jjj2 knockout yeast strain, Orb2A mainly exists in the non-prion state, whereas on Jjj2 overexpression the non-prion state could be converted to a prion-like state. In S2 cells coexpression of Jjj2 with Orb2A lead to an increase in Orb2 oligomerization (Li *et al*, 2016a). However, Jjj2 differs from Mrj, as when it is expressed in S2 cells, we do not detect it to be present in the polysome fractions.

The Jjj2 polysome data is now presented in Supplementary Figure 6C.

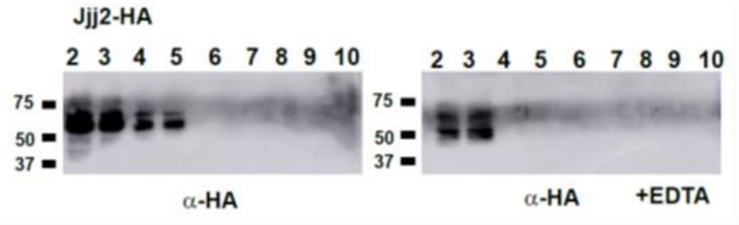

Figure 11:
Polysome fractionation of Jjj2-HA expressing S2 cells in absence and presence of EDTA followed by detection of Jjj2 using western blots shows its absence in the heavier polysome fractions.

Reviewer #2 (Significance (Required)):

General assessment:

Overall, the work is clearly described and the manuscript is very well-written. The motivation behind the study and its importance are well-explained. I only have minor comments and suggestions to improve the clarity of the work.
The study newly describes the interaction between the chaperone Mrj and the translation regulator Orb2. The experiments that the screen for proteins that interact with Orb2 and promote its oligomerization are very thorough. The experiments that delve into the role of Mrj in protein synthesis are a good start, and need to be explored further, but that is beyond the scope of this study.

Advance:
The study describes a new interaction between the chaperone Mrj and the translation regulator Orb2. The study is helpful in expanding our knowledge of prion regulators as well factors that affect memory acquisition and consolidation.

Audience:
This paper will be of most interest to basic researchers.

My expertise is in Drosophila genetics and neuronal injury.

Reviewer #3 (Evidence, reproducibility and clarity (Required)):

The manuscript submitted by Desai et al. identifies a chaperone of the Hsp40 family (Mrj) that binds Orb2 and modulates its oligomerization, which is critical for Orb2 function in learning and memory in Drosophila. Orb2 are proteins with prion-like properties whose oligomerization is critical for their function in the storage of memories. The main contribution of the article is the screen of Hsp40 and Hsp70-family proteins that bind Orb2. The authors show IP results for all the candidates tested, including those that bind Fig. 1) and those that don't (Supp Fig 3). There is also a figure devoted to examining the interaction of Mrj with polyglutamine models (Htt). They also generate a KO mutant that is viable and shows no gross defects or protein aggregation. Lastly, they show that the silencing of Mrj in the mushroom body gamma neurons results in weaker memories in a courtship paradigm. Although the data is consistent and generally supportive of the hypothesis, key details are missing in several areas, including controls. Additionally, the interpretation of some results leaves room for debate. Overall, this is an ambitious article that needs additional work before publication.

Specific comments:

1. General concern over the interpretation of IP experiments and colocalization. These experiments don't necessarily reflect direct interactions. They are consistent with direct interaction but not the only explanation for a positive IP or colocalization.

This paper is centered on the interaction between Orb2A and Mrj, which we have detected using immunoprecipitation. The reviewer's concern here is, this experiment is not able to distinguish if this can be a direct protein-protein interaction or if the two proteins are part of a complex.

To address this concern we have used purified recombinant protein-based pulldowns. Our experiments with purified protein pulldowns (GST tagged Mrj from E.coli with Orb2A from E.coli or Orb2A-GFP from Sf9 cells) suggest Orb2A and Mrj can directly interact amongst themselves. This data is now presented in Figures 1J and K.

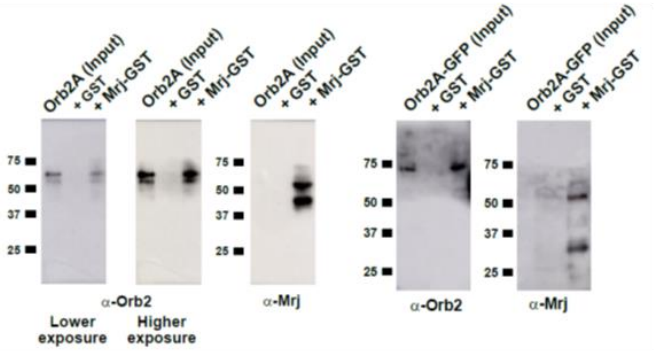

Figure 12:
Left panel (First three) : Recombinant GST-Mrj bound to Glutathione beads on incubation with purified recombinant Orb2A-His from E.coli could pulldown Orb2A. The same blot is probed first with an anti-Orb2 antibody followed by probing with an anti-Mrj antibody. GST protein bound to Glutathione beads was used as a negative control
Right panel (Last two): Recombinant GST-Mrj bound to Glutathione beads on incubation with purified recombinant Orb2A-GFP-His from Sf9 cells could pulldown Orb2A-GFP. The same blot is probed first with an anti-Orb2 antibody followed by probing with an anti-Mrj antibody. GST protein bound to Glutathione beads was used as a negative control..

2. The Huntingtin section has a few concerns. The IF doesn't show all controls and the quantification is not well done in terms of what is relevant. A major problem is the interpretation of Fig 2F. The idea is that Mrj prevents the aggregation of Htt, which is the opposite of what is observed with Orb2. The panel actually shows a large Htt aggregate instead of multiple small aggregates. This has been reported before in Drosophila and other systems with different polyQ models. Mrj and other Hsp40 and Hsp70 proteins modify Htt aggregation, but in an unexpected way. This affects the model shown in Fig. 6H. Lastly, Fig 2H and 2I show very different level of total Htt.

In Figure 2F of the previously submitted manuscript, we have shown representative images of HttQ103-GFP cells coexpressing with a control DnaJ protein CG7133-HA and Mrj-HA. In Figure 2G we quantitated the number of cells showing aggregates within the population of doubly transfected cells. On the reviewer's point of figure 2F showing large Htt aggregates instead of multiple small aggregates, we do not see a large Htt aggregate in presence of Mrj in this figure, the pattern looks diffused here and very different from the control CG7133 where the aggregates are seen. We have performed the same experiment with a different Htt construct (588 amino acids long fragment) tagged with RFP, and here also we notice in presence of Mrj, the aggregates are decreased and the expression pattern looks diffused (Supplementary Figure 4E, 4F).

If the comment on large Htt aggregates in presence of Mrj is concerning figure 2E, here we show Mrj-RFP to colocalize with the Htt aggregates. Here, even though Mrj-RFP colocalizes with Htt aggregates, it rescues the Htt aggregation phenotype as in comparison to the control CG7133, the number of cells with Htt aggregates is still significantly less here. We have added this quantitation of rescue by Mrj-RFP in the revised manuscript now. The observation of colocalization of Mrj-RFP with Htt aggregates is similar to previous reports of chaperones rescuing Htt aggregation and yet showing colocalization with the aggregates. Both Hdj-2 and Hsc70 suppress Htt aggregation and yet were observed to colocalize with Htt aggregates in the cell line model as well as in nuclear inclusions in the brain (Jana *et al*, 2000). In a nematode model of Htt aggregation, DNJ-13 (DnaJB-1), HSP-1 (Hsc70), and HSP-11 (Apg-2) were shown to colocalize with Htt aggregates and yet decrease the Htt aggregation (Scior *et al*, 2018). Hsp70 was also found to colocalize with Htt aggregates in Hela cells (Kim *et al*, 2002).

Regarding Figures 2H and 2I, while figure 2H is of an SDS-PAGE to show no difference in the levels of monomeric HttQ103 (marked with *) in presence of Mrj and the control CG7133, figure 2I is for the same samples ran in an SDD-AGE where reduced amount of Htt oligomers as seen with the absence of a smear in presence of Mrj. The apparent difference in Htt levels between 2H and 2I is due to the detection of Htt aggregates/oligomers in the SDD-AGE which are unable to enter the SDS-PAGE and hence undetected. In Supplementary Figure 4E, similar experiments were done with the longer Htt588 fragment and here we notice in the SDD-AGE reduced intensity of the smear made up of Htt oligomers, again suggesting a reduction in Htt aggregates. Thus our results are not in contradiction to previous studies where Mrj was found to rescue Htt aggregate-associated toxicity.

3. Endogenous expression of Mrj using Gal4 line: where else is it expressed in the brain / head and in muscle. Fig 3G shows no muscle abnormalities but no evidence is shown for muscle

expression. It is nice that Fig 3E and F show no abnormal aggregates in the Mrj mutant, but this would be maybe more interesting if flies were subjected to some form of stress.

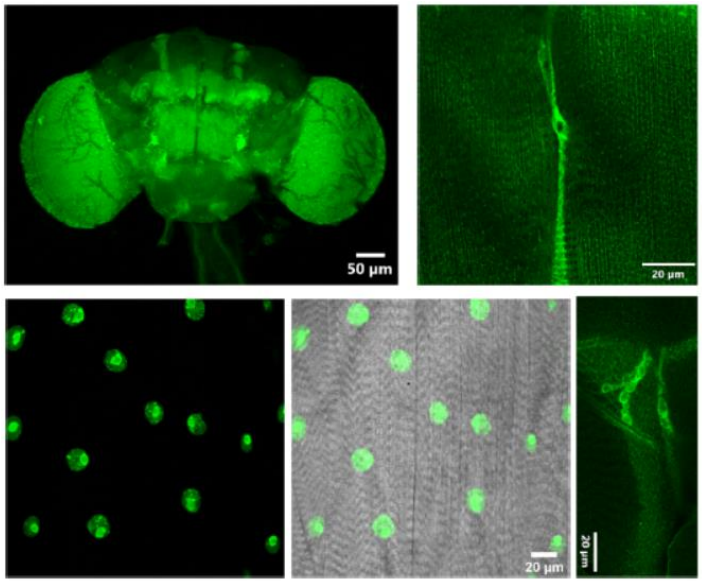

Figure 13:
Left panel (Top row) : Drosophila adult brain imge of Mrj knockout (KO) Gal4 driving UAS-CD8GFP showing enriched expression in optic lobes, olfactory lobes and the mushroom body region
Right panel (Top row): Image from the larval muscle of the same flies show expression of CD8-GFP in the muscle
Left panel (Bottom row): Image of larval muscles from Mrj KO Gal4 driving UAS-NLS-GFP shows the expression in multiple nuclei confirming the expression in muscle fibers. The right panel depicts the merge of NLS-GFP with the DIC image
Right panel (bottom row): Image of the neuromuscular junction from larvae of Mrj KO Gal4 driving UAS-CD8GFP shows expression in the neuromuscular junction synaptic boutons

We have now added images of the brain and muscles to show the expression pattern of Mrj. Using Mrj Gal4 line and UAS- CD8GFP, we noticed enriched expression in the optic lobes, mushroom body, and olfactory lobes. We also noticed GFP expression in the larval muscles and neuromuscular junction synaptic boutons. This data is now presented in Supplementary Figures 5C, D, E, and F.

On the reviewer's point of subjecting the Mrj KO flies to some form of stress, we have not performed this. We have added in the discussions a note of caution, that loss of Mrj may still result in a protein aggregation-related disease phenotype, probably under a sensitized condition of certain stresses which is not tested in this manuscript.

4. Fig. 5B shows no Mrj detectable from head homogenates in flies silencing Mrj in neurons with Elav-Gal4. It would be nice if they could show that ONLY neurons express Mrj in the head. Also noted, Elav-Gal4 is a weak driver, so it is surprising that it can generate such robust loss of Mrj protein

We have used an X chromosome Elav Gal4 driver to drive the UAS-Mrj RNAi line and here we could not detect Mrj in the western. To address the reviewer's point on the glial contribution towards the expression of Mrj, we used a Glial driver Repo Gal4 to drive Mrj RNAi. In this experiment, we did not detect any difference in Mrj levels between the control and the Mrj RNAi line (presented now in Supplementary Figure 5G). We also used the Mrj knockout Gal4 line to drive NLS-GFP and immunostained these using a glial marker anti-Repo antibody. Here, we were able to detect cells colabelled by GFP as well as Repo, suggesting Mrj is likely to be present in the glial cells (presented now in Supplementary Figure 5H). We also looked in the literature and found a reference where it was shown that Elav protein as well as Elav Gal4 at earlier stages of development expresses in neuroblasts and all Glia (Berger *et al*, 2007). So, another possibility is when we are driving Mrj RNAi using Elav Gal4, this knocks down Mrj in both the neurons as well as in the glia.

5. Fig 4-Colocalization of Orb2 with Mrj lacks controls. The quantification could describe other phenomena because the colocalization is robust but the numbers shown describe something else.

We have now added the intensity profile and colocalization quantitation (Pearson's coefficient) in Supplemental Figures 5A and B. This quantitation is done from multiple ROI's taken from 4-6 cells. Also, to suggest the interaction of Orb2 isoforms with Mrj, we are not depending on colocalization alone and have used immunoprecipitation experiments to support our observations.

6. Fly behavior. The results shown for Mrj RNAi alleles is fine but it would be more robust if this was validated with the KO line AND rescued with Mrj overexpression.

We have now performed memory assays with the Mrj knockout. Our experiments showed Mrj knockouts to show significantly decreased memory in comparison to wild-type flies at 16 and 24-hour time points (presented in Figure 6B). We have not been able to make an Mrj Knockout-UAS Mrj recombinant fly, most likely due to the closeness of the two with respect to their genomic location in the second chromosome.

Minor comments:

Please, revise minor errors, there are several examples of words together without a space.

We have identified the words without space and have corrected them now.

Intro: describe the use of functional prions. Starting the paragraph with this sentence and then explaining what prion diseases are is a little confusing. Also "prion proteins" can be confusing because the term refers to PrP, the protein found in prions.

We have now altered the introduction and have described functional prions.

Results, second subtitle in page 5. This sentence is quite confusing using prion-like twice

We have now changed the heading to "Drosophila Mrj converts Orb2A from non-prion to a prion-like state."

Page 6: "conversion from non-prion to prion-like form...". This can be presented differently. Prion-like properties are intrinsic, proteins don't change from non-prion to prion-like. They may be oligomeric or monomeric or highly aggregated but the prion-like property doesn't change

We agree with the reviewer's point of Prion-like properties are intrinsic, but the protein might or might not exist in the prion-like state or confirmation. When we are using the term conversion from non-prion to prion-like form we mean to suggest a conformational conversion leading to the eventual formation of the oligomeric species. We also noted the terminology of non-prion to prion-like state change is used in several papers (Satpute-Krishnan & Serio, 2005; Sw & Yo, 2012; Uptain *et al*, 2001).

Scale bars and text are too small in several figures

We have now mentioned in the figure legends the size of the scale bars. For several images, we have made the scale bars also larger.

Not sure why Fig 4C is supplemental, seems like an important piece of data.

We have kept this data in the supplemental data as we performed this experiment with recombinant protein which is tagged with 6X His and we are not sure if this high degree of oligomerization/aggregation of recombinant Mrj and further precipitation over time, happens inside the cells/ brain.

Intro to Mrj KO in page 7 is too long. Most of it belongs in the discussion

We have now moved the portions on mammalian DNAJB6 which were earlier in the results section to the discussions section.

Change red panels in IF to other color to make it easier for colorblind readers.

We have now changed the red panels to magenta. We apologize for our figures not being colorblind friendly earlier.

The discussion is a little diffuse by trying to compare Orb2 with mammalian prions and amyloids and yeast prions.

We looked into the functional prion data and couldn't find much on chaperone-mediated regulation of these. Also, we felt comparing with the amyloids and yeast prions brings out the contrast with respect to the Mrj-mediated regulatory differences between the two.

Reviewer #3 (Significance (Required)):

This is a paper with a broad scope and approaches. The paper describes the role of Mrj in the oligomerization of Orb2 by protein biochemistry techniques and determine the role of loss of Mrj in the mushroom bodies in fly behavior.

The audience for this content is basic research and specialized. The role of Mrj in Orb2 aggregation and function sheds new light on the mechanisms regulating the function of this protein involved in a novel mechanism of learning and memory.

References:

Bengoechea R, Findlay AR, Bhadra AK, Shao H, Stein KC, Pittman SK, Daw JA, Gestwicki JE, True HL & Weihl CC (2020) Inhibition of DNAJ-HSP70 interaction improves strength in muscular dystrophy. *J Clin Invest* 130: 4470–4485

Berger C, Renner S, Lüer K & Technau GM (2007) The commonly used marker ELAV is transiently expressed in neuroblasts and glial cells in the Drosophila embryonic CNS. *Dev Dyn* 236: 3562–3568

Fayazi Z, Ghosh S, Marion S, Bao X, Shero M & Kazemi-Esfarjani P (2006) A Drosophila ortholog of the human MRJ modulates polyglutamine toxicity and aggregation. *Neurobiol Dis* 24: 226–244

Heinrich SU & Lindquist S (2011) Protein-only mechanism induces self-perpetuating changes in the activity of neuronal Aplysia cytoplasmic polyadenylation element binding protein (CPEB). *Proc Natl Acad Sci U S A* 108: 2999–3004

Hervás R, Li L, Majumdar A, Fernández-Ramírez MDC, Unruh JR, Slaughter BD, Galera-Prat A, Santana E, Suzuki M, Nagai Y, *et al* (2016) Molecular Basis of Orb2 Amyloidogenesis and Blockade of Memory Consolidation. *PLoS Biol* 14: e1002361

Hervas R, Rau MJ, Park Y, Zhang W, Murzin AG, Fitzpatrick JAJ, Scheres SHW & Si K (2020) Cryo-EM structure of a neuronal functional amyloid implicated in memory persistence in Drosophila. *Science* 367: 1230–1234

Izawa I, Nishizawa M, Ohtakara K, Ohtsuka K, Inada H & Inagaki M (2000) Identification of Mrj, a DnaJ/Hsp40 family protein, as a keratin 8/18 filament regulatory protein. *J Biol Chem* 275: 34521–34527

Jana NR, Tanaka M, Wang G h & Nukina N (2000) Polyglutamine length-dependent interaction of Hsp40 and Hsp70 family chaperones with truncated N-terminal huntingtin: their role in suppression of aggregation and cellular toxicity. *Hum Mol Genet* 9: 2009–2018

Kakkar V, Månsson C, de Mattos EP, Bergink S, van der Zwaag M, van Waarde MAWH, Kloosterhuis NJ, Melki R, van Cruchten RTP, Al-Karadaghi S, *et al* (2016) The S/T-Rich Motif in the DNAJB6 Chaperone Delays Polyglutamine Aggregation and the Onset of Disease in a Mouse Model. *Mol Cell* 62: 272–283

Kim S, Nollen EAA, Kitagawa K, Bindokas VP & Morimoto RI (2002) Polyglutamine protein aggregates are dynamic. *Nat Cell Biol* 4: 826–831

Li L, Sanchez CP, Slaughter BD, Zhao Y, Khan MR, Unruh JR, Rubinstein B & Si K (2016a) A Putative Biochemical Engram of Long-Term Memory. *Curr Biol* 26: 3143–3156

Li S, Zhang P, Freibaum BD, Kim NC, Kolaitis R-M, Molliex A, Kanagaraj AP, Yabe I, Tanino M, Tanaka S, *et al* (2016b) Genetic interaction of hnRNPA2B1 and DNAJB6 in a Drosophila model of multisystem proteinopathy. *Hum Mol Genet* 25: 936–950

Liebman SW & Chernoff YO (2012) Prions in yeast. *Genetics* 191: 1041–1072

Lu W-H, Yeh N-H & Huang Y-S (2017) CPEB2 Activates GRASP1 mRNA Translation and Promotes AMPA Receptor Surface Expression, Long-Term Potentiation, and Memory. *Cell Rep* 21: 1783–1794

Prusiner SB (2001) Neurodegenerative Diseases and Prions. *New England Journal of Medicine* 344: 1516–1526

Satpute-Krishnan P & Serio TR (2005) Prion protein remodelling confers an immediate phenotypic switch. *Nature* 437: 262–265

Scior A, Buntru A, Arnsburg K, Ast A, Iburg M, Juenemann K, Pigazzini ML, Mlody B, Puchkov D, Priller J, *et al* (2018) Complete suppression of Htt fibrilization and disaggregation of Htt fibrils by a trimeric chaperone complex. *EMBO J* 37: 282–299

Si K (2015) Prions: what are they good for? *Annu Rev Cell Dev Biol* 31: 149–169

Si K, Choi Y-B, White-Grindley E, Majumdar A & Kandel ER (2010) Aplysia CPEB can form prion-like multimers in sensory neurons that contribute to long-term facilitation. *Cell* 140: 421–435

Si K, Lindquist S & Kandel ER (2003) A neuronal isoform of the aplysia CPEB has prion-like properties. *Cell* 115: 879–891

Sw L & Yo C (2012) Prions in yeast. *Genetics* 191

Thiruvalluvan A, de Mattos EP, Brunsting JF, Bakels R, Serlidaki D, Barazzuol L, Conforti P, Fatima A, Koyuncu S, Cattaneo E, *et al* (2020) DNAJB6, a Key Factor in Neuronal Sensitivity to Amyloidogenesis. *Mol Cell* 78: 346-358.e9

Uptain SM & Lindquist S (2002) Prions as protein-based genetic elements. *Annu Rev Microbiol* 56: 703–741

Uptain SM, Sawicki GJ, Caughey B & Lindquist S (2001) Strains of [PSI(+)] are distinguished by their efficiencies of prion-mediated conformational conversion. *EMBO J* 20: 6236–6245

Watson ED, Mattar P, Schuurmans C & Cross JC (2009) Neural stem cell self-renewal requires the Mrj co-chaperone. *Dev Dyn* 238: 2564–2574

Wickner RB (2016) Yeast and Fungal Prions. *Cold Spring Harb Perspect Biol* 8: a023531

Wickner RB, Edskes HK, Maddelein ML, Taylor KL & Moriyama H (1999) Prions of yeast and fungi. Proteins as genetic material. *J Biol Chem* 274: 555–558

Wickner RB, Masison DC, Edskes HK & Maddelein ML (1996) Prions of yeast, [PSI] and [URE3], as models for neurodegenerative diseases. *Cold Spring Harb Symp Quant Biol* 61: 541–550
