# Supplementary material for: Mrj is a chaperone of the Hsp40 family that regulates Orb2 oligomerization and long-term memory in Drosophila
Source: PLoS Biol. 2024 Apr 22;22(4):e3002585. doi: 10.1371/journal.pbio.3002585 (PMC11034981; doi:10.1371/journal.pbio.3002585)
Supplement: Attachment — Submitted filename: Response to reviewers_plosbioDec11.pdf [file pbio.3002585.s014.pdf]

Response to reviewers' comments

We would like to thank the reviewers for their extensive review of our manuscript and constructive criticism. We have attempted to address the points raised in the reviewer's comments, have performed additional experiments, and have edited the text of the manuscript to explain these points. We have submitted a version of the manuscript with track change mode to highlight the changes made. Please see below, our point-by-point response (in blue) to the reviewer's comments (in red). In the submitted revised manuscript, some figure numbers have changed from the prior reviewed version, and the following are the new figures which are added during the revision.

New figures:

Figure 2E, F, L, M
Figure 4E, K
Figure 5J, L
Supplementary Figure 5A
Supplementary Figure 6A, B

Reviewer #1:

The authors have send in a revision in which they mostly adequately dealt with my comments (see below, with my reply in red).

We thank the reviewer for the constructive suggestions and for allowing us to address and explain the questions raised during the review process.

Figure 1 (plus related Supplemental figures):

• There seem to be two isoforms of Mrj (like what has been found for human DNAJB6). I find it striking to see that only (preferentially?) the shorter isoform interacts with Orb2. For DNAJB6, the long isoform is mainly related to an NLS and the presumed substrate binding is identical for both isoforms. If this is true for Dm-Mrj too, the authors could actually use this to demonstrate the specificity of their IPs where Orb2 is exclusively non-nuclear? "Well addressed."

• I would be interested to know a bit more about the other 5 JDPs that are interactors with Orb2: are the human orthologs of those known? It is striking that these other 5 JDPs interact with Orb2 in Dm (in IPs) but have no impact on Sup35 prion behavior. Importantly, this does not imply they may not have impact on the prion-like behavior of other Dm substrates, including Dm-Orb2.

• "Well addressed. I would like the authors to add the table as supplement to the paper as it is valuable information for follow-up studies. I actually agree that it goes beyond the scope of this paper to show what the biological relevance is of the interaction between these other JDPs and Dm-Orb2."

According to the reviewer's suggestion, we have now added the table as a supplement to the paper.

• The data in panels H,I indeed suggest that Mrj1 alters the (size of) the oligomers. It would be important to know what is the actual physicochemical change that is occurring here. The observed species are insoluble in 0.1 % TX100 but soluble in 0.1% SDS, which suggest they could be gels, but not real amyloids such as formed by the polyQ proteins that require much higher SDS concentrations (~2%) to be solubilized. This is relevant as Mrj1 reduces polyQ amyloidogenesis whereas is here is shown to enhance Orb2A oligomerization/gelidification. In the same context, it is striking to see that without Mrj the amount of Orb2A seems drastically reduced and I wonder whether this might be due to the fact that in the absence of Mrj a part of Orb2A is not recovered/solubilized due to its conversion for a gel to a solid/amyloid state? In other words: Mrj1 may not promote the prion state, but prevents that state to become an irreversible, non-functional amyloid?

"I am still a bit confused, also because of the complexity of the answer in relation to my question. I do understand and accept that the authors cannot pinpoint (at this stage) what actual physicochemical states of the Orb2A is in under the various condition. What I still do not understand is why there is less Orb2A in the absence of Mrj1. What I also still think could be the case is that Mrj1 is not driving oligomerization per se, but that its (early) interactions with Orb2A affect the oligomerization process (up until becoming amyloids). So, I suggest that the authors remain somewhat open-minded about this alternative explanation and mention it in their paper."

In the absence of Mrj, the total Orb2 levels are not changed but the amount of Orb2 oligomers is decreased. In the differential extraction experiments with SDS buffers, we notice in the absence of Mrj, there is more Orb2 in the soluble fraction (NP40 extracted) and less of it in the SDS extracted fractions. While one possibility that we have originally suggested is Mrj is driving the oligomerization process of Orb2, we also agree with the reviewer's alternative suggestion that early interactions with Mrj might be affecting the oligomerization process of Orb2 up until becoming amyloid. We have now added the following statement in our results section:

An alternate possibility is early interactions of Mrj with Orb2 affect the latter's oligomerization process, by regulating a conformational change in Orb2 which now makes it an amenable unlocked substrate on which other chaperones or other regulatory proteins might act to drive the oligomerization process.

Figure 2 (plus related Supplemental figures):

• It may be good for clarity to refer to the human Mrj as DNAJB6 according to the HUGO nomenclature. Also, the first evidence for its oligomerization was by Hageman et al 2010. "Well addressed."

• It is striking to see that Mrj co-IPs with Hsp70AA, Hsp70-4 but not Hsp70Cb. The fact that interactions were detected without using crosslinking is also striking given the reported transient nature of J-domain-Hsp70 interactions Together, this may even suggest that Mrj-1 is recognized as a Hsp70 substrate (for Hsp70AA, Hsp70-4 but not Hsp70Cb) rather than as a co-chaperone. In fact, a variant of Mrj-1 with a mutation in the HPD motif should be used to exclude this option.

"I understand and am aware of the fact that in some cases JDP-Hsp70 interactions are seen in co-IP even without crosslinking, but also here these may be indirect via binding to substrates for which different Hsp70 may have different specificities (rather than Mrj-1 specifically binding to specific Hsp70 isoforms). In any case, I feel an experiment with an HDP motif mutant would be essential in my view to make any claims on J-domains showing specific binding to only a subset of Hsp70 isoforms."

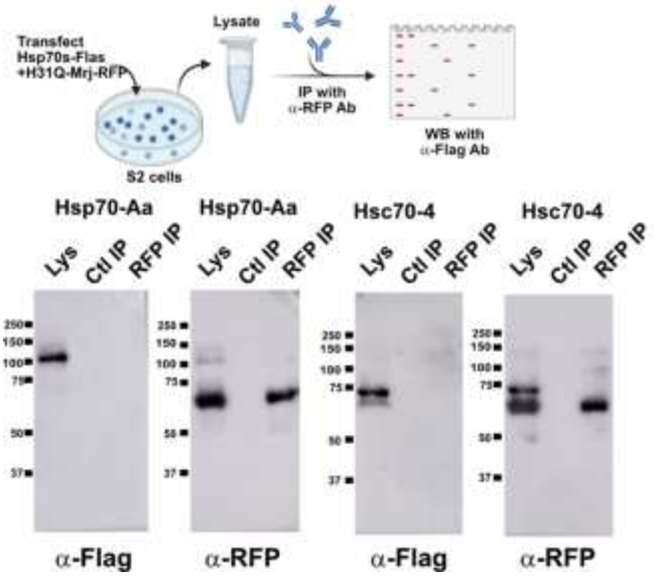

We have now mutated the HPD motif mutant of Mrj to QPD and used this construct in IP with the Hsp70Aa and Hsc70-4. In our IP's while we could detect the pulldown of Mrj happening, we were unable to detect the presence of Hsp70Aa and Hsc70-4 in the immunoprecipitate suggesting a lack of interaction between two Hsp70's and the HPD mutated Mrj. This is presented in Figure 2E, F in the revised manuscript.

• The rest of these data reconfirm nicely that Mrj/DNAJB6 can suppress polyQ-Htt aggregation. Yet note that in this case the oligomers that enter the agarose gel are smaller, not bigger. This argues that Mrj is not an enhancer of oligomerization, but rather an inhibitor of the conversion of oligomers to a more amyloid like state.

"I was indeed wrong regarding my interpretation of figure 2I (I assume incorrectly that the lane marked with Mrj was the knockout). However, the interpretation still is (even more so now) skewed by the fact that the total amount of polyQ detected in the case of cells with Mrj is so much less that in the controls. So, whereas the other anti-aggregation data for polyQ are still solid and confirming literature data, I still feel one cannot interpret that data as to suggest that Mrj is an "enhancer of oligomerization" (for neither polyQ, nor for Orb2A: see above). The situation is likely more subtle."

Based on the points made by the reviewer, we have now revisited this. As we did not have a loading control and quantitation earlier, we have now repeated these experiments and provided quantitations. In our SDS-PAGE experiment, we see no significant difference between HttQ103 levels between CG7133 control and the Mrj samples (In red box). In the SDD-AGE experiments with a higher percent of agarose in the presence of Mrj, we notice two distinct bands of Htt. One faster-moving band of lighter intensity migrates faster in comparison to the lower-most band in the CG7133 lane. The other band in the Mrj lane is a bit higher but is more intense in the blots and comes at a slightly higher position (marked with a red arrow here) than the lowermost band seen in the CG7133 lane. The smear in the Mrj lane is much lighter and covers a smaller range than the CG7133 lane indicating fewer oligomers. The intense band marked by the red arrow here probably represents the collapse of the larger Htt oligomers to smaller, less diverse-sized Htt oligomers, suggesting Mrj possibly acts as an inhibitor or suppressor for HttQ103 aggregation. These are presented in Figures 2J, K, L, and M in the revised manuscript.

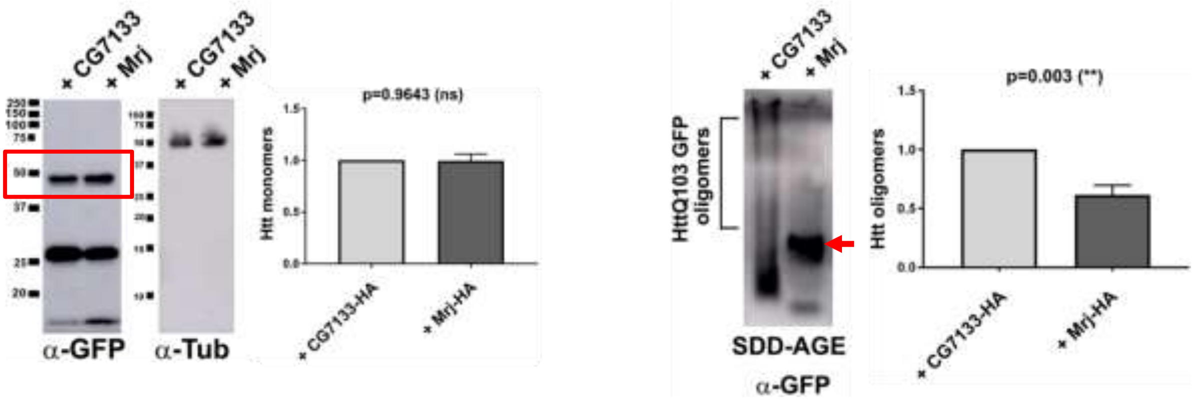

Figure 3:

• The finding that knockout of DNAJB6 in mice is embryonic lethal is related to a problem with placental development and not embryonic development (Hunter et al, 1999; Watson et al, 2007, 2009, 2011) as well recognized by the authors. Therefore, the finding that deletion of Dm-Mrj has no developmental phenotype in Drosophila may not be that surprising.

"Well addressed."

• It is a bit more surprising that Mrj knockout flies showed no aggregation phenotype or muscle phenotype, especially knowing that DNAJB6 mutations are linked to human diseases associated with aggregation (again well recognized by the authors). However, most of these diseases are late-onset and the phenotype may require stress to be revealed. So, while important to this MS in terms of not being a confounder for the memory test, I would like to

ask the authors to add a note of caution that their data do not exclude that loss of Mrj activity still may cause a protein aggregation-related disease phenotype. Yet, I also do think that for the main message of this MS, it is not required to further test this experimentally. “Well addressed.”

Figure 4:

• IPs were done with Orb2A as bite and clearly pulled down substantial amounts of GFP-tagged Mrj. For interactions with Orb2B, a V5-tagged Mrj was use and only a minor fraction was pulled down. Why were two different Mrj constructs used for Arb2A and Orb2b? “Well addressed.”

• In addition, I think it would be important what one would see when pulling on Mrj1, especially under non-denaturing conditions and what is the status of the Orb2 that is than found to be associated with Mrj (monomeric, oligomeric and what size).

“Well addressed: nice additional data!.”

• This also relates to my remark at figure 1 and the subsequent fractionation experiments they show here in which there is a slight (not very convincing) increase in the ratio of TX100-soluble and insoluble (0.1% SDS soluble) material. My question would be if there is a remaining fraction of 0.1% insoluble (2% soluble) Orb2 and how Mrj affects that? As stated before, this is (only) mechanistically relevant to understanding why there is less oligomers of Orb2 in terms of Mrj either promoting it or by preventing it to transfer from a gel to a solid state. The link to the memory data remains intriguing, irrespective of what is going on (but also on those data I do have several comments: see below).

“Well addressed (still see comments above).”

• I also find the sentence that “Mrj is probably regulating the oligomerization of endogenous Orb2 in the brain” somewhat an overstatement. I would rather prefer to say that the data show that Mrj1 affects the oligomeric behavior/status of Orb2.

“Well addressed.”

Figure 5:

• To my knowledge, the Elav driver regulates expression in all neurons, but not in glial cells that comprise a significant part of the fly heads/brain. The complete absence of Mrj in the fly-heads when using this driver is therefore somewhat surprising. Or do we need to conclude from this that glial cells normally already lack Mrj expression?

“Well addressed.”

• Why not use these lines also for the memory test for confirmation? I understand the concerns of putative confounding effects of a full knockdown (which were however not reported), but now data rely only on the mushroom body-specific knockdown for the 201Y Gal4 line, for which the knockdown efficiency is not provided. But even more so, here a temperature shift (22oC-30oC) was required to activate the expression of the siRNA. For the effects of this shift alone no controls were provided. The functional memory data are nice and consistent with the hypothesis, but can it be excluded that the temperature shift (rather than the Mrj) knockdown

has caused the memory defects? I think it is crucial to include the proper controls or use a different knockdown approach that does not require temperature shifts or even use the knockout flies.

“Well addressed. Nice new, consistent data!”

Figure 6:

The finding of a co-IP between Rpl18 and Mrj (one-directional only) by no means suffices to conclude that Mrj may interact with nascent Orb2 chains here (which would be the relevant finding here). The fact that Mrj is a self-oligomerising protein (also in vitro, so irrespective of ribosomal associations!), and hence is found in all fractions in a sucrose gradient, also is not a very strong case for its specific interaction with polysomes. The finding that there is just more self-oligomerizing Orb2A co-sedimenting with polysomes in sucrose gradients neither is evidence for a direct effect of Mrj enhancing association of Orb2A with the translating ribosomes even though it would fit the hypothesis. So all in all, I think the data in this figure and non-conclusive and the related conclusions should be deleted.

“Well addressed with nice additional data.”

Overall, provided that proper controls/additional data can be provided for the key experiments of memory consolidation, I find this an intriguing study that would point towards a role of a molecular chaperone in controlling memory functions via regulating the oligomeric status of a prion-like protein and that is worthwhile publishing in a good journal.

However, in terms of mechanistical interpretations, several points have to be reconsidered (see remarks on figure 1,4); this pertains especially to what is discussed on page 13. In addition, I’d like the authors to put their data into the perspective of the findings that in differentiated neurons DNAJB6 levels actually decline, not incline (Thiruvalluvan et al, 2020), which would be counterintuitive if these proteins are playing a role as suggested here in memory consolidation.

“See my remaining comments above. I think a slight rephrasing of a few of the interpretations would suffice for me to advise acceptation of this nice MS.”

We have now added the HPD mutant experiment, presented a new experiment on the effect of Mrj on Htt aggregation, and an alternate interpretation of the role of Mrj on Orb2 oligomerization. We hope these additions answer the reviewer’s points adequately.

Reviewer #2:

The authors have addressed all my concerns about the addition of data (to figure 2G, supplemental figures 5A, B and supplemental figure 6C) and expansion of discussion about Mrj's chaperone functions.

We thank the reviewer for the constructive suggestions and for being supportive of our manuscript.

Two minor points:

- It was mentioned that the Mrj knockout line was created in the w[1118] background. In Fig 6B, is w[1118] the wild type control? Similarly, it appears from the Methods section that the Mrj RNAi and the GFP RNAi lines have the same genetic background. If these are correct, it

would be good to point out that the genetic background of the lines being compared in these experiments are equivalent.

We thank the reviewer for raising this point. The knockout line was created in the background of w1118, the same as our wild-type control strain which was used in all the biochemistry experiments. For the behavior experiments, we even went further and cantonized the Mrj knockout strain by crossing it with wildtype CsBz line for several generations and using CsBz as the control. It was necessary to change the background from w1118 to CsBz, as white-eye flies have been reported earlier to have reduced learning. So, both the Mrj KO and the wild-type flies used here are of equivalent background. Similarly, the Mrj RNAi and the control line are also of equivalent background

- As the other reviewers have pointed out, it would have been stronger to demonstrate that the phenotypes of the Mrj knockout line can be rescued by overexpression. A discussion around why this was not possible would be good to include in the paper.

We planned to do the transgene rescue experiment but we were unable to do this experiment due to technical reasons. Both the Mrj Knockout Gal4 line and the UAS Mrj line were in the second chromosome and we attempted to make a recombinant, however, we were unable to get this recombinant, which is most likely due to both the lines being very close in their genomic location, thus reducing any chances of recombination.

Reviewer #3:

This is a second version of this manuscript which identifies a chaperone of the Hsp40 family (Mrj) that binds Orb2 and modulates its oligomerization, which is critical for Orb2 function in learning and memory in Drosophila. The article starts with a screen of Hsp40 and Hsp70-family proteins that bind Orb2 and the identification of Mrj as the one with the specific interaction. Following the generation of a KO Mrj mutant, they show that the silencing of Mrj in the mushroom body gamma neurons results in weaker memories in a courtship paradigm. The data is novel, interesting, consistent, and generally supportive of the hypothesis. The article also has some concerns as presented. This is an ambitious article that needs additional work before publication.

Several experiments still lack the expected rigor, including controls and quantification. The observations are quite interesting and expansive. As the authors attempt to cover so much ground with Orb2, Htt-103Q, biochem, cell bio, and behavior, the experiments are conducted without the necessary rigor, leaving questions unanswered along with concerns for the interpretation of data. The strength is clearly in the biochemistry with other approaches leaving extensive room for criticism and interpretation.

Importantly, key controls are still missing in the behavioral assays. These assays are very sensitive to genetic background. Multiple controls are necessary to support the claim that an effect on memory is detected both in the Mrj KO experiments and in the RNAi assays. Mrj overexpression is also critical to support the claims throughout the manuscript that Mrj promotes Orb2 oligomerization, which should strengthen memory formation. In addition, no explanation or mechanism are given for why only long-term memory is affected in the Mrj mutant background. These are time-consuming experiments and hard to replicate but controls are critically needed to support the conclusions of the study.

We thank the reviewer for the detailed comments and the points raised in the review. We have attempted to address the reviewer’s comments below.

Detailed comments:

I am still not convinced about the ability of Mrj to eliminate Htt-103Q nuclear aggregates.

The authors point to literature supporting this anti-aggregation effect of chaperones, but there is significant literature supporting a pro-aggregation effect of chaperones, possibly as a defensive mechanism. This may be an artifact observed in non-physiological models that are comparable to the model shown here: Warrick, Bonini, Nat Gen 1999-No change in polyQ aggregation with Hsp70 co-expression; Fernandez-Funez, Botass, Nature 2000-more compact polyQ aggregates with DNAJ1 co-expression. Other later reports

Fig 2E show multiple aggregates to show the colocalization of Orb2A and Mrj. Figs 2F and G show drastically decreased Htt aggregates. 2E, F and G don't seem to correlate too well. Counting the number of aggregates per cell could help with this specific point. Also, the middle panel of F still shows a large NI.

Both the papers cited by the reviewer are based on proteins that form nuclear aggregates. The Warrick Bonini paper shows even though Hsp70 rescues the toxicity associated with MJDtrQ78, it does not decrease the nuclear inclusions. This suggests the mechanism of Hsp70-mediated rescue is not through altering the MJD nuclear aggregates but this doesn't say the chaperone has a pro-aggregation role. The Fernandez-Funez, Botas paper shows that DnaJ1 rescues the Ataxin1-82Q, and in this case, the nuclear aggregates look more compact and less invasive similar to the non-pathogenic Ataxin1-30Q. Here also, we think of this not as a pro-aggregation role of the chaperone but as something that limits the aggregates.

For, both the Htt constructs (103Q and 138Q) used in this manuscript, we do not observe them to form nuclear aggregates. In Figure 2E (now Figure 2G in the revised manuscript) we do not show colocalization between Orb2A and Mrj, but between HttQ103 and Mrj, and we sincerely hope the point by the reviewer is a typo. In the graph in Figure 2G (now Figure 2I in the revised manuscript), these cells are the ones that are part of the around 20% of cells that show Htt aggregates in them. Figure 2F (now Figure 2H) shows representative images of what happens in the majority of cells where, in the presence of Mrj (both HA and RFP tagged) there is a significant decrease in the number of cells containing Htt aggregates as shown in the quantitation graph in Figure 2G (now Figure 2I). We are also unable to see any large NI in the middle panel of F (also pasted below, now Figure 2H), so we are a bit confused about the reviewer's comments here.

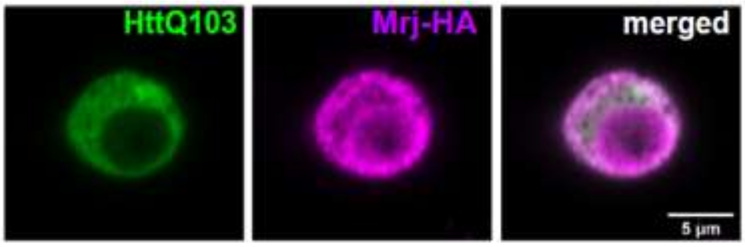

We also have pasted below a representative image of another cell to show diffused HttQ103 in presence of Mrj.

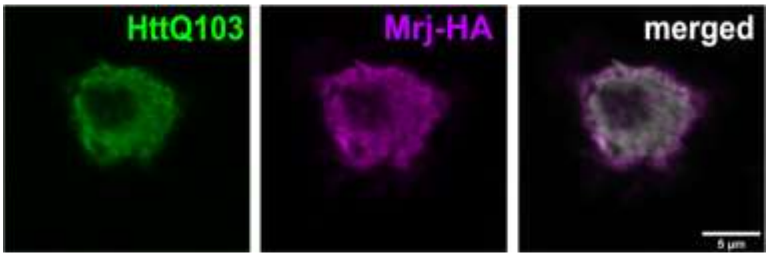

On a more practical point, how does Mrj Lower Htt-109Q aggregation on its own? Hsp40 can bind client proteins but the catalytic activity is provided by Hsp70. Overexpressing Hsp40 can bind substrates and alter their dynamics but should not be sufficient on its own to remove Htt-103Q aggregates.

Our observation of Drosophila Mrj alone lowering Htt aggregates is not too different from the previous observations with both Drosophila and mammalian Mrj/DnaJB6. In Drosophila expression of Mrj alone was reported to reduce poly-Glutamine associated toxicity and aggregation [1]. During the mammalian Mrj's initial characterization work, it was shown that the expression of Mrj alone can lower the Htt aggregates [2]. Further work from the Kampinga lab supported that Mrj/DnaJB6 alone can reduce Htt aggregates and they also showed for lowering Htt aggregation, the Hsp70 interacting domain of Mrj is not needed [3]. We have cited these papers in the manuscript. There might be other proteins including Hsp70 which may even further enhance the protective role of Mrj concerning the Htt aggregates, but in our experiments, as compared to the control we see a protective role of Mrj on its own, and are reporting this.

2H: The WB shows similar expression but no loading control. I am curious, what are the different bands in 2H, particularly the one running at 250?

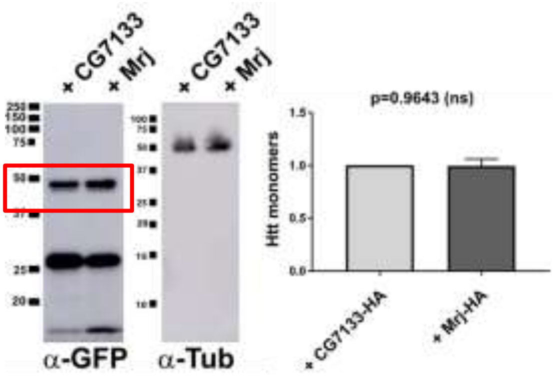

We think the band at 250 (previously Figure 2H in the earlier manuscript) is probably a higher oligomeric form of Htt which can enter the resolving gel and is detected by the antibody. As this experiment lacked a loading control and quantitation, we have now repeated the experiments and provided new data with loading control and quantitation data (Figure 2J, K in the revised manuscript). For the Tubulin blot, we used a different percent polyacrylamide gel as the Htt band and Tub bands come around the same size. Here we do not see any significant difference in total Htt (in red box here) in the presence of Mrj and the control.

2I: shows Lower oligomers in the presence of Mrj but Total Htt is much lower. Where is the rest of the protein? This is not quantified either

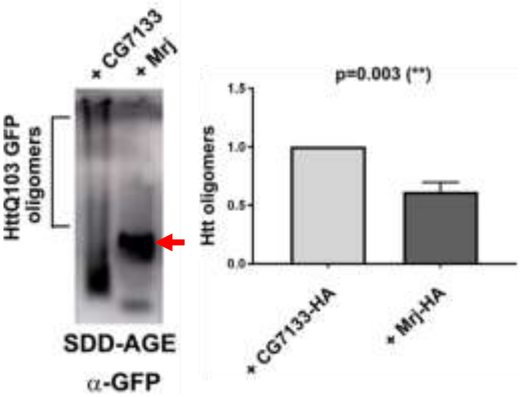

We thank the reviewer for raising this point and based on this we reinvestigated this. We have now repeated this experiment of SDD-AGE with a higher percentage of agarose. Compared to the control lane (CG7133), in the presence of Mrj, we see lighter smear (marked as Htt Oligomers) and therefore lesser oligomers. In the presence of Mrj, we notice two distinct bands of Htt. One faster-moving band of lighter intensity migrates faster in comparison to the lowermost band in the CG7133 lane. The other band in the Mrj lane is a bit higher (marked with a red arrow here) but is more intense in the blots and comes at a slightly higher position than the lowermost band seen in the CG7133 lane. The SDD-AGE and SDS PAGE experiments suggest the total HttQ103 is of similar levels between the CG7133 and Mrj

samples. We have provided quantitation of the Htt Oligomers based on the intensity of the smear and this suggests there are fewer Htt oligomers in the presence of Mrj. The band marked with a red arrow here probably represents the collapse of the larger Htt oligomers to smaller, less diverse-sized Htt oligomers in presence of Mrj. This is now presented in Figures 2L and M in the revised manuscript.

To complete this part of the story, it would be nice if the authors showed the consequence of Mrj KO on Htt-103Q.

Our experiments are done in S2 cells with transfection and we do not have a S2 cell knockout line for Mrj to try this experiment. However, the effect of Mrj/DnaJB6 KO on Htt aggregates in mammalian cells is previously shown in two papers [4,5], and in both, it shows an increase in Htt aggregates in these cells, and we have cited both these papers in the manuscript.

Prion-like terminology: Sorry to make a large point out of this detail. if a protein has a prion-like domain (per the authors), it cannot "convert to a prion-like state". This terminology is misleading even if it can be found in the Orb2 field. Prion-like properties refer to the ability to transition from soluble to misfold and oligomerize, properties that are encoded in the protein, meaning that a soluble state still has prion-like properties. Since the authors make significant references to prions and amyloids, the proper use of the terminology would be desirable. There is an overuse of the "prion" terminology, perhaps to increase the impact of the paper, when the actual intent is to identify aggregated conformations. Later on, Htt aggregates are called oligomers, but Htt does not seem to have a prion-like state. Consistent nomenclature can help avoid distractions and it takes nothing from the merits of the paper.

We agree for the traditional PrP prion, the protein transitions from soluble to misfolded oligomers and then to aggregates. However, Orb2 is different and is classified as a functional prion, and in the prion-like oligomeric state, it does not misfold but helps in the physiological function of maintenance of memory. Functional prions are now a flourishing field with many proteins being discovered which in their prion-like conformational state help in the regulation of physiological functions in cells [6–16]. Our use of the term prion-like is based on the use of the prion-like terminology in earlier papers from Eric Kandel, Susan Lindquist, and Kausik Si's lab where the concept of CPEB/ Orb2 being a prion-like protein was initiated and established [17–32].

End of page 4: "… Orb2A's prion-like conversion". Should be conformational conversion

We have changed this to prion-like conformational conversion.

Fig 1J and K: what are the two bands seen in the membranes incubated with anti-Mrj? The second band has a different MW in J and K, it is unclear if this is an Mrj byproduct (fragment?)

We think these different bands represent different breakdown products of Mrj in independent experiments.

Supp fig. 5: Mrj expression / distribution: it would be nice to see the description of Mrj distribution more aligned with the discussion of the Mrj KO line: looking for abnormal phenotypes in brain and muscle makes sense if Mrj is expressed in these tissues.

According to the reviewer's suggestions, we have now moved our observations on Mrj's expression to the section where we describe the characterization of Mrj KO Gal4 line, as in this section we discuss abnormal phenotypes in the brain and muscles.

Fig. 4A-D: These images need controls and quantifications. All images shown have coexpression of Orb2A and Mrj. It would be nice to see controls, particularly of Orb2A and B alone. Is the distribution of Orb2A and / or Orb2B altered by Mrj? Is Mrj contributing to the aggregation of Orb2B since it does not seem to aggregate on its own?

In our previous submissions, we put images of cells co-expressing Orb2 and Mrj, as our focus was on the colocalization and interaction between them. We have now provided individual images of S2 cells expressing GFP-tagged constructs of Orb2A, Orb2A325, Orb2AΔ162, Orb2B, and Orb2B478 in the Supplementary Figure 5A.

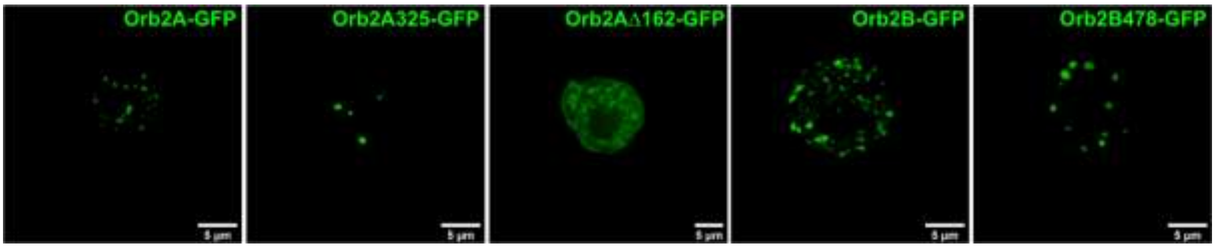

On the point of if Mrj is altering the distribution of Orb2A/Orb2B, contributing to their aggregation, we are not drawing any conclusion on these based on the imaging experiments, the reason being the heterogeneity in terms of expression levels that we see in our transient transfections in S2 cells. For only Orb2B-GFP expressing cells, some cells are showing Orb2B aggregates based on higher expression and for the colocalization experiment, we have imaged these.

Fig 4B: it seems that the aggregation pattern of the Orb2A325 mutant has changed compared to WT to the formation of fewer but larger aggregates. If this is a consistent change, it is quite relevant. This mutant should be shown alone to determine its aggregation qualities.

The Orb2A325 GFP mutant has a deletion of the RNA binding domain. In comparison to the full-length Orb2A GFP, this shows fewer aggregates but of bigger sizes in cells. We have provided an image of this now in the supplemental figure 5A in the revised version. On co-expression with Mrj, we don't think this is changing the distribution of the Orb2A325 GFP aggregates. The idea of doing this experiment was to see the colocalization between these proteins with Mrj-RFP and to use this in immunoprecipitation experiments to check for interaction.

Page 10: Mrj interacts with both the Orb2A and Orb2B isoforms, and this interaction is independent of the RNA binding domain." Technically, the authors only know this for Orb2A and this is relevant because Orb2A and B have different behaviors.

We agree with the reviewer's suggestion, that our experiments show only the interaction of Orb2A with Mrj is independent of its RNA binding domain since we have not used an RNA binding domain deleted construct of Orb2B to show the same. To address this we have now made an RNA binding domain construct of Orb2B (Orb2B478) and used this to perform immunoprecipitation with Mrj. In this experiment, we could detect a positive interaction between Mrj and the RNA binding domain deleted form of Orb2B. We also noticed colocalization

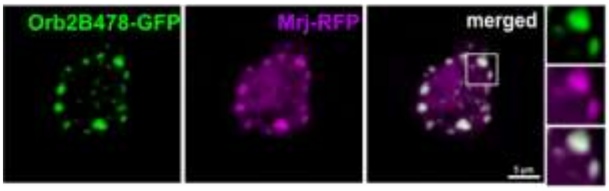

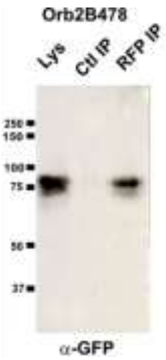

between Mrj and Orb2B478. Together this supports the point that Mrj interacts with both the Orb2A and Orb2B isoforms and this interaction is independent of the RNA binding domain.

Fig. 4L: the text describing this panel may need some clarification. "…the presence of both monomeric Orb2B and oligomeric Orb2 in the immunoprecipitated…". I am not sure how the authors distinguish between monomeric Orb2B and oligomeric Orb2 (?) isoforms in the gel after probing with the Orb2 antibody. This may be a typo and refer only to oligomeric vs monomeric Orb2. But since the top of the middle paragraph starts with both Orb2A and B isoforms, this last sentence is confusing.

Monomeric Orb2B is detected in the western blot after SDS-PAGE. We mention this as Orb2B based on the molecular weight. When we run the same IP in SDD-AGE, on probing with anti Orb2 antibody, we detect a smear. This we refer to as Orb2 oligomer as here we cannot distinguish between Orb2A and Orb2B. The oligomeric population has been previously shown to contain both Orb2A and Orb2B and its formation of these oligomers was found to be dependent on Orb2A [28].

Fig. 5A: "the detectable form is Orb2B representing its relatively higher abundance in the brain". How do they know this? Are the two isoforms differentiated in WB by electrophoretic mobility?

A previous paper [28] discusses the pieces of evidence based on which it is said that Orb2B is relatively of much higher abundance than Orb2A. While Orb2B and Orb2A on overexpression can be distinguished by electrophoretic mobility, for endogenous Orb2A and Orb2B, the detectable form from fly heads in westerns is Orb2B.

Fig 5C-D: Now we see here 2 bands that we do not see in Fig 5A. The quantification in 5A makes the point that there is less insoluble Orb2 in the -/-. It would be critical to quantify separately the soluble and the insoluble fractions. If there is less insoluble Orb2, there should be more soluble. Calculating the fraction only indicates that one of them changes but we don't know about the other. This is quite critical in these experiments because this serves as an internal control, just like showing the Tub does not accumulate in the insoluble fraction, which shows good technique in the separation. Since the p = 0.038, quite borderline significance, understanding the differences in each fraction separately is critical.

In Fig5C-D, we have shown a ratio of soluble/insoluble which uses the quantitation of both the fractions to get the ratio. The band seen here represents Orb2B (sometimes we see this as a doublet). Later, in Figures 5K and 5L, we did an experiment where we used three buffer conditions with different detergent concentrations. For the soluble part's lysis, we used a buffer containing NP-40 and Triton X-100, next the leftover pellet was solubilized in a buffer containing 0.1% SDS and again the remaining pellet from this was solubilized in a 2% SDS containing buffer and all these three fractions were checked in SDS-PAGE. Here on quantitation, we notice that in the soluble fraction for Mrj knockout, there is more soluble Orb2B and less of this in the SDS extracted insoluble fractions whereas for the wild type, there is less Orb2B in the soluble fraction and more in the SDS insoluble fractions. When we ran these in SDD-AGE we a similar pattern for Orb2 oligomers (Figure 5I and J) of more oligomers in the soluble fraction and less in the 2% SDS insoluble fraction.

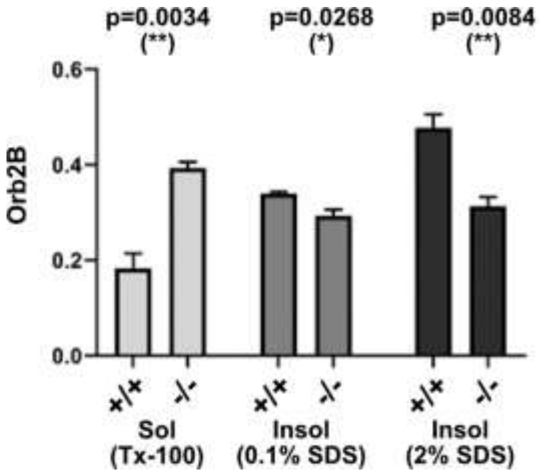

Figure 6: 6A controls still missing. The background of the Mrj-/- is different from the WT and this can affect the behavioral assay. Isogenic background, adding a line with the same background as the -/- but with normal Mrj, or adding more controls, like a +/- are necessary here.

The background of Mrj-/- and Wt are not different. While the Mrj-/- was made in the w1118 background, for the behaviour experiments, we put this in the same w+ background as the wild type and also cantonised the flies so that both the control and test have the same background. The transgenic rescue has not been feasible due to a technical problem of us not being able to make the recombinant line of Mrj KO with UAS-Mrj where both of these are in the second chromosome.

Based on the reviewer's suggestion of an experiment with Mrj-heterozygous flies, we have done one experiment using a P element line KG04490, which is near the Mrj locus but does not disrupt the ORF and the UTRs. We crossed the Mrj-/- line with this line, took the Mrj-/ KG04490 flies, and subjected them to a memory assay experiment. We focussed on the 16 and 24-hour time points here as at these time points Mrj-/- shows a memory deficit. In this experiment, we don't see any significant difference between the memory scores for wild type and Mrj-/ KG04490 at 16 and 24-hour time points. This suggests that the memory deficit seen in homozygous Mrj-/- flies is not seen in flies that are heterozygous for Mrj-. This data is presented in Supplementary Figure 6A.

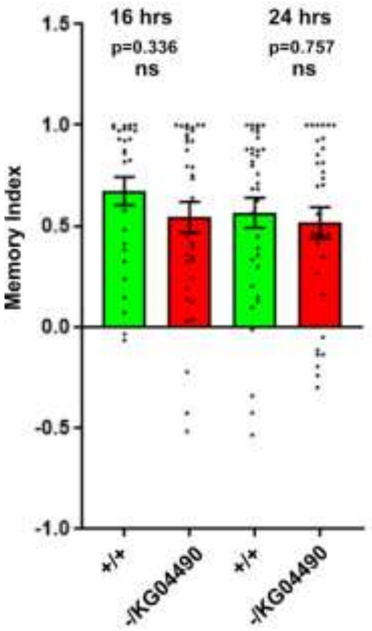

Same for 6F, more controls are needed, included non-temp shifted and other RNAi lines to account for genetic backgrounds. The mechanistic claims in the paper requires the overexpression of Mrj to promote aggregation and detect stronger memory formation.

Both the control GFP RNAi and Mrj RNAi have equivalent genetic backgrounds and are crossed to the same 201YGal4; TubGal80 ts line and their progeny (201YGal4; Tub Gal80 ts; UAS-GFP RNAi and 201YGal4; Tub Gal80ts; UAS-Mrj RNAi) is used in the memory experiments. When these flies were kept at a higher temperature which inactivates the Gal80 ts and thus allows the expression of the RNAi, we noticed memory deficit at the 16 and 24-hour time points for the Mrj RNAi in comparison to the control GFP RNAi. We have now performed the suggested control experiment using the progenies from the same genetic crosses (of the same genetic background) but without the temperature shift so that the Gal80 ts is not inactivated and it can suppress the Gal4 and thus prevent the RNAi from getting induced. In this experiment we could not see any significant difference between the two sets

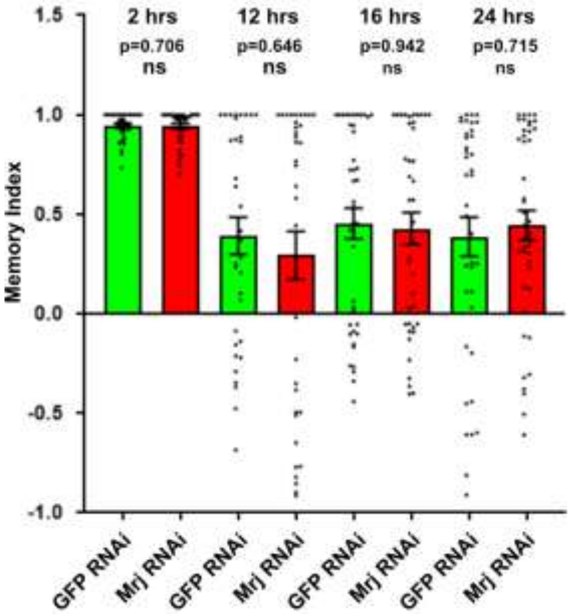

at any time point including the 16 and 24-hour time points (Supplementary Figure 6B), suggesting our observation of memory deficit happens only on inducing the Mrj RNAi.

Mrj seems to affect Long-term memory but not short-term memory: there is no mechanism for this observation. Without a clear mechanism, it may be just variability or background effects at a time when memories are much more labile.

Long-term memory mutants usually do not show short-term memory deficits. Also while long-term memory is thought to be dependent on protein synthesis, short-term memory is not. Our memory experiments with knockout and RNAi lines suggest the need for Mrj for long-term memory but not for short-term memory. While we do not know the exact mechanistic reason for this, we speculate, that one of the possible reasons for this can be due to decreased Orb2 oligomers in the brain which causes a dysfunction with the long-term memory phase but not with short-term memory. Another possibility is Mrj through its interaction with the translating ribosomes, might regulate the translation or assisted folding of a certain subset of proteins which might be needed for long-term but not short-term memory. Apart from Orb2, there are possibly other proteins, including RNA binding proteins, and translation regulators which might be regulated by Mrj.

Minor:
Fig. 5I is not quantified

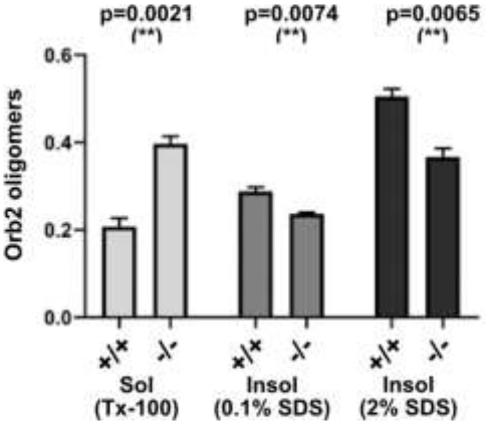

We have now added the quantitation of Figure 5I in the revised manuscript. In the quantification of Orb2 oligomers in the SDD-AGE experiment, a similar trend as seen in the earlier SDS-PAGE experiment was seen.

Capitalized words (sometimes): prions, yeast, adenine

We have now changed all these to non-capitalized form.

Please, fix the scale bars to make them legible

We have mentioned the dimensions of the scale bars in the figure legends now.

We have now performed additional experiments and presented new data and hope our revised manuscript answer the reviewer’s points adequately.

References

1. Fayazi Z, Ghosh S, Marion S, Bao X, Shero M, Kazemi-Esfarjani P. A Drosophila ortholog of the human MRJ modulates polyglutamine toxicity and aggregation. Neurobiol Dis. 2006;24: 226–244. doi:10.1016/j.nbd.2006.06.015

2. Chuang J-Z, Zhou H, Zhu M, Li S-H, Li X-J, Sung C-H. Characterization of a brain-enriched chaperone, MRJ, that inhibits Huntingtin aggregation and toxicity independently. J Biol Chem. 2002;277: 19831–19838. doi:10.1074/jbc.M109613200

3. Hageman J, Rujano MA, van Waarde MAWH, Kakkar V, Dirks RP, Govorukhina N, et al. A DNAJB chaperone subfamily with HDAC-dependent activities suppresses toxic protein aggregation. Mol Cell. 2010;37: 355–369. doi:10.1016/j.molcel.2010.01.001

4. Rodríguez-González C, Lin S, Arkan S, Hansen C. Co-chaperones DNAJA1 and DNAJB6 are critical for regulation of polyglutamine aggregation. Sci Rep. 2020;10: 8130. doi:10.1038/s41598-020-65046-5

5. Thiruvalluvan A, de Mattos EP, Brunsting JF, Bakels R, Serlidaki D, Barazzuol L, et al. DNAJB6, a Key Factor in Neuronal Sensitivity to Amyloidogenesis. Mol Cell. 2020;78: 346-358.e9. doi:10.1016/j.molcel.2020.02.022

6. Hou F, Sun L, Zheng H, Skaug B, Jiang Q-X, Chen ZJ. MAVS forms functional prion-like aggregates to activate and propagate antiviral innate immune response. Cell. 2011;146: 448–461. doi:10.1016/j.cell.2011.06.041

7. Rayman JB, Kandel ER. TIA-1 Is a Functional Prion-Like Protein. Cold Spring Harb Perspect Biol. 2017;9: a030718. doi:10.1101/cshperspect.a030718

8. Shi Y, Yuan B, Qi N, Zhu W, Su J, Li X, et al. An autoinhibitory mechanism modulates MAVS activity in antiviral innate immune response. Nat Commun. 2015;6: 7811. doi:10.1038/ncomms8811

9. Cai X, Chen ZJ. Prion-like polymerization as a signaling mechanism. Trends Immunol. 2014;35: 622–630. doi:10.1016/j.it.2014.10.003

10. Cai X, Chen J, Xu H, Liu S, Jiang Q-X, Halfmann R, et al. Prion-like polymerization underlies signal transduction in antiviral immune defense and inflammasome activation. Cell. 2014;156: 1207–1222. doi:10.1016/j.cell.2014.01.063

11. Cai X, Xu H, Chen ZJ. Prion-Like Polymerization in Immunity and Inflammation. Cold Spring Harb Perspect Biol. 2017;9: a023580. doi:10.1101/cshperspect.a023580

12. Chakrabortee S, Kayatekin C, Newby GA, Mendillo ML, Lancaster A, Lindquist S. Luminidependens (LD) is an Arabidopsis protein with prion behavior. Proc Natl Acad Sci U S A. 2016;113: 6065–6070. doi:10.1073/pnas.1604478113

13. Jarosz DF, Lancaster AK, Brown JCS, Lindquist S. An evolutionarily conserved prion-like element converts wild fungi from metabolic specialists to generalists. Cell. 2014;158: 1072–1082. doi:10.1016/j.cell.2014.07.024

14. Jarosz DF, Brown JCS, Walker GA, Datta MS, Ung WL, Lancaster AK, et al. Cross-kingdom chemical communication drives a heritable, mutually beneficial prion-based transformation of metabolism. Cell. 2014;158: 1083–1093. doi:10.1016/j.cell.2014.07.025

15. Brown JCS, Lindquist S. A heritable switch in carbon source utilization driven by an unusual yeast prion. Genes Dev. 2009;23: 2320–2332. doi:10.1101/gad.1839109

16. Holmes DL, Lancaster AK, Lindquist S, Halfmann R. Heritable remodeling of yeast multicellularity by an environmentally responsive prion. Cell. 2013;153: 153–165. doi:10.1016/j.cell.2013.02.026

17. Si K, Kandel ER. The Role of Functional Prion-Like Proteins in the Persistence of Memory. Cold Spring Harb Perspect Biol. 2016;8: a021774. doi:10.1101/cshperspect.a021774

18. Si K. Prions: what are they good for? Annu Rev Cell Dev Biol. 2015;31: 149–169. doi:10.1146/annurev-cellbio-100913-013409

19. Rayman JB, Kandel ER. Functional Prions in the Brain. Cold Spring Harb Perspect Biol. 2017;9: a023671. doi:10.1101/cshperspect.a023671

20. Drisaldi B, Colnaghi L, Fioriti L, Rao N, Myers C, Snyder AM, et al. SUMOylation Is an Inhibitory Constraint that Regulates the Prion-like Aggregation and Activity of CPEB3. Cell Rep. 2015;11: 1694–1702. doi:10.1016/j.celrep.2015.04.061

21. Fioriti L, Myers C, Huang Y-Y, Li X, Stephan JS, Trifilieff P, et al. The Persistence of Hippocampal-Based Memory Requires Protein Synthesis Mediated by the Prion-like Protein CPEB3. Neuron. 2015;86: 1433–1448. doi:10.1016/j.neuron.2015.05.021

22. Raveendra BL, Siemer AB, Puthanveettil SV, Hendrickson WA, Kandel ER, McDermott AE. Characterization of prion-like conformational changes of the neuronal isoform of Aplysia CPEB. Nat Struct Mol Biol. 2013;20: 495–501. doi:10.1038/nsmb.2503

23. Heinrich SU, Lindquist S. Protein-only mechanism induces self-perpetuating changes in the activity of neuronal Aplysia cytoplasmic polyadenylation element binding protein (CPEB). Proc Natl Acad Sci U S A. 2011;108: 2999–3004. doi:10.1073/pnas.1019368108

24. Hervas R, Rau MJ, Park Y, Zhang W, Murzin AG, Fitzpatrick JAJ, et al. Cryo-EM structure of a neuronal functional amyloid implicated in memory persistence in Drosophila. Science. 2020;367: 1230–1234. doi:10.1126/science.aba3526

25. Khan MR, Li L, Pérez-Sánchez C, Saraf A, Florens L, Slaughter BD, et al. Amyloidogenic Oligomerization Transforms Drosophila Orb2 from a Translation Repressor to an Activator. Cell. 2015;163: 1468–1483. doi:10.1016/j.cell.2015.11.020

26. Li L, Sanchez CP, Slaughter BD, Zhao Y, Khan MR, Unruh JR, et al. A Putative Biochemical Engram of Long-Term Memory. Curr Biol. 2016;26: 3143–3156. doi:10.1016/j.cub.2016.09.054

27. Li L, McGinnis JP, Si K. Translational Control by Prion-like Proteins. Trends Cell Biol. 2018;28: 494–505. do:10.1016/j.tcb.2018.02.002

28. Majumdar A, Cesario WC, White-Grindley E, Jiang H, Ren F, Khan MR, et al. Critical role of amyloid-like oligomers of Drosophila Orb2 in the persistence of memory. Cell. 2012;148: 515–529. doi:10.1016/j.cell.2012.01.004

29. Mastushita-Sakai T, White-Grindley E, Samuelson J, Seidel C, Si K. Drosophila Orb2 targets genes involved in neuronal growth, synapse formation, and protein turnover. Proc Natl Acad Sci U S A. 2010;107: 11987–11992. doi:10.1073/pnas.1004433107

30. Si K, Choi Y-B, White-Grindley E, Majumdar A, Kandel ER. Aplysia CPEB can form prion-like multimers in sensory neurons that contribute to long-term facilitation. Cell. 2010;140: 421–435. doi:10.1016/j.cell.2010.01.008

31. Hervás R, Li L, Majumdar A, Fernández-Ramírez MDC, Unruh JR, Slaughter BD, et al. Molecular Basis of Orb2 Amyloidogenesis and Blockade of Memory Consolidation. PLoS Biol. 2016;14: e1002361. doi:10.1371/journal.pbio.1002361

32. White-Grindley E, Li L, Mohammad Khan R, Ren F, Saraf A, Florens L, et al. Contribution of Orb2A stability in regulated amyloid-like oligomerization of Drosophila Orb2. PLoS Biol. 2014;12: e1001786. doi:10.1371/journal.pbio.1001786
