# Supplementary material for: Mrj is a chaperone of the Hsp40 family that regulates Orb2 oligomerization and long-term memory in Drosophila
Source: PLoS Biol. 2024 Apr 22;22(4):e3002585. doi: 10.1371/journal.pbio.3002585 (PMC11034981; doi:10.1371/journal.pbio.3002585)
Supplement: Attachment — Submitted filename: Response_editor_March2024.pdf [file pbio.3002585.s015.pdf]

Response to editor's suggestions

Please find our point by point response to the points raised by the editor in blue added next to the editors points.

Dear Amitabha,

Following on from my previous message, thank you again for your patience while we reconsidered our decision regarding your manuscript entitled "Mrj an Hsp40 family chaperone regulates the oligomerization of Orb2 and long-term memory".

Based on our Academic Editor's assessment of your rebuttal, I am pleased to say that we are likely to accept this manuscript for publication, but we think it would be beneficial to include some of your responses to Reviewer #3 in the manuscript text where appropriate. Specifically, we ask that you please include brief clarifications that were provided in response to points 3 and 4, as well citing and contextualizing the previous work noted in response to points 5 and 6 to lend support for your claims.

In the revised manuscript, based on the editorial suggestion we have clarified the following points.

Point 3: We have now added a statement on the quantitation of the intensity of the smear in the SDD-AGE blots.

The statement now reads as:

On probing the SDD-AGE blot with an anti-Orb2 antibody and quantitating the intensity of the smear, we observed a significant increase in Orb2 oligomers in the presence of Mrj.

Point 4: We have added a statement in the figure legend that the lower sized band is most likely a breakdown product. We also have marked in the figure the actual GST-Mrj band in the blot with a *.

The statement in the Figure legend now reads as:

The band marked with * is the GST-Mrj protein and the band below it is possibly a breakdown product from the former.

Point 5: We have a sentence included in the manuscript which cited and contextualized the previous work "SDD-AGE has been previously reported to detect SDS-resistant Orb2 oligomers as a smear in agarose gels (Khan et al, 2015; Li et al, 2016a; Majumdar et al, 2012)."

Point 6: We have added a statement that all the IPs of Mrj with the four Hsp70's were done in the same condition.

The statement now reads as:

All these four immunoprecipitation experiments were done under the same conditions.

We have a sentence in the manuscript which cited and contextualized the previous work, "Mammalian Mrj is reported to interact with Hsp70 proteins (Izawa et al, 2000; Kakkar et al, 2016; Bengoechea et al, 2020)."

Other changes: Based on the first reviewer's suggestion to refer the findings of Mrj's effect on Htt as "Mrj is impedes on the biophysical properties of its aggregating clients" we have now changed the statement as follows:

This intense band probably represents the collapse of the larger Htt oligomers to smaller, less diverse-sized Htt oligomers in the presence of Mrj, suggesting Mrj possibly interferes or impedes HttQ103 aggregation.

Minor changes: We have found some typos and missed some statistical test details in the earlier manuscript and have now changed these.

In addition, please also make sure to address the following data and other policy-related requests that I have provided below (A-F):

(A) We would like to suggest the following modification to the title:

"Mrj is a chaperone of the Hsp40 family that regulates Orb2 oligomerization and long-term memory in Drosophila"

Based on the editorial suggestion we have now changed the title of the manuscript to "Mrj is a chaperone of the Hsp40 family that regulates Orb2 oligomerization and long-term memory in Drosophila"

(B) You may be aware of the PLOS Data Policy, which requires that all data be made available without restriction: http://journals.plos.org/plosbiology/s/data-availability. For more information, please also see this editorial: http://dx.doi.org/10.1371/journal.pbio.1001797

Note that we do not require all raw data. Rather, we ask that all individual quantitative observations that underlie the data summarized in the figures and results of your paper be made available in one of the following forms:

-Supplementary files (e.g., excel). Please ensure that all data files are uploaded as 'Supporting Information' and are invariably referred to (in the manuscript, figure legends, and the Description field when uploading your files) using the following format verbatim: S1 Data, S2 Data, etc. Multiple panels of a single or even several figures can be included as multiple sheets in one excel file that is saved using exactly the following convention: S1_Data.xlsx (using an underscore).

-Deposition in a publicly available repository. Please also provide the accession code or a reviewer link so that we may view your data before publication.

Regardless of the method selected, please ensure that you provide the individual numerical values that underlie the summary data displayed in the following figure panels as they are essential for readers to assess your analysis and to reproduce it:

Figure 1I, 2I, 2K, 2M, 3H-I, 3K, 5D, 5G, 5J, 5L, 6B, 6F, 7E, S4G, S5C, S6A-B

NOTE: the numerical data provided should include all replicates AND the way in which the plotted mean and errors were derived (it should not present only the mean/average values).

We have now provided the requested numerical data in figshare

(https://figshare.com/s/f5d913a0a289339ee16b).

In case we need to make any changes in these regarding the file names, format, etc please let us know. The link here is still a private link and is not published yet.

(C) Please also ensure that each of the relevant figure legends in your manuscript include information on *WHERE THE UNDERLYING DATA CAN BE FOUND*, and ensure your supplemental data file/s has a legend.

We have added the following statement underneath each figure:

The data underlying this figure are available at: https://figshare.com/s/f5d913a0a289339ee16b

(D) We require the original, uncropped and minimally adjusted images supporting all blot and gel results reported in the following Figures:

Figure 1B, 1D, 1H, 1J-K, 2B, 2D, 2F, 2J, 2L, 3B-C, 3J, 4G-K, 4M, 4O-P, 5A, 5C, 5F, 5I, 5K, 6D, 7A-C, 7F-G, S3, S4B, S4E, S5H, S6C-E, S6G, S7A-F

We will require these files before a manuscript can be accepted so please prepare and upload them now. Please carefully read our guidelines for how to prepare and upload this data: https://journals.plos.org/plosbiology/s/figures#loc-blot-and-gel-reporting-requirements

We have now provided the requested numerical data and raw data of all gels and blots in figshare (https://figshare.com/s/f5d913a0a289339ee16b).

In case we need to make any changes in these regarding the file names, format, etc please let us know. The link here is still a private link and is not published yet.

(E) Please ensure that your Data Statement in the submission system accurately describes where your data can be found and is in final format, as it will be published as written there.

We have provided the figshare link in the data statement in the submission system.

(F) Please also provide a blurb which (if accepted) will be included in our weekly and monthly Electronic Table of Contents, sent out to readers of PLOS Biology, and may be used to promote your article in social media. The blurb should be about 30-40 words long and is subject to editorial changes. It should, without exaggeration, entice people to read your manuscript. It should not be redundant with the title and should not contain acronyms or abbreviations. For examples, view our author guidelines: https://journals.plos.org/plosbiology/s/revising-your-manuscript#loc-blurb

We have now added the following blurb to the manuscript:

A biochemistry and behavior based study suggests oligomerization of the prion-like translation regulator Orb2 and long term memory is regulated by a Hsp40 family chaperone protein Mrj.

As you address these items, please take this last chance to review your reference list to ensure that it is complete and correct. If you have cited papers that have been retracted, please include the rationale for doing so in the manuscript text, or remove these references and replace them with relevant current references. Any changes to the reference list should be mentioned in the cover letter that accompanies your revised manuscript.

We have not changed the references apart from changing the format to PLOS Biology format with numbers for references.

We expect to receive your revised manuscript within two weeks.

To submit your revision, please go to https://www.editorialmanager.com/pbiology/ and log in as an Author. Click the link labelled 'Submissions Needing Revision' to find your submission record. Your revised submission must include the following:

- a cover letter that should detail your responses to any editorial requests, if applicable, and whether changes have been made to the reference list

- a Response to Reviewers file that provides a detailed response to the reviewers' comments (if applicable, if not applicable please do not delete your existing 'Response to Reviewers' file.)

- a track-changes file indicating any changes that you have made to the manuscript.

We have included a cover letter responding to the editorial requests, detailed response to the reviewer's comments and a track-changes file indicating the changes made to the manuscript.

NOTE: If Supporting Information files are included with your article, note that these are not copyedited and will be published as they are submitted. Please ensure that these files are legible and of high quality (at least 300 dpi) in an easily accessible file format. For this reason, please be aware that any references listed in an SI file will not be indexed. For more information, see our Supporting Information guidelines:

https://journals.plos.org/plosbiology/s/supporting-information

We have included high quality tiff files for the supplementary information related figures.

Published Peer Review History

Please note that you may have the opportunity to make the peer review history publicly available. The record will include editor decision letters (with reviews) and your responses to reviewer comments. If eligible, we will contact you to opt in or out. Please see here for more details:

https://blogs.plos.org/plos/2019/05/plos-journals-now-open-for-published-peer-review/

We would like the peer review history to be publicly available.

Press

Should you, your institution's press office or the journal office choose to press release your paper, please ensure you have opted out of Early Article Posting on the submission form. We ask that you notify us as soon as possible if you or your institution is planning to press release the article.

Protocols deposition

To enhance the reproducibility of your results, we recommend that if applicable you deposit your laboratory protocols in protocols.io, where a protocol can be assigned its own identifier (DOI) such that it can be cited independently in the future. Additionally, PLOS ONE offers an option for publishing peer-reviewed Lab Protocol articles, which describe protocols hosted on protocols.io. Read more information on sharing protocols at https://plos.org/protocols?utm_medium=editorial-email&utm_source=authorletters&utm_campaign=protocols

Please do not hesitate to contact me should you have any questions.

Sincerely,

Richard

Richard Hodge, PhD

Senior Editor, PLOS Biology

rhodge@plos.org

PLOS

Empowering researchers to transform science

Carlyle House, Carlyle Road, Cambridge, CB4 3DN, United Kingdom

ORCiD I plosbio.org I @PLOSBiology I Blog

California (U.S.) corporation #C2354500, based in San Francisco

---

In compliance with data protection regulations, you may request that we remove your personal registration details at any time. (Use the following URL: https://www.editorialmanager.com/pbiology/login.asp?a=r). Please contact the publication office if you have any questions.
