# Supplementary material for: Mrj is a chaperone of the Hsp40 family that regulates Orb2 oligomerization and long-term memory in Drosophila
Source: PLoS Biol. 2024 Apr 22;22(4):e3002585. doi: 10.1371/journal.pbio.3002585 (PMC11034981; doi:10.1371/journal.pbio.3002585)
Supplement: Attachment — Submitted filename: 2023_05_01 - referee report R1.docx [file pbio.3002585.s013.docx]

The authors have send in a revision in which they mostly adequately dealt with my comments (see below, with my reply in red).

**Figure 1 (plus related Supplemental figures):**

- There seem to be two isoforms of Mrj (like what has been found for human DNAJB6). I find it striking to see that only (preferentially?) the shorter isoform interacts with Orb2. For DNAJB6, the long isoform is mainly related to an NLS and the presumed substrate binding is identical for both isoforms. If this is true for Dm-Mrj too, the authors could actually use this to demonstrate the specificity of their IPs where Orb2 is exclusively non-nuclear?

Well addressed.

- I would be interested to know a bit more about the other 5 JDPs that are interactors with Orb2: are the human orthologs of those known? It is striking that these other 5 JDPs interact with Orb2 in Dm (in IPs) but have no impact on Sup35 prion behavior. Importantly, this does not imply they may not have impact on the prion-like behavior of other Dm substrates, including Dm-Orb2.
- Well addressed. I would like the authors to add the table as supplement to the paper as it is valuable information for follow-up studies. I actually agree that it goes beyond the scope of this paper to show what the biological relevance is of the interaction between these other JDPs and Dm-Orb2.
- The data in panels H,I indeed suggest that Mrj1 alters the (size of) the oligomers. It would be important to know what is the actual physicochemical change that is occurring here. The observed species are insoluble in 0.1 % TX100 but soluble in 0.1% SDS, which suggest they could be gels, but not real amyloids such as formed by the polyQ proteins that require much higher SDS concentrations (~2%) to be solubilized. This is relevant as Mrj1 reduces polyQ amyloidogenesis whereas is here is shown to enhance Orb2A oligomerization/gelidification. In the same context, it is striking to see that without Mrj the amount of Orb2A seems drastically reduced and I wonder whether this might be due to the fact that in the absence of Mrj a part of Orb2A is not recovered/solubilized due to its conversion for a gel to a solid/amyloid state? In other words: Mrj1 may not promote the prion state, but prevents that state to become an irreversible, non-functional amyloid?

I am still a bit confused, also because of the complexity of the answer in relation to my question. I do understand and accept that the authors cannot pinpoint (at this stage) what actual physicochemical states of the Orb2A is in under the various condition. What I still do not understand is why there is less Orb2A in the absence of Mrj1. What I also still think could be the case is that Mrj1 is not driving oligomerization per se, but that its (early) interactions with Orb2A affect the oligomerization process (up until becoming amyloids). So, I suggest that the authors remain somewhat open-minded about this alternative explanation and mention it in their paper.

**Figure 2 (plus related Supplemental figures):**

- It may be good for clarity to refer to the human Mrj as DNAJB6 according to the HUGO nomenclature. Also, the first evidence for its oligomerization was by Hageman et al 2010.

Well addressed.

- It is striking to see that Mrj co-IPs with Hsp70AA, Hsp70-4 but not Hsp70Cb. The fact that interactions were detected without using crosslinking is also striking given the reported transient nature of J-domain-Hsp70 interactions Together, this may even suggest that Mrj-1 is recognized as a Hsp70 substrate (for Hsp70AA, Hsp70-4 but not Hsp70Cb) rather than as a co-chaperone. In fact, a variant of Mrj-1 with a mutation in the HPD motif should be used to exclude this option.

I understand and am aware of the fact that in some cases JDP-Hsp70 interactions are seen in co-IP even without crosslinking, but also here these may be indirect via binding to substrates for which different Hsp70 may have different specificities (rather than Mrj-1 specifically binding to specific Hsp70 isoforms). In any case, I feel an experiment with an HDP motif mutant would be essential in my view to make any claims on J-domains showing specific binding to only a subset of Hsp70 isoforms.

- The rest of these data reconfirm nicely that Mrj/DNAJB6 can suppress polyQ-Htt aggregation. Yet note that in this case the oligomers that enter the agarose gel are smaller, not bigger. This argues that Mrj is not an enhancer of oligomerization, but rather an inhibitor of the conversion of oligomers to a more amyloid like state.

I was indeed wrong regarding my interpretation of figure 2I (I assume incorrectly that the lane marked with Mrj was the knockout). However, the interpretation still is (even more so now) skewed by the fact that the total amount of polyQ detected in the case of cells with Mrj is so much less that in the controls. So, whereas the other anti-aggregation data for polyQ are still solid and confirming literature data, I still feel one cannot interpret that data as to suggest that Mrj is an “enhancer of oligomerization” (for neither polyQ, nor for Orb2A: see above). The situation is likely more subtle.

**Figure 3:**

- The finding that knockout of DNAJB6 in mice is embryonic lethal is related to a problem with placental development and not embryonic development (Hunter et al, 1999; Watson et al, 2007, 2009, 2011) as well recognized by the authors. Therefore, the finding that deletion of Dm-Mrj has no developmental phenotype in Drosophila may not be that surprising.

Well addressed.

- It is a bit more surprising that Mrj knockout flies showed no aggregation phenotype or muscle phenotype, especially knowing that DNAJB6 mutations are linked to human diseases associated with aggregation (again well recognized by the authors). However, most of these diseases are late-onset and the phenotype may require stress to be revealed. So, while important to this MS in terms of not being a confounder for the memory test, I would like to ask the authors to add a note of caution that their data do not exclude that loss of Mrj activity still may cause a protein aggregation-related disease phenotype. Yet, I also do think that for the main message of this MS, it is not required to further test this experimentally.

Well addressed.

**Figure 4:**

- IPs were done with Orb2A as bite and clearly pulled down substantial amounts of GFP-tagged Mrj. For interactions with Orb2B, a V5-tagged Mrj was use and only a minor fraction was pulled down. Why were two different Mrj constructs used for Arb2A and Orb2b?

Well addressed.

- In addition, I think it would be important what one would see when pulling on Mrj1, especially under non-denaturing conditions and what is the status of the Orb2 that is than found to be associated with Mrj (monomeric, oligomeric and what size).

Well addressed: nice additional data!.

- This also relates to my remark at figure 1 and the subsequent fractionation experiments they show here in which there is a slight (not very convincing) increase in the ratio of TX100-soluble and insoluble (0.1% SDS soluble) material. My question would be if there is a remaining fraction of 0.1% insoluble (2% soluble) Orb2 and how Mrj affects that? As stated before, this is (only) mechanistically relevant to understanding why there is less oligomers of Orb2 in terms of Mrj either promoting it or by preventing it to transfer from a gel to a solid state. The link to the memory data remains intriguing, irrespective of what is going on (but also on those data I do have several comments: see below).

Well addressed (still see comments above).

- I also find the sentence that “Mrj is probably regulating the oligomerization of endogenous Orb2 in the brain” somewhat an overstatement. I would rather prefer to say that the data show that Mrj1 affects the oligomeric behavior/status of Orb2.

Well addressed.

**Figure 5:**

- To my knowledge, the Elav driver regulates expression in all neurons, but not in glial cells that comprise a significant part of the fly heads/brain. The complete absence of Mrj in the fly-heads when using this driver is therefore somewhat surprising. Or do we need to conclude from this that glial cells normally already lack Mrj expression?

Well addressed.

- Why not use these lines also for the memory test for confirmation? I understand the concerns of putative confounding effects of a full knockdown (which were however not reported), but now data rely only on the mushroom body-specific knockdown for the 201Y Gal4 line, for which the knockdown efficiency is not provided. But even more so, here a temperature shift (22oC-30oC) was required to activate the expression of the siRNA. For the effects of this shift alone no controls were provided. The functional memory data are nice and consistent with the hypothesis, but can it be excluded that the temperature shift (rather than the Mrj) knockdown has caused the memory defects? I think it is crucial to include the proper controls or use a different knockdown approach that does not require temperature shifts or even use the knockout flies.

Well addressed. Nice new, consistent data!

**Figure 6:**

The finding of a co-IP between Rpl18 and Mrj (one-directional only) by no means suffices to conclude that Mrj may interact with nascent Orb2 chains here (which would be the relevant finding here). The fact that Mrj is a self-oligomerising protein (also in vitro, so irrespective of ribosomal associations!), and hence is found in all fractions in a sucrose gradient, also is not a very strong case for its specific interaction with polysomes. The finding that there is just more self-oligomerizing Orb2A co-sedimenting with polysomes in sucrose gradients neither is evidence for a direct effect of Mrj enhancing association of Orb2A with the translating ribosomes even though it would fit the hypothesis. So all in all, I think the data in this figure and non-conclusive and the related conclusions should be deleted.

Well addressed with nice additional data.

Overall, provided that proper controls/additional data can be provided for the key experiments of memory consolidation, I find this an intriguing study that would point towards a role of a molecular chaperone in controlling memory functions via regulating the oligomeric status of a prion-like protein and that is worthwhile publishing in a good journal.

However, in terms of mechanistical interpretations, several points have to be reconsidered (see remarks on figure 1,4); this pertains especially to what is discussed on page 13. In addition, I’d like the authors to put their data into the perspective of the findings that in differentiated neurons DNAJB6 levels actually decline, not incline (Thiruvalluvan et al, 2020), which would be counterintuitive if these proteins are playing a role as suggested here in memory consolidation.

See my remaining comments above. I think a slight rephrasing of a few of the interpretations would suffice for me to advise acceptation of this nice MS.
